# Introgression maintains the genetic integrity of the mating-type determining chromosome of the fungus *Neurospora tetrasperma*

**DOI:** 10.1101/gr.197244.115

**Published:** 2016-04

**Authors:** Pádraic Corcoran, Jennifer L. Anderson, David J. Jacobson, Yu Sun, Peixiang Ni, Martin Lascoux, Hanna Johannesson

**Affiliations:** 1 Department of Organismal Biology, Uppsala University, 752 36 Uppsala, Sweden;; 2 Department of Animal and Plant Sciences, University of Sheffield, Sheffield S10 2TN, United Kingdom;; 3 Department of Cell and Molecular Biology, Uppsala University, 752 36 Uppsala, Sweden;; 4 BGI HongKong;; 5 Department of Ecology and Genetics, Science for Life Laboratory, Uppsala University, 752 36 Uppsala, Sweden

## Abstract

Genome evolution is driven by a complex interplay of factors, including selection, recombination, and introgression. The regions determining sexual identity are particularly dynamic parts of eukaryotic genomes that are prone to molecular degeneration associated with suppressed recombination. In the fungus *Neurospora tetrasperma*, it has been proposed that this molecular degeneration is counteracted by the introgression of nondegenerated DNA from closely related species. In this study, we used comparative and population genomic analyses of 92 genomes from eight phylogenetically and reproductively isolated lineages of *N. tetrasperma*, and its three closest relatives, to investigate the factors shaping the evolutionary history of the genomes*.* We found that suppressed recombination extends across at least 6 Mbp (∼63%) of the mating-type (*mat*) chromosome in *N. tetrasperma* and is associated with decreased genetic diversity, which is likely the result primarily of selection at linked sites. Furthermore, analyses of molecular evolution revealed an increased mutational load in this region, relative to recombining regions. However, comparative genomic and phylogenetic analyses indicate that the *mat* chromosomes are temporarily regenerated via introgression from sister species; six of eight lineages show introgression into one of their *mat* chromosomes, with multiple *Neurospora* species acting as donors. The introgressed tracts have been fixed within lineages, suggesting that they confer an adaptive advantage in natural populations, and our analyses support the presence of selective sweeps in at least one lineage. Thus, these data strongly support the previously hypothesized role of introgression as a mechanism for the maintenance of mating-type determining chromosomal regions.

The content, structure, and organization of eukaryote genomes change over time in response to complex interactions between selection, mutation, recombination, introgression, and other factors. Chromosomal regions conferring sexual identity (i.e., sex or mating type) are particularly dynamic parts of eukaryote genomes, evolving independently and divergently from a formerly homologous state (Bull 1983). This divergent evolution is associated with suppressed recombination between the chromosomes that effectively preserves their unique identities. Low, or suppressed, recombination leads to a reduction in effective population size (*N*_e_) through selection at linked sites; thereby the efficacy of selection is reduced and we expect an increased mutational load (Bachtrog and Charlesworth 2002; Charlesworth and Charlesworth 2010). The link between recombination suppression and molecular degeneration has been observed in the sex- and mating-type determining genomic regions of a number of taxa across all eukaryote kingdoms (e.g., Bachtrog 2003; Hood et al. 2004; Liu et al. 2004; Marais et al. 2008; Whittle and Johannesson 2011; Whittle et al. 2011a; Fontanillas et al. 2015) and is one of the main factors expected to favor either rare recombination events on sex chromosomes (Malcom et al. 2014) or a high turnover of chromosomes harboring the sex-determining loci (Blaser et al. 2013).

In the filamentous ascomycete, *Neurospora tetrasperma*, it has been proposed that introgression, the integration of genetic material from one species into the genome of another, serves to renew and maintain the integrity of the mating-type determining chromosomal regions (Sun et al. 2012). This species has independently evolved pseudohomothallism, a mating system in which self-fertility is achieved through the production of sexual spores that contain nuclei of both mating types (i.e., it is heterokaryotic for *mat A* and *mat a*) (Supplemental Fig. 1; Raju and Perkins 1994). Pseudohomothallism in *N. tetrasperma* is thought to have evolved from a heterothallic ancestor, for which sexual spores are of single mating type (Supplemental Fig. 1), about one million years ago (Corcoran et al. 2014). To accomplish correct nuclear packaging in the spores of *N. tetrasperma*, crossing over is suppressed between the *mat* locus and the centromere, ensuring that *mat A* and *mat a* will segregate at the first division of meiosis. Indeed, in *N. tetrasperma*, recombination is suppressed across most of the mating-type (*mat*) chromosome in all strains examined to date (Howe and Haysman 1966; Merino et al. 1996; Gallegos et al. 2000; Menkis et al. 2008; Ellison et al. 2011b). In the *Neurospora* genus, this recombination suppression is unique to *N. tetrasperma* and is not found in its heterothallic sister taxa, for which the *mat* chromosomes freely recombine except in a very short region (3–5 kb) surrounding the *mat* locus (Glass et al. 1990; Staben and Yanofsky 1990). Furthermore, in *N. tetrasperma*, suppressed recombination is accompanied by an accumulation of nonbeneficial mutations (Ellison et al. 2011b; Whittle and Johannesson 2011; Whittle et al. 2011a; Sun et al. 2012). Sun et al. (2012) used comparative genomics of six *N. tetrasperma* strains to show that introgression of the *mat* chromosomes from other freely recombining *Neurospora* species may have reduced degeneration on the *mat a* chromosomes. Here, we use a large-scale genomic sampling of 92 genomes and a population and comparative genomic approach to evaluate the roles of selection, recombination, and introgression in shaping the *mat* chromosomes of *N. tetrasperma* populations*.*

## Results

### Global pattern of variation in *N. tetrasperma*

**Figure 1.**
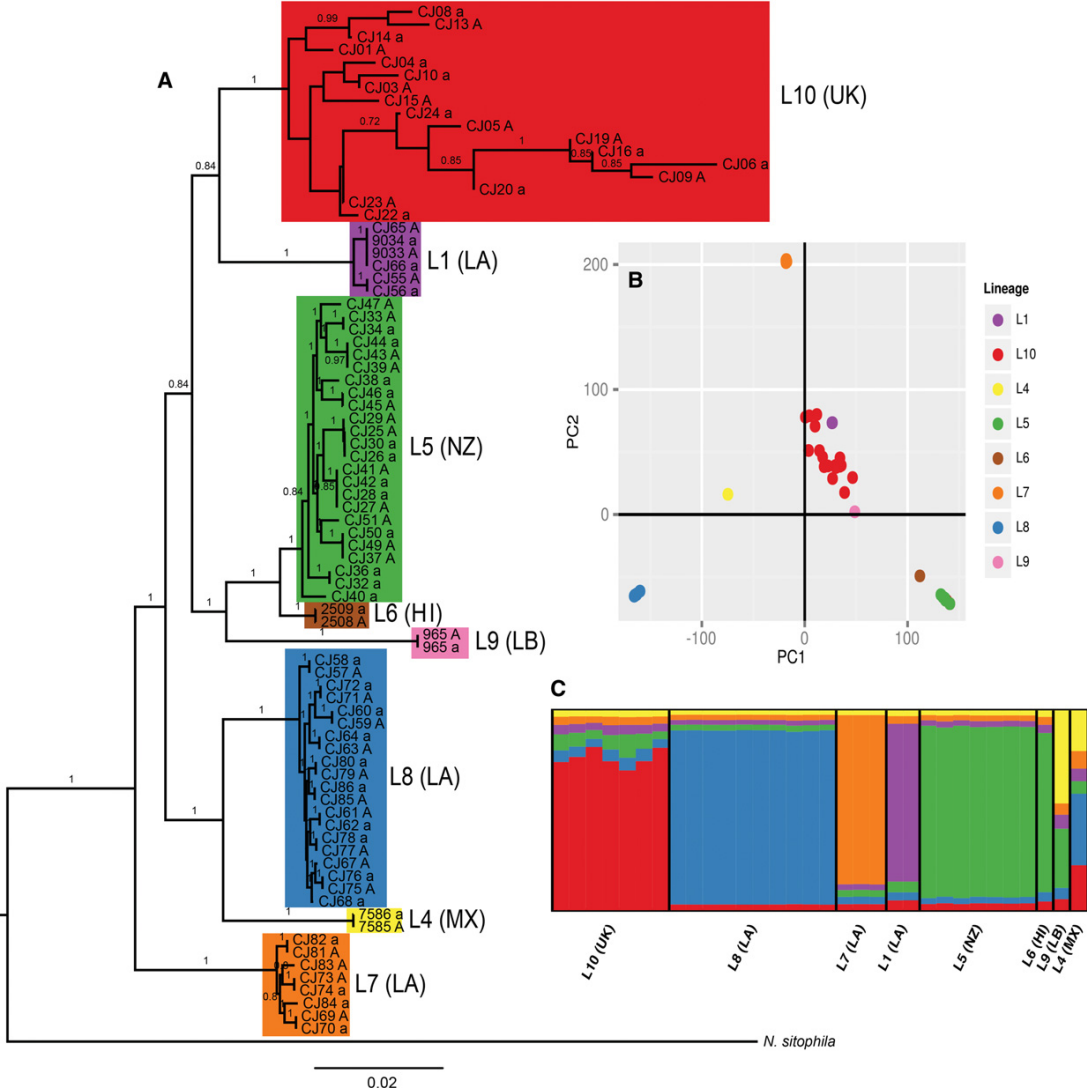
The global pattern of variation in *N. tetrasperma.* (*A*) The phylogenetic relationships of all *N. tetrasperma* strains used in this study, inferred from 2,259,433 variable sites on the autosomes. A subtree excluding *N. discreta*, *N. crassa*, and *N. hispaniola* is shown. Numbers on the branches indicate the bootstrap support for that relationship expressed as a proportion. (*B*) Principal component analysis (PCA) of genetic variation (509,199 biallelic autosomal SNPs) across the global sample of *N. tetrasperma* strains. The first two principal components are shown. (*C*) Population structure of *N. tetrasperma* inferred from 9000 SNPs (1500 from each of the six autosomes) using InStruct at *K* = 6. Lineages color coded in *A*, *B*, and *C* according to the legend in *B*. (LA) Louisiana; (NZ) New Zealand; (UK) United Kingdom; (HI) Hawaii; (MX) Mexico; (LB) Liberia.

Genome sequencing (to mean coverage of 25–45×) and reference assembly of 92 strains of *N. tetrasperma* from across the globe (Supplemental Table 1) resulted in the discovery of 1,693,770 biallelic single nucleotide polymorphisms (SNPs) within this clade. After filtering of heterokaryotic strains and clones (Supplemental Fig. 2; Supplemental Table 1), we analyzed the autosomes (the set of six chromosomes corresponding to linkage groups [LG] II to VII in *N. crassa*) to reveal the global pattern of variation in *N. tetrasperma*. The largest chromosome, the *mat* chromosome (linkage group I in *N. crassa*), was excluded due to the large regions of suppressed recombination on this chromosome in *N. tetrasperma*. All strains of *N. tetrasperma* form a monophyletic group, as confirmed by both Maximum Likelihood phylogenomic analysis of variable sites and a species tree inference of autosomal gene trees (Fig. 1A; Supplemental Fig. 3). Furthermore, for the first time, we show with strong phylogenetic support that *N. sitophila* is the sister taxon of *N. tetrasperma* (Supplemental Fig. 3; cf. Dettman et al. 2003; Corcoran et al. 2014). Additionally, phylogenomic and principal component analyses confirm the previously defined lineages of *N. tetrasperma* (Fig. 1A,B; Corcoran et al. 2014), henceforth, referred to as L1 to L10. In accordance with previous studies (Saenz et al. 2003; Menkis et al. 2009; Corcoran et al. 2014), lineages primarily correlate with geographical region, although this pattern is not universal, for example, the strains of *N. tetrasperma* from Louisiana (LA) belong to three genetically divergent lineages (L1, L7, and L8) (Fig. 1A,B). Despite lineages of the *N. tetrasperma* clade constituting well-supported phylogenetic groups (Fig. 1A), Bayesian clustering analysis on a randomly chosen subset of 9000 autosomal SNPs indicates that the genomic ancestry of only five of the lineages belongs to one population (Fig. 1C). L4, L9, and L10 show mosaic ancestries, which may result from past hybridization between lineages, or in the case of L4 and L9, may reflect the inability to assign their ancestry to a single population given the small sample sizes for these lineages (Fig. 1C; Supplemental Fig. 4).

### A history of selfing and admixture in *N. tetrasperma*

**Figure 2.**
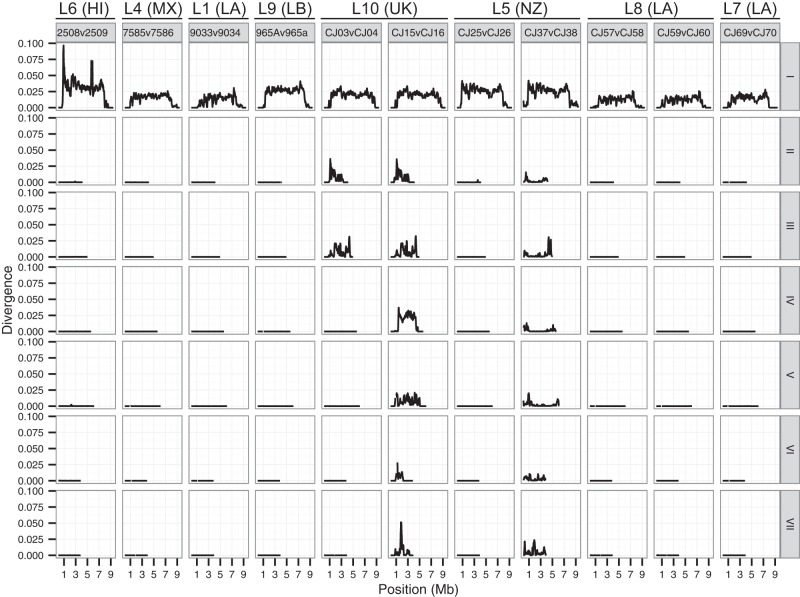
Pair-wise divergences between the *mat A* and *mat a* homokaryons sampled from the same heterokaryon for the *mat* chromosomes (linkage group I) and six autosomes (II–VII) of representatives from all *N. tetrasperma* lineages. Each linkage group is shown in a separate row (labeled on the *right*). The pair-wise divergences were calculated as the fraction of differences (in bp) between the sequences, using a 100-kb sliding window (step size 20 kb). (LA) Louisiana; (NZ) New Zealand; (UK) United Kingdom; (HI) Hawaii; (MX) Mexico; (LB) Liberia.

Our analyses strengthen the view of *N. tetrasperma* as a predominantly selfing species. First, linkage disequilibrium (LD) is much more extensive in all *N. tetrasperma* lineages than previously determined for populations of the heterothallic close relative *N. crassa* (Supplemental Table 2; Ellison et al. 2011a). The levels of LD observed in L5 and L8 extend for 11 and 31 kb, respectively; and in L10, LD extends for hundreds of kilobases on some chromosomes (Supplemental Figs. 5, 6)*.* Second, few differences were found across the autosomes of *mat A* and *mat a* homokaryons (i.e., strains containing nuclei of a single mating type) isolated from the same natural heterokaryon (Fig. 2; Supplemental Fig. 7), and the vast majority of such paired mating-type homokaryons (e.g., CJ57 *A* and CJ58 *a* from L8) group together in the phylogeny of Figure 1A.

However, the allelic distribution of two autosomal heterokaryon incompatibility (*het*) genes supports a history of occasional outcrossing in *N. tetrasperma*. These genes govern self–nonself recognition in natural fungal populations and typically evolve under balancing selection and therefore maintain ancestral polymorphism through speciation (e.g., Powell et al. 2007). We found different alleles of *het* genes among closely related strains of *N. tetrasperma* and shared alleles in distantly related lineages (Supplemental Figs. 8, 9), a pattern inconsistent with obligate selfing in the history of *N. tetrasperma* (cf. Powell et al. 2001; Menkis et al. 2009).

A notable exception to the pattern of phylogenetic grouping of paired autosomes derived from natural heterokaryons is L10, in which homokaryon pairs originating from natural heterokaryons do not cluster together (Fig. 1 A) and strains are highly divergent (Fig. 2; Supplemental Fig. 7). Many of the chromosome pairs within heterokaryons of L10 are extensively differentiated from each other (Fig. 2; Supplemental Fig. 7), a pattern which is particularly striking on LG IV, with a chromosomal divergence of >2% (Fig. 2; Supplemental Fig. 7). High genetic variation (Supplemental Table 2), an excess of intermediate frequency variants across the genome (Supplemental Fig. 10), and extensive linkage disequilibrium (Supplemental Figs. 5, 6) are consistent with a history of recent admixture in L10.

**Table 1.**
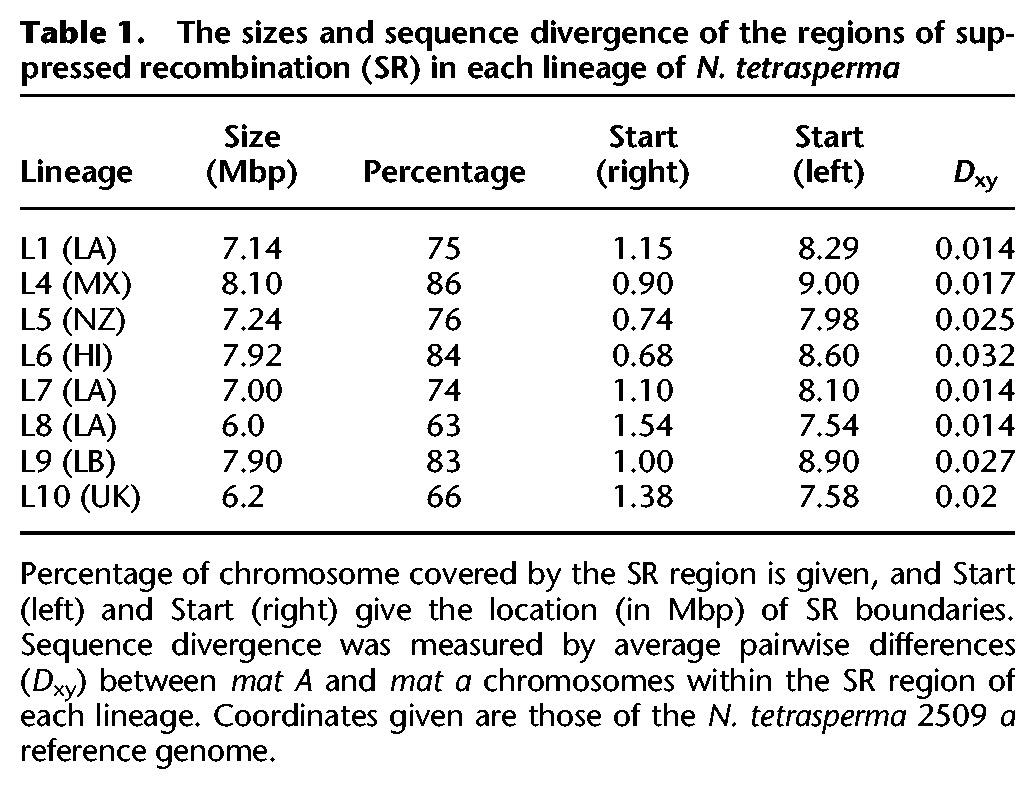
The sizes and sequence divergence of the regions of suppressed recombination (SR) in each lineage of *N. tetrasperma*

### Recombination suppression and reduced diversity of the *mat* chromosomes

**Figure 3.**
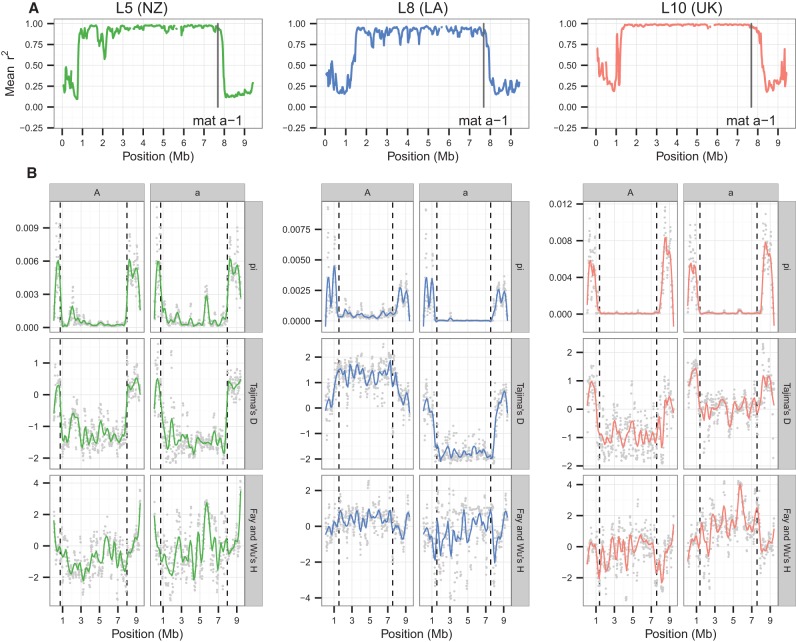
Patterns of genetic variation across the *mat* chromosome for *N. tetrasperma* lineages L5, L8, and L10. (*A*) Linkage disequilibrium given as the mean Pearson's correlation coefficient (*r*^*2*^). The vertical black line shows the position of the *mat a* locus in the 2509 reference genome. (*B*) Nucleotide diversity (π), Tajima's *D* (Tajima 1989), and Fay and Wu's *H* (Fay and Wu 2000). For all variables, we used a 100-kb window size (step size 20 kb). The values for each window are represented by the gray points, and smooth lines were plotted with stat_smooth in the ggplot2 R package using the gam method with a span of 0.2. Dashed vertical lines indicate lineage-specific limits of the SR region.

In *N. tetrasperma*, unlike its heterothallic sister taxa, recombination is suppressed between the *mat* locus and the centromere. Without recombination, genetic material on homologous chromosomes is expected to diverge. Indeed, for all strains of *N. tetrasperma* studied here, pairs of *mat A* and *mat a* chromosomes originating from the same heterokaryons harbor large regions of elevated divergence, a pattern in stark contrast to most of the autosomes (Fig. 2; Supplemental Fig. 7). Furthermore, all SNPs within this region in L5, L8, and L10 (lineages with sample sizes allowing for population level analyses) were found to be in near complete LD (Fig. 3A), indicating that recombination is absent in this region.

Our results show that the extent of divergence between *mat A* and *mat a* chromosomes varies significantly across lineages (Kruskal-Wallis test, *P* < 0.001) (Table 1; Fig. 2; Supplemental Figs. 7, 11). Divergence between the SR regions of *mat A* and *mat a* is highest in L6 at 3.2%, which is more than twofold greater than the lowest divergence of 1.4% observed in L8 (Table 1; Supplemental Fig. 11). The region of the reference genome corresponding to the SR regions also vary in size, ranging from 6 Mbp for L8, to 8.1 Mbp for L4, which translates into 63%–86% of the entire *mat* chromosome, the largest chromosome in this species (Table 1). Note that the given sizes of the SR regions are relative to the reference genome assembly; actual sizes of the SR regions may differ in cases in which an individual genome differs from the reference, but are not possible to assess with these data.

As a direct effect of recombination suppression, we expect *N*_e_ of the *mat* chromosomes to be reduced to at least half of that of the autosomes (cf. Kimura 1983; Charlesworth and Charlesworth 2000), and selection at linked sites is expected to further reduce *N*_e_ in this region of the genome. Accordingly, the diversity of all investigated *N. tetrasperma* lineages is greatly reduced in the SR region compared to recombining chromosomal (R) regions. Specifically, when analyzing the synonymous nucleotide diversity (π_s_) in the SR and R regions, we found that they differ by >75-fold in L10, up to ∼24-fold in L8, and up to ∼fivefold in L5 (Supplemental Fig. 12; Supplemental Table 3).

### Widespread occurrence of *mat* chromosome introgressions in *N. tetrasperma*

**Figure 4.**
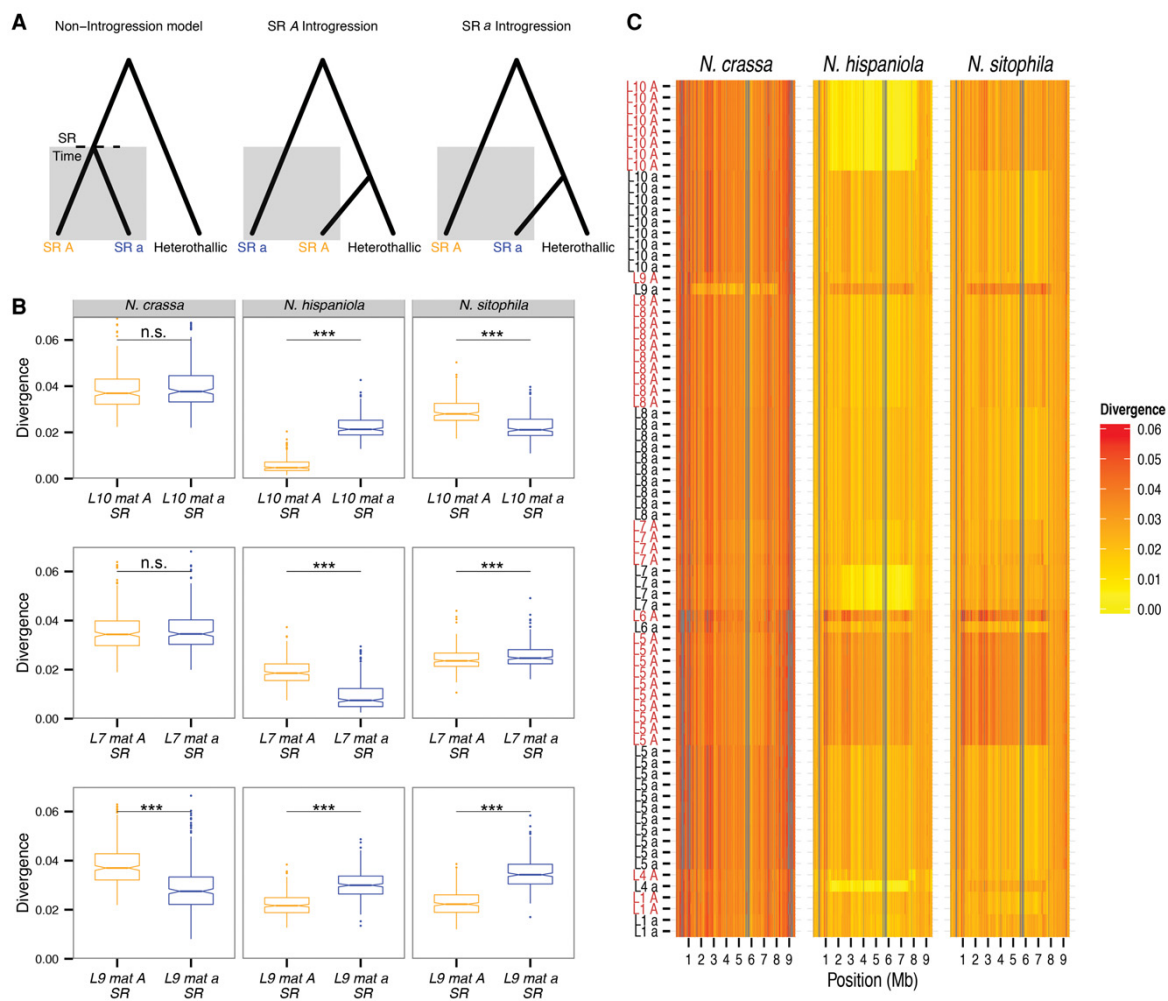
Introgression of large chromosomal regions from heterothallic *Neurospora* to *N. tetrasperma*. (*A*) Diagrams showing the genealogy of the *mat* chromosome SR region in a *N. tetrasperma* lineage when suppression of recombination had begun at SR Time. The shaded regions show that a lineage is in SR. The models from *left* to *right* show expected relationships in the absence of introgression, SR *A* introgression, and SR *a* introgression. (*B*) Box plots of mean divergence between L10, L7, and L9 in the SR region. Asterisks *above* the horizontal black lines are the *P*-values for the Mann-Whitney *U* test between SR regions within a lineage: (***) *P* < 0.001; (n.s.) nonsignificant. (*C*) Pair-wise divergences between the *mat* chromosomes of *N. tetrasperma* strains and the heterothallic species *N. crassa, N. hispaniola*, and *N. sitophila*. Each row in the figure shows the sequence divergence between a strain of *N. tetrasperma* and the heterothallic species indicated in the heading of the column, using a nonoverlapping sliding window of 25 kb. *N. tetrasperma* strains are sorted by lineage and according to mating type. Regions lacking a sufficient number of sites (2500 sites) are colored gray. A maximum divergence of up to 0.06 is plotted, and windows exceeding this are colored gray.

**Figure 5.**
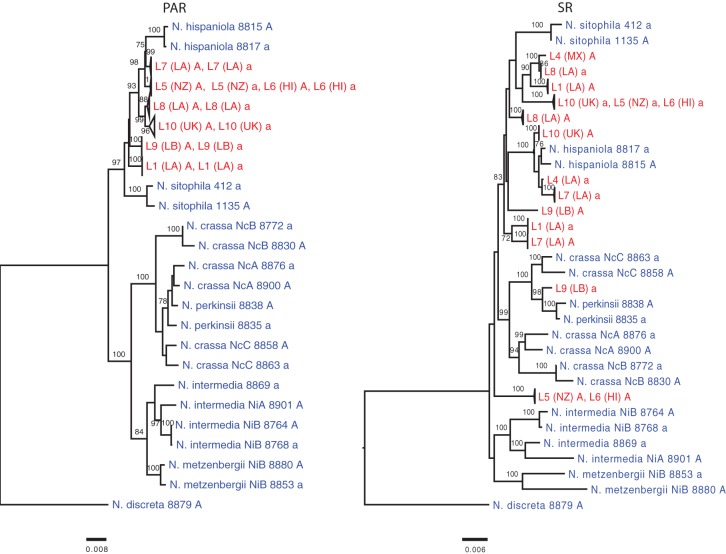
The phylogenetic relationships of *Neurospora* strains in the PAR and SR regions common to all *N. tetrasperma* lineages. The SR tree was reconstructed from a concatenated data set of six genes and the PAR tree from two genes (see Supplemental Table 12). The heterothallic species are in blue, and *N. tetrasperma* lineages in red. Numbers on the branches are bootstrap support values expressed as a percentage; values below 70 are not shown.

Hybridization of *N. tetrasperma* with other heterothallic species is predicted to leave long tracts of introgression in the SR regions of the *N. tetrasperma mat* chromosomes due to the lack of recombination to break them up over time (Fig. 4A). Using genomic scans of divergence between *N. tetrasperma* lineages and heterothallic species, and phylogenetic analysis of *mat* chromosome genes, we found evidence that introgression into the *mat* chromosomes has occurred in six of the eight investigated lineages of *N. tetrasperma* (Table 2). Moreover, introgressions have originated from multiple species of *Neurospora*: At least three heterothallic species appear to have been donors to *N. tetrasperma* (Table 2; Fig. 5; Supplemental Table 4).

**Table 2.**
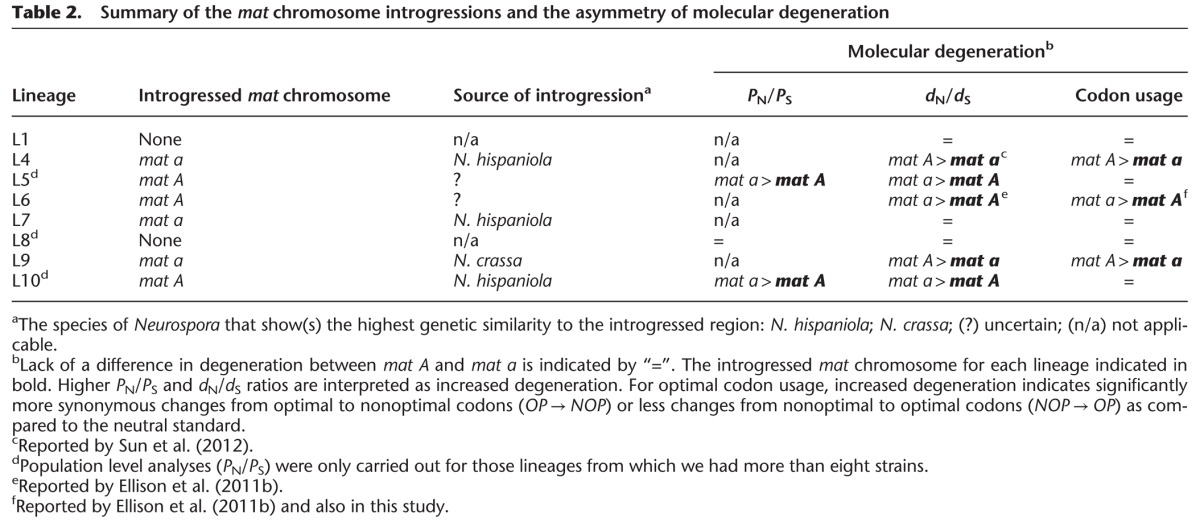
Summary of the *mat* chromosome introgressions and the asymmetry of molecular degeneration

Typically, divergence in the SR regions is higher within than between lineages of *N. tetrasperma* (Supplemental Fig. 13), suggesting that these regions of the genome have not diverged through mutations alone. When comparing the genomes of *N. tetrasperma* strains to genomes of the heterothallic species *N. crassa*, *N. hispaniola*, and *N. sitophila*, we observed cases in which one of the SR regions (in either the *A* or *a* homokaryon) is significantly more similar to a heterothallic species than the opposite *mat* SR and R regions for that strain (Fig. 4B; Supplemental Fig. 14), suggesting that these regions have been introgressed from other species. This pattern is notable in comparisons between L10A (i.e., the *mat A* chromosome of lineage 10), L4a, and L7a and *N. hispaniola*, and between L9a and *N. crassa* (Fig. 4B; Supplemental Fig. 13; Supplemental Table 4). The SR regions of these chromosomes are visible as long tracts of low divergence to one or the other of the investigated heterothallic species (Fig. 4C; Supplemental Fig. 14). Also, in L10, for which population level analyses are possible, we found that *mat A* chromosomes share a large excess of derived alleles with *N. hispaniola* within the SR region, further supporting introgression from *N. hispaniola* in this lineage (Supplemental Fig. 16). In the *mat A* SR regions of L5 and L6, our analyses show that the divergence from *N. hispaniola* and *N. sitophila* are higher than the other strains of *N. tetrasperma* (Fig. 4C), suggesting that they have been introgressed from more distantly related species.

To test whether the regions of low sequence divergence across the *mat* chromosome (Fig 4 C) can be explained by variance in coalescent times across the genome due to drift in the ancestral population of the species being compared, we simulated sequence data for species pairs under a multispecies coalescent model without gene flow, using the MCMCcoal program (Rannala and Yang 2003). If the regions of low divergence are due to introgression, they should be extreme outliers compared to the divergence calculated from the simulated data. Using this approach, we found that the simulations could not reproduce the low levels of sequence divergence that we observe between L10A, L4a, and L7a and *N. hispaniola*, providing additional support for the hypothesis that these SR regions have been introgressed (Supplemental Table 4), whereas a pure isolation model, with no introgression, can explain the levels of divergence observed across comparisons for the other lineages (Supplemental Table 4). Interestingly, for L9a, the divergence to *N. crassa* genome within the SR region is not significantly different from that expected under a model without gene flow (*P* = 0.25) (Supplemental Table 4), a result that may be due to the donor species being distant from *N. crassa*, as supported by the phylogenetic analysis outlined below.

Phylogenetic analysis of six genes in the SR region and two genes from the pseudoautosomal regions (PAR: the recombining flanking regions of the *mat* chromosome), show that L10A, L4a, and L7a all group closely with the *N. hispaniola* strains, further supporting that these regions have been introgressed from *N. hispaniola*. Furthermore, L9a is closest to *N. perkinsii* for the SR region (Fig. 5) (0.5% divergence at the investigated genes), a species close to *N. crassa* but not included in the comparative genomic analysis shown in Figure 4, whereas the heterothallic species closest to L5A and L6A could not be identified with certainty.

The PAR has a pattern similar to the autosomal phylogenetic tree with *A* and *a* strains from the same lineage forming clades. However, *N. tetrasperma* lineages do not form a monophyletic group in the PAR. This may be due to introgression or incomplete lineage sorting, but few conclusive explanations can be drawn from the analysis of two genes.

### Evidence of autosomal introgression supports interspecific hybridization in *Neurospora*

Genome-wide analyses of sequence divergence to the heterothallic *Neurospora* species show that large tracts of introgressed DNA are mainly restricted to the *mat* chromosome of *N. tetrasperma* (Supplemental Fig. 16). However, we found evidence that the autosomes of *N. tetrasperma* lineages have patterns consistent with a history of gene flow with their heterothallic relatives. We observe long tracts of low divergence to *N. hispaniola* in the center of LG IV (Supplemental Fig. 16) in four strains of L10. These tracts correspond to large regions of elevated divergence between the homokaryons isolated from those heterokaryons (Fig. 2; Supplemental Fig. 7). The diverged regions are much larger than the expected centromeric regions in *Neurospora*, and the specific tracts mentioned above do not include the centromere (Sun et al. 2012), and thus cannot be explained by maintenance of heterozygosity of centromeric regions under automixis (Hood and Antonovics 2000). This long tract on LG IV may instead be due to intogression into L10 being more recent than for the other lineages of *N. tetrasperma*.

To specifically test for a history of introgression on the *N. tetrasperma* autosomes, we used three approaches: Patterson's D-statistics (Green et al. 2010), TreeMix analysis (Pickrell and Pritchard 2012), and a likelihood ratio test of post divergence gene flow with the program 3S (Yang 2010). The D-statistics were calculated for the autosomes using “ABBA-BABA” site patterns using the genomic data available from heterothallic species. Using this approach, we found that all L10 strains show signatures of autosomal introgression from *N. hispaniola* (significantly positive D-statistic for all strains (Supplemental Table 5) with L1 strain 9033 as P1, L10 as P2, *N. hispaniola* as P3, and *N. discreta* as the outgroup), whereas no such signal was detected when using *N. sitophila* or *N. crassa* as P3 (Supplemental Tables 6, 7, respectively). Lineages 4, 7, and 8 also show signatures of autosomal introgression from *N. hispaniola* (Supplemental Table 5), consistent with the patterns of introgression found on the *mat* chromosomes of L4 and L7 (Table 2).

We used a Treemix analysis to reconstruct evolutionary relationships of *N. tetrasperma* from autosomal SNPs and to see whether a history of migration between lineages gives a better fit to the data than a tree-like history based on visual inspection of the plot of residuals (Supplemental Fig. 17). The addition of migration events to the model improves the fit to the data (Supplemental Fig. 17B,C). With the addition of five migration events, we found support for a history in which L10 is an admixed lineage with past migration from L4 and *N. hispaniola* (Supplemental Fig. 17C) and for gene flow from L7 to *N. hispaniola*.

Finally, the likelihood ratio test of gene flow implemented with the 3S program indicated that the divergence model, including gene flow between the heterothallic species and *N. tetrasperma* lineages, fits the data better than a pure isolation model for all comparisons involving *N. hispaniola* and *N. sitophila* for the autosomes. A model without gene flow shows a better fit to the data for all comparisons with *N. crassa*, with the exception of L9, L1, and L4, in which the model with gene flow is preferred (Supplemental Table 8).

### Introgression reduces mutational load in regions of suppressed recombination

Suppression of recombination and reduced *N*_e_ are expected to lead to a decrease in the efficacy of natural selection and to contribute to the degeneration of regions determining sexual identity (Charlesworth and Charlesworth 2000). As outlined below, we observe a pattern of mutation accumulation consistent with a reduced efficacy of selection in the SR region within each lineage of *N. tetrasperma*. Our analyses also show population level support for the hypothesis that introgression from species that do not suffer from SR-related mutational load acts to reduce the mutational load on *N. tetrasperma mat* chromosomes (Table 2).

We assayed mutational load in the *N. tetrasperma* genomes by estimating three parameters: (1) the ratio of nonsynonymous polymorphisms per nonsynonymous site to synonymous polymorphisms per synonymous site (*P*_N_/*P*_S_); (2) the nonsynonymous/synonymous substitution rate (*d*_N_/*d*_S_); and (3) the switches from optimal to nonoptimal synonymous codons. The accumulation of nonsynonymous polymorphisms/substitutions and nonoptimal codons are typically interpreted as molecular degeneration in regions with reduced recombination (Betancourt et al. 2009; Whittle et al. 2011a). In L5, L8, and L10, the *P*_N_/*P*_S_ ratio is significantly higher in all SR than R regions (Fisher's exact test [FET], *P* < 0.001 for all SR v R comparisons) (Supplemental Table 9), a result consistent with a reduced *N*_e_. In L10 and L5, the introgressed *mat A* regions have a significantly lower *P*_N_/*P*_S_ than the *mat a* (FET, L10: *P* < 0.001 and L5 *P* < 0.05) (Supplemental Table 9), whereas in L8 (not introgressed), *P*_N_/*P*_S_ between the *mat* chromosomes is not significantly different (Table 2; Supplemental Fig. 18).

Analyses of substitution rates confirm the prediction of a lower nonsynonymous/synonymous substitution rate (*d*_N_/*d*_S_) within the introgressed SR regions of the *mat* chromosomes (Table 2). Specifically, in the introgressed L7a and L9a regions, we observed lower *d*_N_/*d*_S_ ratios compared to genes in the *mat A* SR region of the same lineages, although this was only significant in L9 (Mann-Whitney *U* test [MWU], *P* = 0.055 and *P* < 0.001 for L7 and L9, respectively) (Supplemental Table 10). In the cases of L10 and L5, the introgressed *mat A* SR regions had significantly lower *d*_N_/*d*_S_ than the *mat a* (MWU, *P* < 0.05 and *P* < 0.001 for L5 and L10, respectively), indicating less degeneration in the introgressed *mat A* chromosomes. For L6 and L4, which were excluded from this analysis due to high similarity to other lineages, the introgressed *mat* region was previously confirmed to exhibit a lower *d*_N_/*d*_S_ ratio than its nonintrogressed counterparts (Ellison et al. 2011b; Sun et al. 2012). In the two lineages for which introgression was not confirmed in this study (L1 and L8), we found no significant differences in *d*_N_/*d*_S_ ratio between the *mat* chromosomes (Table 2). The result for L1 differs from that reported by Sun et al. (2012), who found that the *mat A* chromosome was more degenerated than the *mat a* counterpart in this lineage. The lack of a significant difference in this study may be due to the much larger number of genes and taxa included in the analysis.

The higher *d*_N_/*d*_S_ values of the nonintrogressed *mat* chromosomes in *N. tetrasperma* is unlikely to be the result of a history of positive selection acting specifically at these sites in these lineages, because the efficacy of selection is reduced, as indicated by an elevated *P*_N_/*P*_S,_ within the SR regions (see above) (Supplemental Fig. 18). This reduction in efficacy of selection may result in an elevated *d*_N_/*d*_S_ due to the fixation of deleterious nonsynonymous mutations.

Finally, analyses of codon usage also support the notion that introgressed regions have a lower level of degeneration: The introgressed L9a and L6A both exhibit a significant excess of switches from nonoptimal to optimal codons relative to the nonintrogressed *mat* chromosome in those lineages (FET L6 *P* < 0.001; L9 *P* = 0.0003). The number of switches to nonoptimal codons is lower in the introgressed L4a relative to L4A (FET *P* < 0.05) (Table 2; Supplemental Table 11). However, no significant differences in switches from optimal to nonoptimal and vice versa were observed for the other lineages, in which introgression of one of the *mat* chromosomes has occurred (Table 2; Supplemental Table 11).

### Models of linked selection on the *mat* chromosomes

The large SR regions on the *mat* chromosomes of *N. tetrasperma* should be susceptible to the effects of both selective sweeps and background selection (Charlesworth and Charlesworth 2010). We calculated Tajima's D and Fay and Wu's H statistics within the SR regions of L5, L8, and L10 to test for selective sweeps. Tajima's D statistic (*D*) can detect an excess of low frequency variants following a selective sweep in a region, whereas Fay and Wu's H statistic (*H*) can detect the excess of high frequency derived mutations that remain following a recent sweep (Fig. 3B; Tajima 1989; Fay and Wu 2000; Zeng et al. 2006). These statistics were calculated within a lineage on the *mat A* and *mat a* SR regions separately and support the history of selective sweeps on the nonintrogressed L8a region and the introgressed L10A region. Specifically, the SR region of most of the *mat* chromosomes in this analysis (i.e., L5A and L5a, L8a and L10A) shows a negative *D* within the SR region (Fig. 3), indicating an excess of low frequency polymorphisms in the regions. However, only L8a has a significantly negative *D* for the SR region (*D* = −2.078, *P* < 0.0001 from 10,000 coalescent simulations without recombination).

A recent selective sweep event within an SR region should leave a signature of negative *H* values in the flanking recombining regions. Hence, in order to identify regions that were subject to recent hard selective sweeps, we divided the genome in nonoverlapping 100-kb windows to detect regions of the genome with most extreme (top 5%) negative *H*. Using this approach, we observed two adjacent windows of extreme negative *H* ∼300 kb outside the L8a SR boundary between position 7.8–8.0 Mb, and a 100-kb window ∼100 kb outside the L10A SR boundary from position 7.7–7.8 Mb.

To test further for signatures of selective sweeps, we carried out coalescent simulations for a single hard sweep model in a nonrecombining region and determined that such a hard selective sweep model does not fit the data for the majority of the SR regions examined. The observed measures of neutral diversity from the SR regions fit well with a large range of values of two parameters varied in the sweep model—*T*_s_ (the time since the sweep occurred) and θ_0_ (the neutral variation present in the absence of a sweep)—for the L10 and L5 SR regions (Supplemental Fig. 19). However, the L8a SR region fits a much narrower range of parameter values for the sweep model than the other SR regions, with a narrow range of *T*_s_ that are more recent than observed in the other SR regions (Supplemental Fig. 19). These data are also consistent with values of θ_0_ in the model that overlap with the levels of neutral diversity observed on the autosomes. The observation that a selective sweep model only fits one (L8a) of the six SR regions examined here suggests that selective sweeps are difficult to detect or may be rare in the SR regions of *N. tetrasperma*.

## Discussion

In this study, we extend previous genomic studies of *N. tetrasperma* (e.g., Sun et al. 2012) to include population level analyses of genomes from multiple lineages within the species and provide novel insights into the complex interactions between recombination, introgression, and selection in natural eukaryote populations. In particular, we extend the understanding of factors driving the evolution of nonrecombining genomic regions by showing that adaptive introgression contributes to evolutionary transitions of chromosomal regions determining mating type, and this has the potential to generate cycles of reinvigoration following accumulation of deleterious mutations. First, we found that suppressed recombination of the *mat* chromosome is associated with decreased genetic diversity. Although such low diversity is commonly found for nonrecombining sex chromosomes in animals and plants (Bachtrog and Charlesworth 2002; Hellborg and Ellegren 2004; Laporte et al. 2005), this provides, to our knowledge, the first demonstration of a reduction in genetic variation in nonrecombining genomic regions conferring sexual identity in fungi (cf. Votintseva and Filatov 2011). Second, we found that introgression has been a common phenomenon in the history of *N. tetrasperma* and played an important role in the evolution of the *mat* chromosomes of this species. Large tracts have been introgressed from multiple heterothallic species into both *mat A* and *mat a* chromosomes of *N. tetrasperma*; thus, introgression is not biased toward *mat a* chromosomes as indicated by the smaller scale study by Sun et al. (2012). Structural heterozygosity due to rearrangements of the *mat A* chromosome of the *N. tetrasperma* reference strain (FGSC 2508) has been reported previously (Jacobson 2005; Ellison et al. 2011b). If rearrangements are the sole cause of recombination suppression in this system, and the changes found on the *mat A* chromosome in FGSC 2508 are ancestral in *N. tetrasperma*, we would expect long introgression tracts to be confined to the *mat a* chromosomes throughout the species (cf. Sun et al. 2012). However, the data presented herein do not support this hypothesis. In the absence of chromosome-level de novo assemblies of the *N. tetrasperma* genomes analyzed in this study, we are not able to infer the gene order and architecture of the *mat* chromosomes of the strains investigated here and are thus unable to connect structural heterozygosity with recombination suppression and introgression in *N. tetrasperma*.

Two possible alternative explanations to introgression for the patterns of low divergence to heterothallic species are balancing selection and ancestral variance in coalescent times. Mating-type loci in fungi have been shown to evolve under balancing selection, and alleles within these regions can be more similar to the same allele from a different species than a different allele of the same species (e.g., May et al. 1999). However, since recombination is not suppressed around the *mat* locus in heterothallic *Neurospora*, this pattern should be confined to the *mat* locus and not extend to encompass most of the flanking regions of the *mat* chromosome in heterothallic species and is unlikely to produce the observed low levels of sequence divergence. We can also discount ancestral variance in coalescent times as an explanation for the low divergence we observe in L10A, L4a, and L7a to *N. hispaniola,* and L9a to *N. perkinsii*.

The observation of large introgressed blocks from multiple heterothallic species, fixed in *N. tetrasperma* lineages, supports the view that adaptive introgression has contributed to the evolutionary history of *N. tetrasperma.* A history of recombination on the *mat* chromosomes of heterothallic “donor” species has acted to purge deleterious mutations by an effective purifying selection. Therefore, we hypothesize that fixation of an introgressed haplotype by positive selection occurred because it contained fewer linked deleterious mutations than the other *N. tetrasperma* haplotypes already present in the recipient lineage.

It is noteworthy that we did not detect the signature of recent large-scale selective sweeps on the introgressed SR regions of L5A and L10A; thus, our data suggest that the alternative model of background selection may be a better model for explaining the patterns of molecular evolution in *N. tetrasperma* SR regions for these lineages. This finding, however, does not necessarily argue against the adaptive introgression model, as the action of background selection occurring after the fixation of the introgressed region could have removed any signatures of older sweep events. The fact that the SR regions of *N. tetrasperma* are relatively young (∼1 million years old) compared to sex chromosomes or other neo-sex chromosomes (Zhou and Bachtrog 2012; Charlesworth 2013; Cortez et al. 2014) and that the majority of the genes in this SR region are expressed (Samils et al. 2013) suggests that purifying selection may be a potent force in these regions. Also, the fact that *N. tetrasperma* is viable in a haploid homokaryotic state suggests that deleterious mutations in the SR regions, that may otherwise be masked from selection in a heterokaryon, are subject to periods of haploid selection. Taken together, the view that emerges of the history of linked selection in the *mat* SR regions of *N. tetrasperma* is one of a genomic region that is exposed to negative background selection and shaped by occasional bouts of positive selection of introgressed tracts.

Recombination suppression on the chromosome carrying the mating-type locus has evolved independently in several fungal species distantly related to *N. tetrasperma*, including *Podospora, Cryptococcus*, and *Microbotryum* (Fraser and Heitman 2004; Grognet et al. 2014; Fontanillas et al. 2015) and contributes to our understanding of different aspects of eukaryote genome evolution. Signatures of molecular degeneration associated with recombination suppression have been reported in *Neurospora* and *Microbotryum* (Hood et al. 2004; Fontanillas et al. 2015). There are massive changes in architecture on the *Microbotryum* mating-type chromosome (Badouin et al. 2015), a pattern also reported from one of the *N. tetrasperma* strains (Ellison et al. 2011b), and further studies using high quality genomic data have the potential to reveal the commonality of such rearrangements and their link to recombination suppression of fungal *mat* chromosomes. In summary, this study shows the value of fungal *mat* chromosomes to provide insights into factors driving eukaryote genome evolution by providing a new model for how to evade the inevitable cost of recombination suppression on chromosomes determining sexual identity.

## Methods

### Genome sequencing and assembly of *Neurospora* strains used in the study

We selected 94 strains of *Neurospora* for genome sequencing: 92 strains of *N. tetrasperma*, and one each of the two species *N. sitophila* and *N. hispaniola* (Supplemental Table 1). These taxa differ in mating system—*N. tetrasperma* is pseudohomothallic, and *N. sitophila* and *N. hispaniola* are heterothallic (see Supplemental Fig. 1 for details). All strains were obtained from, or have been deposited to, the Fungal Genetics Stock Center (FGSC), University of Missouri, Kansas City. The majority of the sequenced strains were single mating-type homokaryons (*mat A* and *mat a*) originating from mating-type heterokaryons sampled in nature (Supplemental Fig. 1; Supplemental Table 1). We sequenced strains identified previously as homokaryotic for mating type by laboratory crosses and PCR screens (Corcoran et al. 2012). We then verified mating type and homokaryosis, as outlined in Supplemental Methods, and used only verified strains in further analyses.

Genomic DNA was extracted from mycelial tissue using the Easy-DNA Kit (Invitrogen). Paired-end library preparation and whole-genome sequencing was carried out at BGI HongKong using Illumina HiSeq 2000, which generated an average of 1.5 Gbp of paired 90-bp reads per strain (an average of 1.4 Gbp of sequenced reads after quality filtering) and an average coverage per strain of between 25 and 45× (Supplemental Table 1). The quality filtering of the FASTQ files was performed by BGI HongKong as described in Sun et al. (2015). We performed both reference and de novo genome assemblies of the reads. Detailed procedures for read mapping to two available reference genomes of *N. tetrasperma* (2508 and 2509) (Ellison et al. 2011b), variant calling, and de novo assembly is outlined in the Supplemental Methods.

### Genome alignments of outgroup heterothallic species

Whole-genome alignments of the *N. tetrasperma*, *N. crassa*, and *N. discreta* reference genomes were performed using Mauve v2.3.1 (Supplemental Methods; Darling et al. 2010).

### Phylogenomic analyses

We used two approaches to resolve the phylogenetic relationships of the *Neurospora* species and lineages included in this study. In the first analysis, we carried out Maximum Likelihood phylogenomic analysis using an alignment of only variable sites from all autosomes using RAxML v7.3.1 (Stamatakis 2006). The second analysis used the STAR method (Liu et al. 2009) for estimating species trees from a collection of rooted gene trees from genes on the autosomes. Gene trees from 5723 genes were used as input for STAR (Supplemental Methods).

### Population structure analyses

The Bayesian clustering program InStruct (Gao et al. 2007) was used to analyze the population structure within the *N. tetrasperma* clade. We used 1500 randomly chosen SNPs from each of the autosomes for which we had complete genotype information (i.e., no missing data) across all strains, resulting in a total set of 9000 SNPs. InStruct was run with *K* = 1–12, and five replicate runs for each *K*, where *K* is the number of ancestral clusters to which an individual can be assigned (Supplemental Methods).

A principal component analysis (PCA) was performed to further investigate the genetic relationships among our global sample of *N. tetrasperma* strains. We performed the PCA using Adegenet (Jombart and Ahmed 2011) on 509,119 biallelic SNPs from autosomes from which we had data for every strain.

### Determining the region of suppressed recombination (SR)

The region of suppressed recombination (SR) on the *mat* chromosome of *N. tetrasperma* was determined by two approaches. The first used comparisons between the level of linkage disequilibrium (LD) on the *mat* chromosome and the autosomes to demarcate the SR region. This method was used for L5, L8, and L10, the lineages for which we had the largest number of strains (more than eight). LD between SNPs was calculated using the square of Pearson's correlation coefficient (*r*^2^) as implemented in the Python module egglib v2.1.5 (De Mita and Siol 2012). The *r*^2^ for all chromosomes was calculated by sliding across the chromosomes in 100-kb windows with a step size of 20 kb. The mean *r*^2^ value for each window was estimated by calculating the mean of the *r*^2^ for all pairwise combinations of SNPs within a window. We used the 95% quantile for *r*^2^ across the autosomes as a cutoff to identify the SR region on the *mat* chromosome (i.e., windows with *r*^2^ above this cutoff on the *mat* chromosome were assigned to the SR).

The second approach to determine the SR region on the *mat* chromosome was used for lineages in which the sample size was small (eight or fewer) and was the same as used by Sun et al. (2012) (Supplemental Methods).

### Patterns of genetic variation within *N. tetrasperma* lineages

To quantify the levels of variation within *N. tetrasperma* lineages, we calculated the nucleotide diversity (π) (Tajima 1983) across the genome. We also analyzed the frequency spectrum of mutations across the genomes using Tajima's D statistic (Tajima 1989) and Fay and Wu's H statistic (Fay and Wu 2000; Zeng et al. 2006) using egglib v2.1.7 (De Mita and Siol 2012). For the analysis of the autosomes of L5 and L8, we randomly chose a single homokaryon from each heterokaryon because the vast majority of heterokaryons had few heteroallelic differences on the autosomes. L10 was excluded from these analyses because high levels of diversity were observed within heterokaryons.

The decay of linkage disequilibrium in the genome was examined in lineages L5, L8, and L10. Linkage disequilibrium between pairs of SNPs as measured by *r*^2^ was calculated using egglib. The mean *r*^2^ was calculated for all SNPs within a particular physical distance at increasing 1-kb increments (i.e., the mean *r*^2^ between pairs of SNPs within 1, 2, 3 kb, and so on until 500 kb).

### Genetic distance between pairs of *N. tetrasperma* homokaryons

We calculated genetic distance as the proportion of nucleotide differences per nucleotide site between the *mat A* and *mat a* genomes of each heterokaryon in sliding windows of 100 kb with a 20-kb step. The observed values of genetic distance were corrected for multiple hits using the Juke-Cantor correction (Jukes and Cantor 1969). Windows that had fewer than 10,000 called sites (10%) were excluded.

### Divergence between *N. tetrasperma* lineages

The level of divergence between lineages was measured using the *D*_xy_ statistic (Nei and Li 1979), the average number of pairwise differences between populations. The divergence was measured in 25-kb nonoverlapping windows across the genomes. Sites with missing data were excluded in the calculation of *D*_xy_. Windows with fewer than 2500 (10%) called sites were excluded.

### Divergence between *N. tetrasperma* lineages and heterothallic species

Here, we calculated the divergence between each strain, or each lineage, of *N. tetrasperma* and the genomes of heterothallic species: *N. crassa*, *N. hispaniola*, and *N. sitophila*. The Jukes-Cantor corrected divergence (Jukes and Cantor 1969) between each *N. tetrasperma* strain and each heterothallic species was calculated across the genome in 25-kb nonoverlapping windows. Windows with fewer than 2500 (10%) called sites were excluded.

### Tests for introgression between species using D-statistics

To test for introgression from heterothallic species into the autosomes of the *N. tetrasperma* lineages, we calculated the D-statistic (aka the “ABBA-BABA” test) as outlined by Green et al. (2010) (Supplemental Methods). Block-jackknife resampling with a block size of 250 kb was used to estimate the standard error of the D-statistic and to calculate a *Z*-score. The *Z*-score was then converted to a *P*-value to determine statistical significance of the D-statistic.

To visualize the pattern of ABBA and BABA sites on the *mat* chromosome, we took a sliding window approach that considered only sites fixed between the *mat A* and *mat a* mating-type chromosome from the same lineage (e.g., L10A and L10a) (Supplemental Methods).

### Treemix analysis

We performed an analysis on autosomal SNPs with the program Treemix (Pickrell and Pritchard 2012) to reconstruct the population history of the *N. tetrasperma* lineage incorporating any history of migration between lineages or any migration from *N. sitophila* or *N. hispaniola* using the following parameters: *-k 10000 -noss -root N. hispaniola*. The program was run with different numbers of migration events (-m0 to -m10).

### Likelihood ratio test of divergence with gene flow in autosomes

We used a likelihood-based approach to test for gene flow between a heterothallic species and lineages of *N. tetrasperma* using the program 3S (Yang 2010). The model implemented in the 3S program requires at least one genome per species. We chose *N. discreta* as the outgroup for all triplets of species. We chose the *N. tetrasperma* genome with the highest coverage within a lineage as the representative genome for that lineage. We examined all combinations of *N. tetrasperma* lineages and heterothallic species pairs. We used a likelihood ratio test, as described by Yang (2010), to test whether we could reject the M_0_ isolation model in favor of the M_1_ model with post divergence gene flow (Supplemental Methods).

### Simulations of sequence divergence between species

To determine if the regions of low divergence that we observed in the SR regions of some lineages is due to introgression, or can be better explained by variance in coalescent times due to genetic drift in the ancestral population of a pair species, we performed simulations using the MCMCcoal program. The ancestral population sizes parameter values (θ_4_ and θ_5_) and speciation times parameter values (τ_0_ and τ_1_) used were taken from the results for the M_0_ model in Supplemental Table 8. The parameters estimated for the different *N. tetrasperma* lineages were similar when the same heterothallic was used. Therefore, we calculated the median from the range of estimates as our input parameter value for MCMCcoal (e.g., for θ_5_ in ((*N. tetrasperma*, *N. hispaniola*), *N. discreta*)), the values ranged from 0.016997 to 0.019756. We used the median from this range of values as our choice for θ_5_. We used the same divergence time to *N. discreta* in all simulations (see Supplemental Methods for parameter values used).

We simulated 50,000 25-kb regions without migration between species and calculated the divergence between Species A and B (Supplemental Methods) and compared them to our observed median divergence value within a *mat* SR for a lineage. The *P*-value was calculated as the proportion of simulated divergence values that were equal to or more extreme (greater or less than, depending on whether the SR region being considered showed higher or lower divergence to Species B than the R region of the same lineage) than the observed median divergence of a lineage's SR region to a heterothallic species.

### Phylogenetic analysis of the genes on the *mat* chromosome

We performed maximum likelihood phylogenetic analysis of genes within the SR region and the PAR region of the *mat* chromosome using RAxML v7.3.1. We selected six genes from the SR region and two genes from the PAR common to all lineages of *N. tetrasperma* and included representatives from all heterothallic species (Supplemental Table 12; Supplemental Methods).

### Analyses of molecular evolution in coding regions

We investigated the levels of polymorphism and divergence in coding regions of genes listed in the *N. tetrasperma* 2509 annotation file that had an ortholog in *N. crassa* and *N. discreta* (Supplemental Methods). For polymorphism analysis of coding regions, we retained only the genes that had a minimum length of 70% of the full-length coding sequence, as specified in the *N. tetrasperma* 2509 genome annotation file. We calculated the number of polymorphisms at nonsynonymous (*P*_N_) and synonymous sites (*P*_S_) for each gene within lineages L5, L8, and L10. We also calculated π for both synonymous and nonsynonymous polymorphisms. All counts of coding sites and calculation of polymorphism statistics were carried out using methods from the bio++ library (Guéguen et al. 2013) through egglib (De Mita and Siol 2012).

We used the codeml program in PAML v4.3 (Yang 2007) to test hypotheses on the ratio of nonsynonymous to synonymous substitution rates within the SR region of each *N. tetrasperma* lineage. We carried out the codeml analysis on individual gene alignments of genes within the SR region common to all lineages of *N. tetrasperma*. We removed codons from gene alignments in which there were polymorphisms segregating within a lineage. These were removed because for the codeml analysis, we were only interested in investigating the rates of nonsynonymous and synonymous substitutions that have occurred in the SR regions of each lineage. Following the filtering of polymorphic sites, we retained only a single sequence from each lineage in our gene alignments. We required that the gene alignment length covers at least 70% of the complete CDS sequence for a gene as defined in the *N. tetrasperma* 2509 annotation file. These steps resulted in 1133 genes being used for the codeml analysis. Because estimates of *d*_N_/*d*_S_ are unreliable when branch lengths are short, we excluded L4, L5a, and L6 from the analyses: These lineages showed very low synonymous sequence divergence with other lineages and were analyzed for asymmetrical substitution rates in previous studies (Ellison et al. 2011b; Whittle and Johannesson 2011; Whittle et al. 2011a; Sun et al. 2012). The free ratio model was implemented in codeml to estimate *d*_N_/*d*_S_ for each branch in the phylogeny.

### Codon usage analysis

We further investigated the signal of molecular degeneration in the SR regions of each lineage of *N. tetrasperma* by examining patterns of synonymous substitutions. For this analysis, we also used the polymorphism filtered gene set (described above) for each lineage. We used a Perl script to run through all codon positions for each three-way alignment, including the coding sequence of *N. discreta*, *N. tetrasperma mat A* and *mat a*. We took an optimal (OP) and nonoptimal (NOP) codon list from previous published studies of codon usage study in *N. tetrasperma* and *N. discreta* (Whittle et al. 2011a,b). We assumed the codons from *N. discreta* represent the ancestral state; thus, synonymous codon changes in one of the two *N. tetrasperma* genomes represent allele-specific derived changes. We further divided the allele-specific codon changes into four categories for *N. tetrasperma mat A* and *mat a*: OP to OP, OP to NOP, NOP to OP, and NOP to NOP. The changes of OP to OP and NOP to NOP were used as neutral changes.

### Fit of selective sweep model to SR regions

We tested if a recent hard selective sweep could explain the patterns of reduced diversity of the *mat* SR regions of *N. tetrasperma* by fitting a hard selective sweep model to neutral variation from SR regions of L5, L8, and L10. We followed the approach outlined in Jensen et al. (2002) and used in a number of studies (e.g., Bachtrog 2004; Betancourt et al. 2009; Campos et al. 2014) to model a hard selective sweep in a genomic region without recombination. This approach to modeling a selective sweep is accomplished by performing neutral coalescent simulations without recombination that proceed backward in time until a time, *T*_s_, when all the lineages in the sample are forced to coalesce to a single node, which represents the effect of a selective sweep. Two parameters were varied in the model: (1) the amount of neutral variation (θ_0_) that would be present in the absence of a sweep; and (2) the time (*T*_s_) since the sweep took place. We performed 25,000 replicate simulations for each parameter pair combination. We considered θ_0_ and *T*_s_ pairs compatible with the data if the simulated number of segregating sites (*S*) was within ± (0.05 × *S*) of the observed *S* and the simulated average number of pairwise differences (*k*) was within ± (0.05 × *k*) of the observed *k*. The likelihood of the data was calculated for each parameter pair as described by Jensen et al. (2002). For our observed measures of neutral variation within SR regions of L5, L8, and L10, we used only fourfold degenerate sites and calculated *S* and *k* using polydNdS (Thornton 2003).

### Phylogenetic analysis of heterokaryon incompatibility (*het*) genes

We extracted the *het-6* and *het-c* gene sequences from the de novo assemblies of the *N. tetrasperma*, *N. sitophila*, and *N. hispaniola* genomes (Supplemental Methods). We aligned the *het-c* sequences with the *het-c* sequences from Hall et al. (2010) and *het-6* sequenced from Powell et al. (2007). We constructed phylogenetic trees from the resulting *het-c* and *het-6* alignments using PhyML v3.0.

## Data access

FASTQ files generated in this study have been submitted to the NCBI Sequence Read Archive (SRA; http://www.ncbi.nlm.nih.gov/sra/) under accession number SRP040006. The VCF files, consensus sequences for the *N. tetrasperma* strains, *N. hispaniola* 8817, and *N. sitophila* 1135, whole-genome alignments, and all gene alignments used for the phylogenetic analysis are available from the Dryad Digital Repository (https://datadryad.org/resource/doi: 10.5061/dryad.162mh).

## Supplementary Material

Supplemental Material
