# Supplementary material for: Introgression maintains the genetic integrity of the mating-type determining chromosome of the fungus Neurospora tetrasperma
Source: Genome Res. 2016 Apr;26(4):486–98. doi: 10.1101/gr.197244.115 (PMC4817772; doi:10.1101/gr.197244.115)
Supplement: Supplemental Material [file supp_26_4_486__index.html]

Introgression maintains the genetic integrity of the mating-type determining chromosome of the fungus Neurospora tetrasperma — Introgression maintains the genetic integrity of the mating-type determining chromosome of the fungus Neurospora tetrasperma — Supplemental Material 

# Introgression maintains the genetic integrity of the mating-type determining chromosome of the fungus *Neurospora tetrasperma*

## Supplemental Material

**Files in this Data Supplement:**

- Supplemental Figures.pdf
- Supplemental Methods.pdf
- Supplemental Tables.pdf
