# Supplementary material for: Introgression maintains the genetic integrity of the mating-type determining chromosome of the fungus Neurospora tetrasperma
Source: Genome Res. 2016 Apr;26(4):486–98. doi: 10.1101/gr.197244.115 (PMC4817772; doi:10.1101/gr.197244.115)
Supplement: Supplemental Material [file supp_gr.197244.115_Supplemental_Figures.pdf]

Supplementary Figures

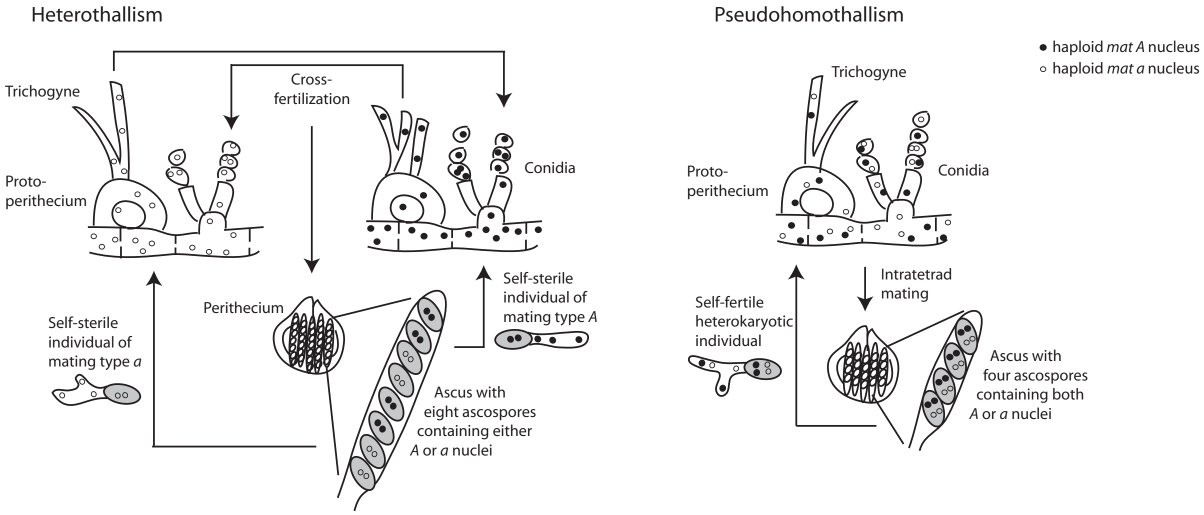

Supplementary Figure 1. Schematic illustration of heterothallism and pseudohomothallism: two mating systems found in Neurospora. Heterothallic species (such as *N. crassa*, *N. hispaniola* or *N. sitophila*) form eight-spored asci with spores homokaryotic for mating type (i.e., they contain haploid nuclei of only one mating-type: *mat A* or *mat a*). These ascospores give rise to homokaryotic, and self-sterile, mycelia, which need to cross-fertilize in order to complete the life cycle. The pseudohomothallic species *Neurospora tetrasperma* forms four-spored asci with ascospores that are heterokaryotic for mating-type (i.e., they contain haploid nuclei of both mating-types: *mat A* and *mat a*). The ascospores germinate to form heterokaryotic, and self-fertile, mycelia. Heterokaryosis is the predominant nuclear stage in natural strains of *N. tetrasperma*. Occassionally, however, *N. tetrasperma* produces sexual or asexual spores (conidia) that are homokaryotic for mating-type. These give rise to self-sterile mycelia that need to outcross in order to mate and reestablish pseudohomothallim. Homokaryotic cultures of *N. tetrasperma* can be grown by the isolation of spores that contain only one kind of nucleus. All genomes analyzed in this study were from homokaryotic cultures.

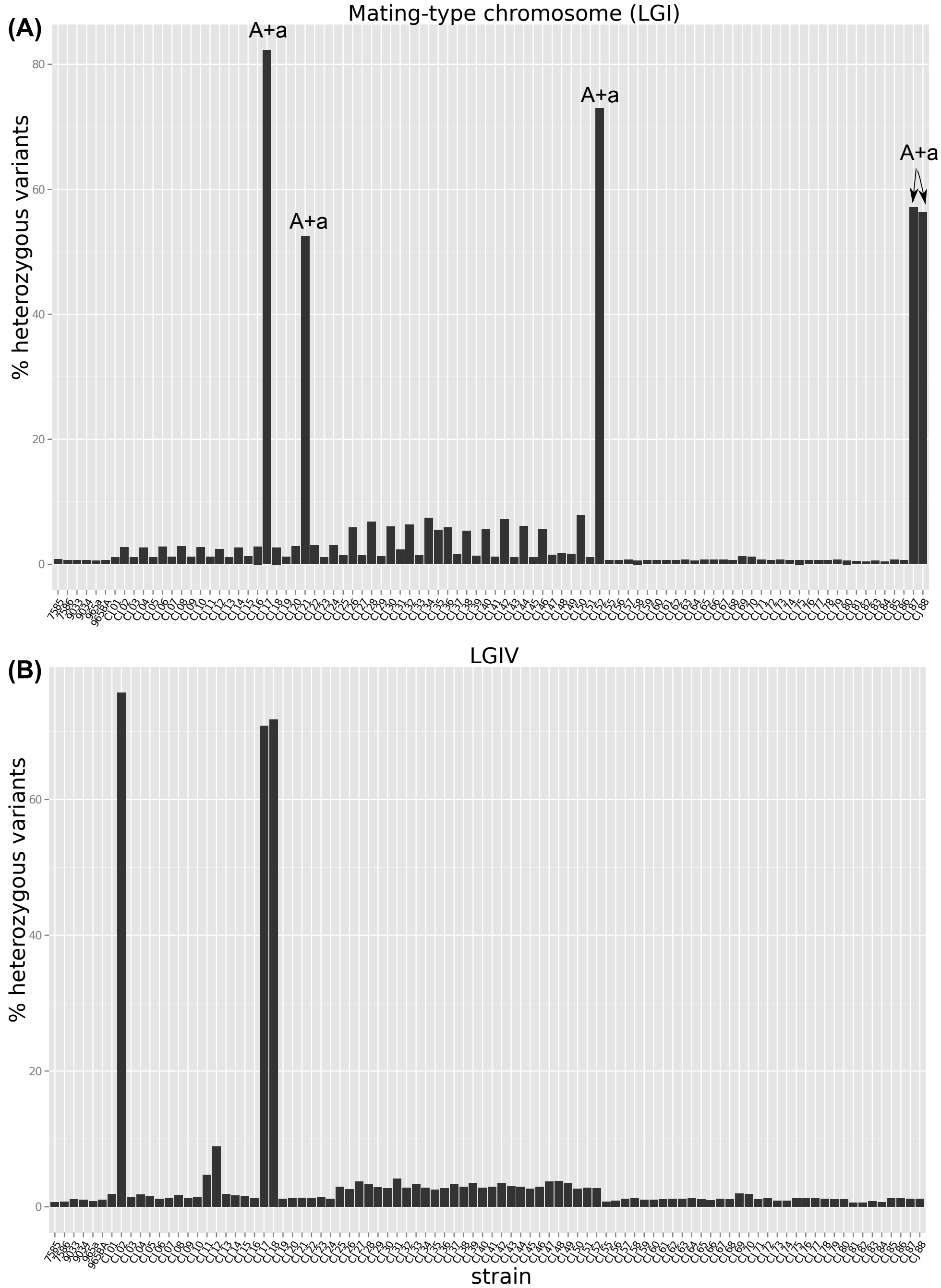

Supplementary Figure 2. The percentage of heterozygous variant called for each *N. tetrasperma* strain during genotype calling in diploid mode with GATK UnifiedGenotyper on the mating-type chromosome (A) and chromosome 4 (B). The 'A+a' above the bars indicates strains that were shown to be heterokaryotic for mating type by blasting the mating-type idiomorph sequences against the deNovo assemblies of the *N. tetrasperma* genomes (see materials and methods for details). The arrows indicate that both CJ87 and CJ88 are heterokaryotic.

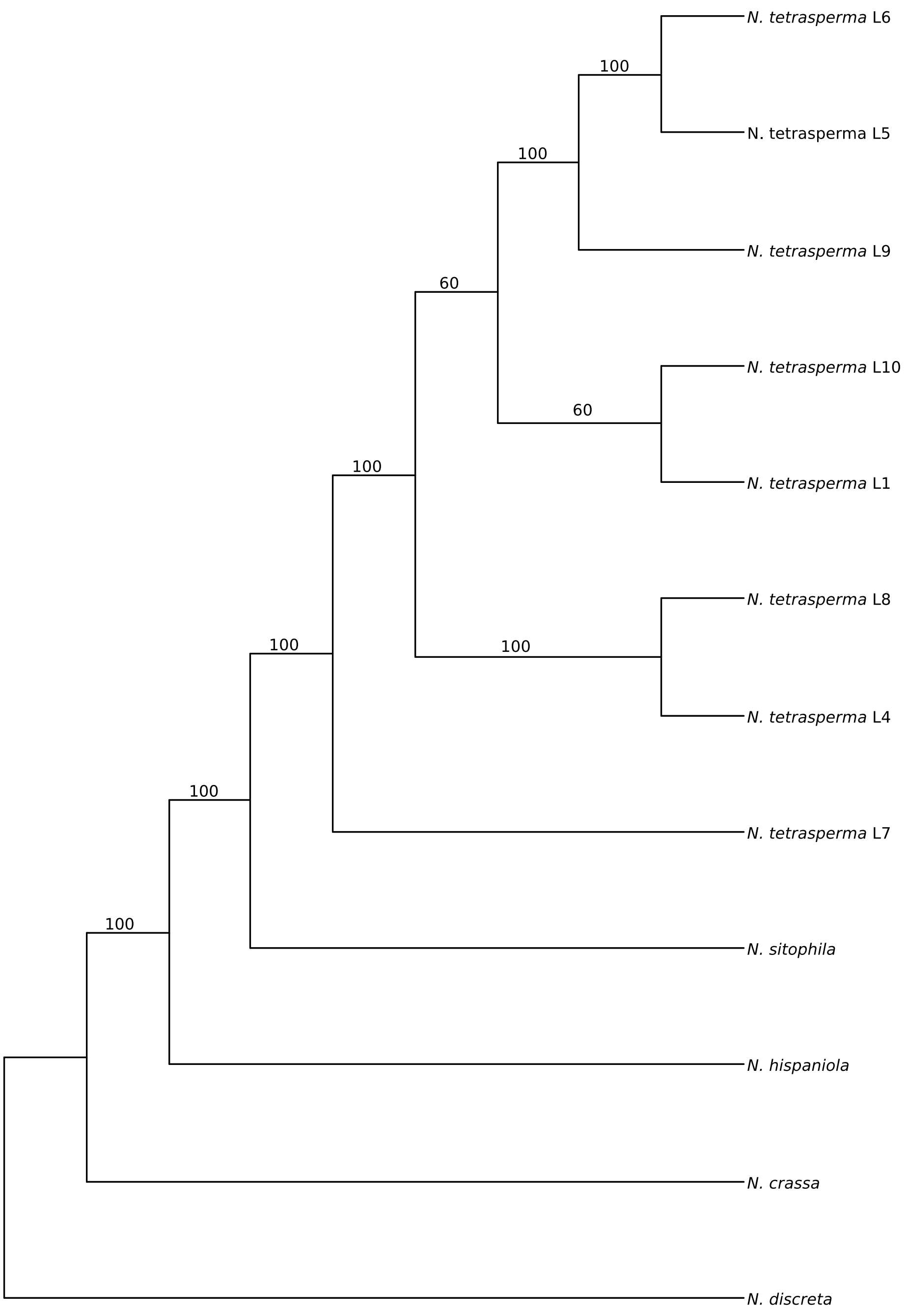

Supplementary Figure 3. The phylogenetic relationship of *Neurospora* species and *N. tetrasperma* lineages inferred using the species tree inference program STAR on 5,292 orthologs. Numbers on the branches indicate the bootstrap support for that relationship expressed as a percentage.

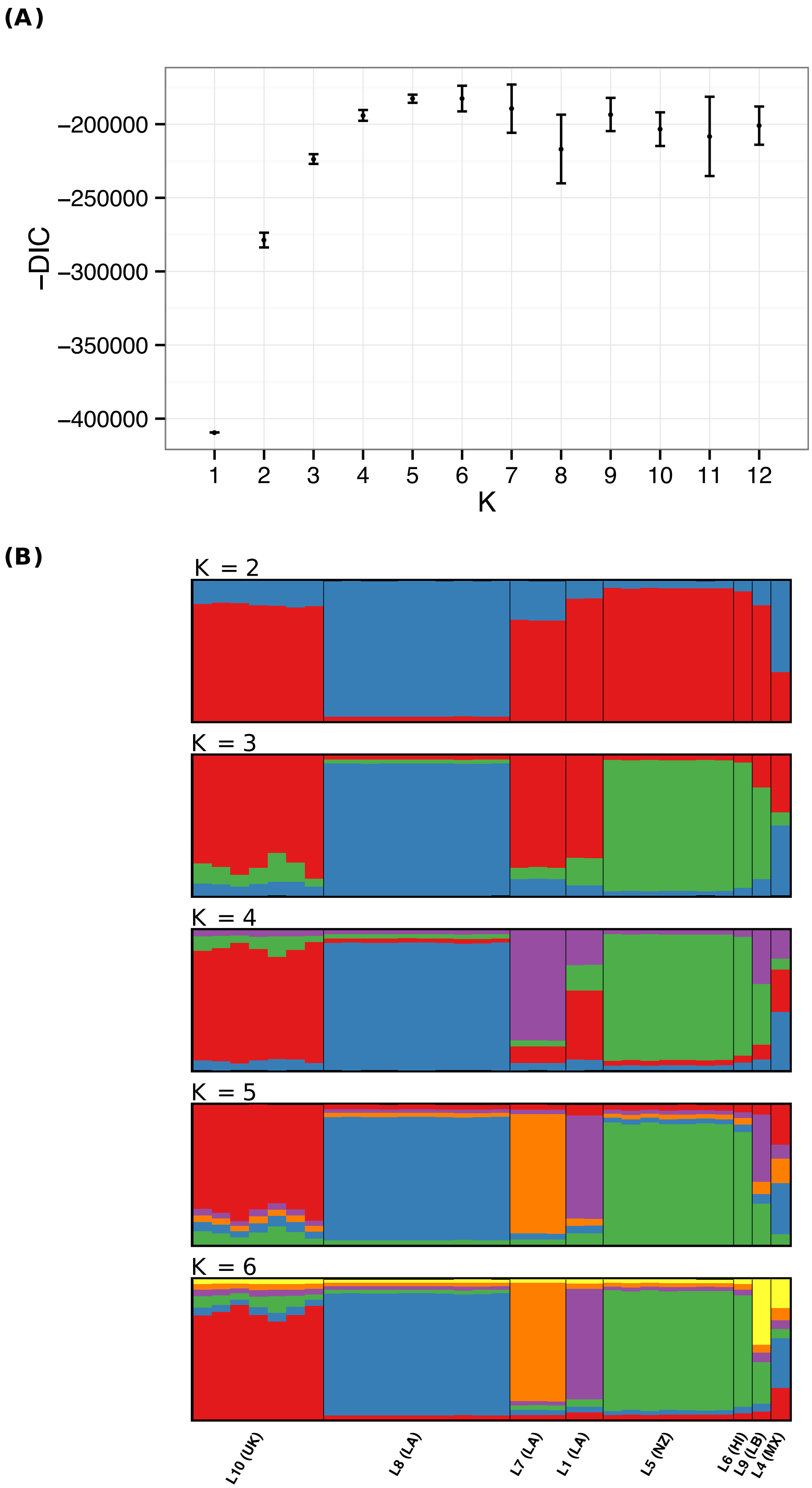

Supplementary Figure 4 Population structure analysis with InStruct (A) The mean deviance information criterion (DIC) (+/- sd) for each K in the InStruct analysis. (B) Population structure inferred using InStruct. Shown are the results from K=2 to K=6. Vertical black bars separate lineages that were identified phylogenetically (see Fig 1A)

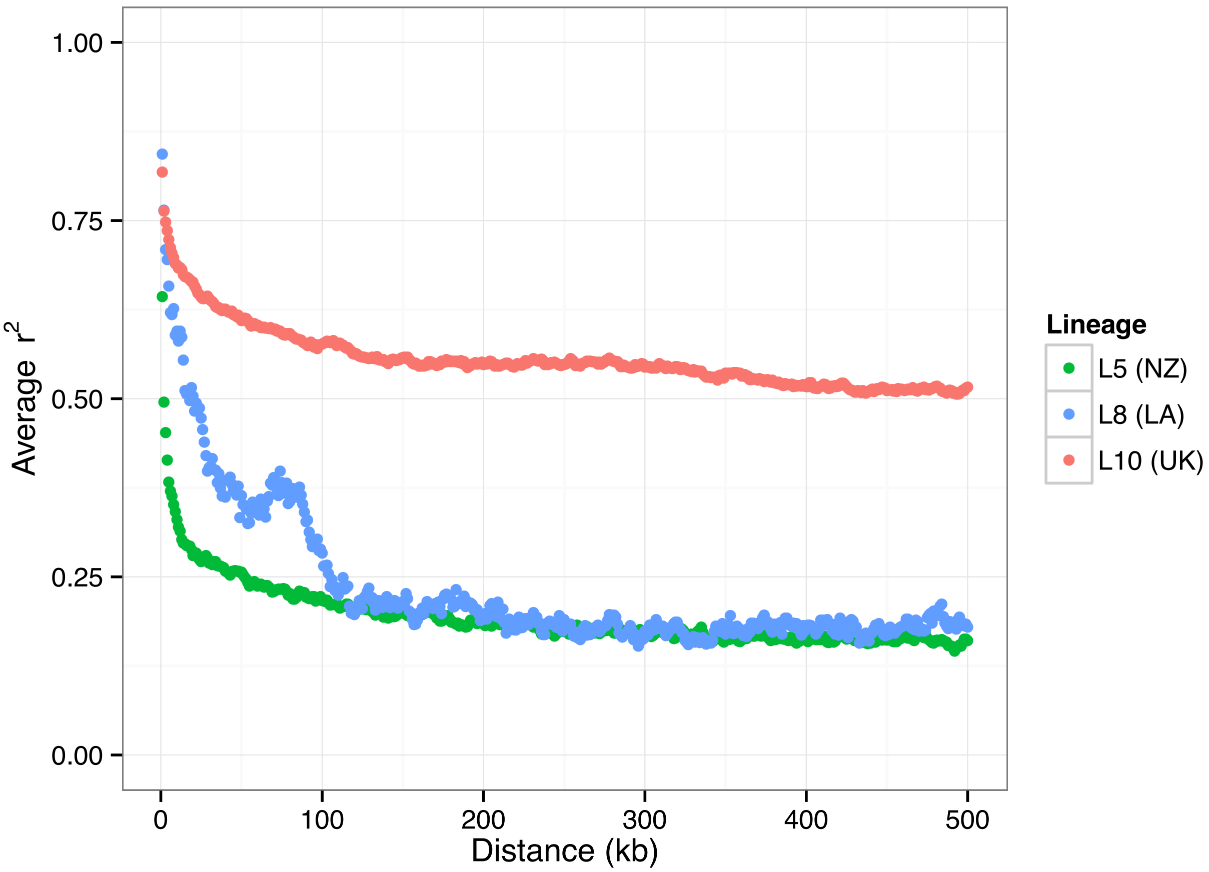

Supplementary Figure 5. Decay of linkage disequilibrium (LD) in *N. tetrasperma* across the autosomes. Plotted is the decay of LD, calculated as r^2, between pairs of autosomal SNPs as a function of physical distance. Each point represents the mean r^2 for snps within a particular distance, increasing by 1kb increments.

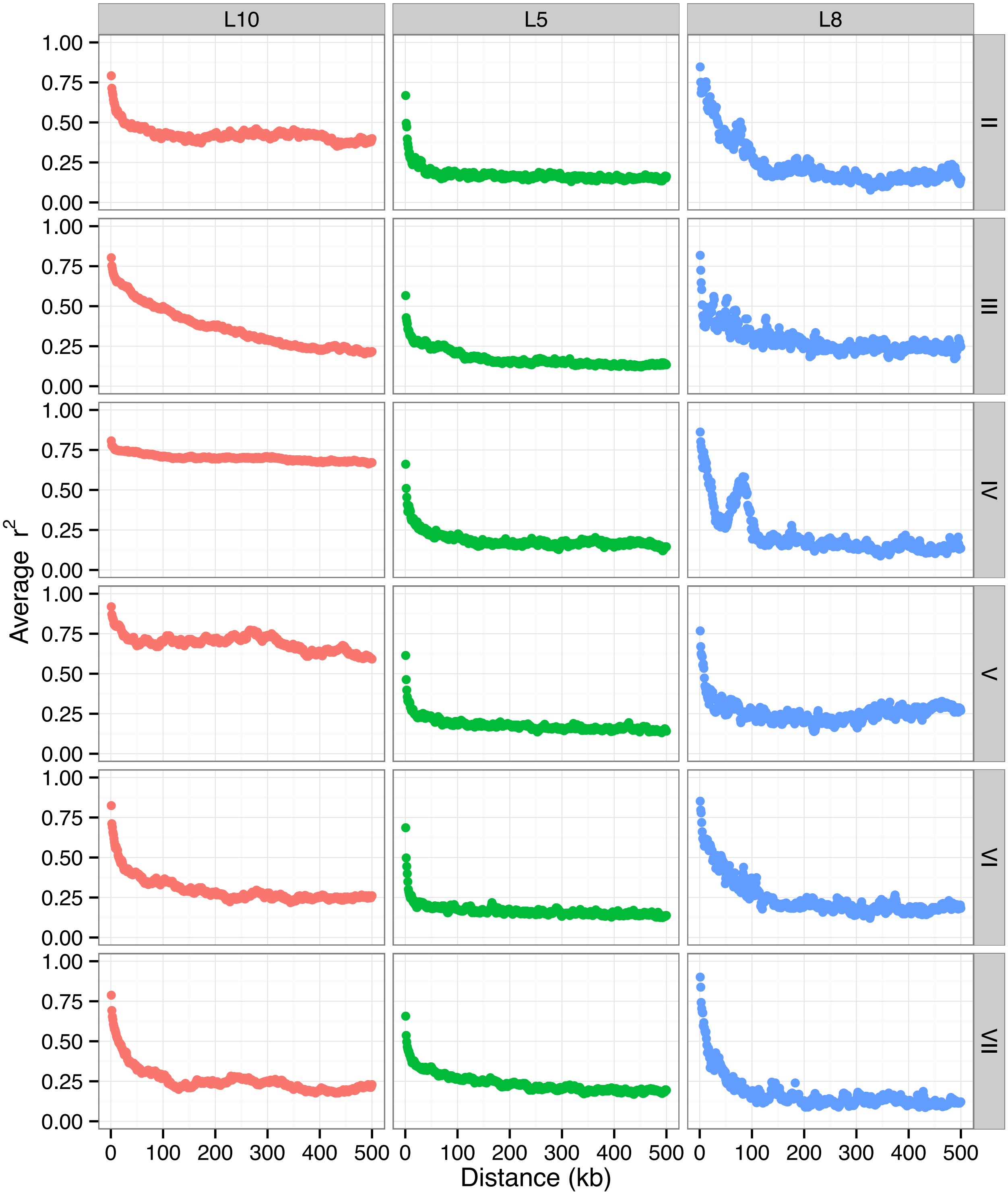

Supplementary Figure 6. Decay of linkage disequilibrium (LD) in *N. tetrasperma* for each of the six autosomes. Plotted is the decay of LD, calculated as r^2, between pairs of autosomal SNPs as a function of physical distance. Each point represents the mean r^2 for snps within a particular distance, increasing by 1kb increments.

L5

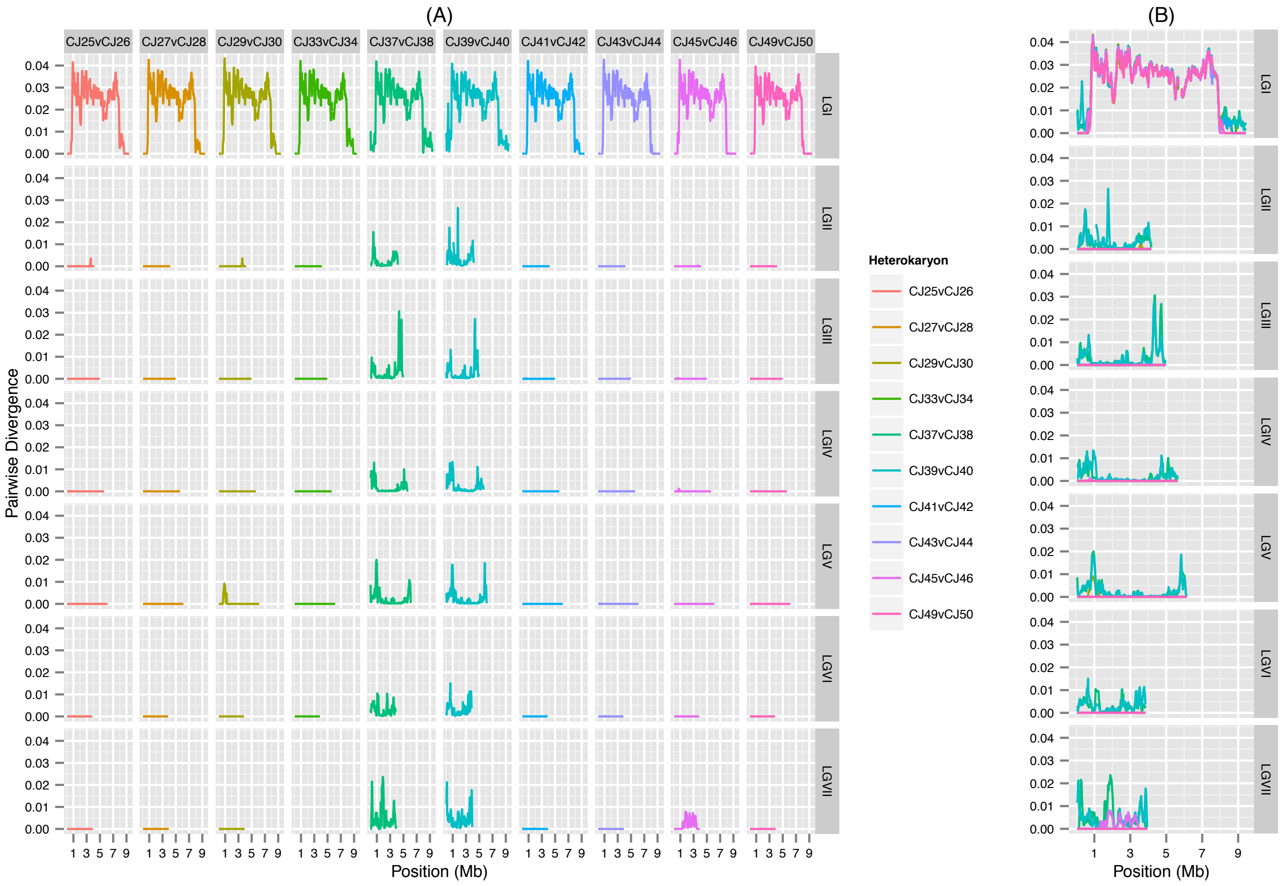

Supplementary Figure 7

L7

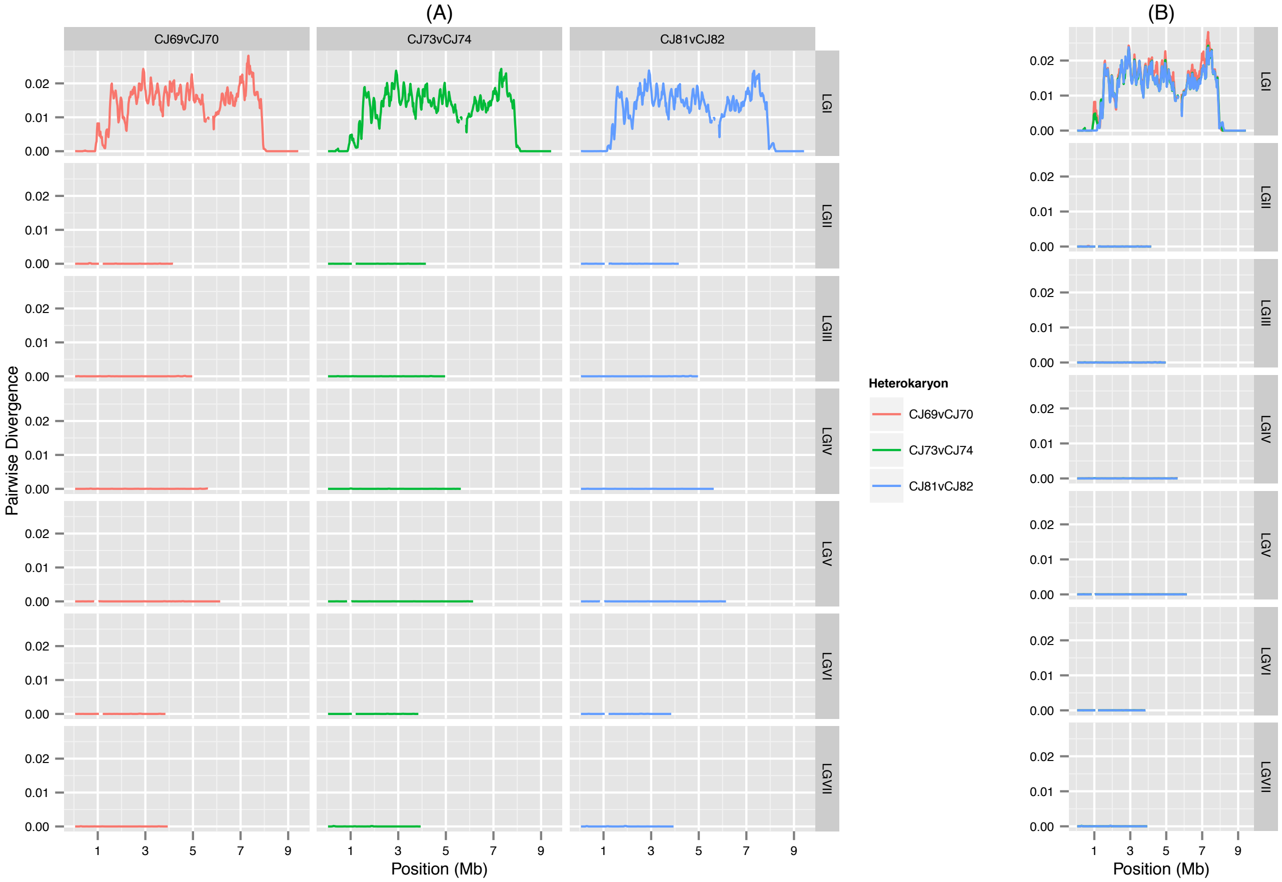

Supplementary Figure 7 continued

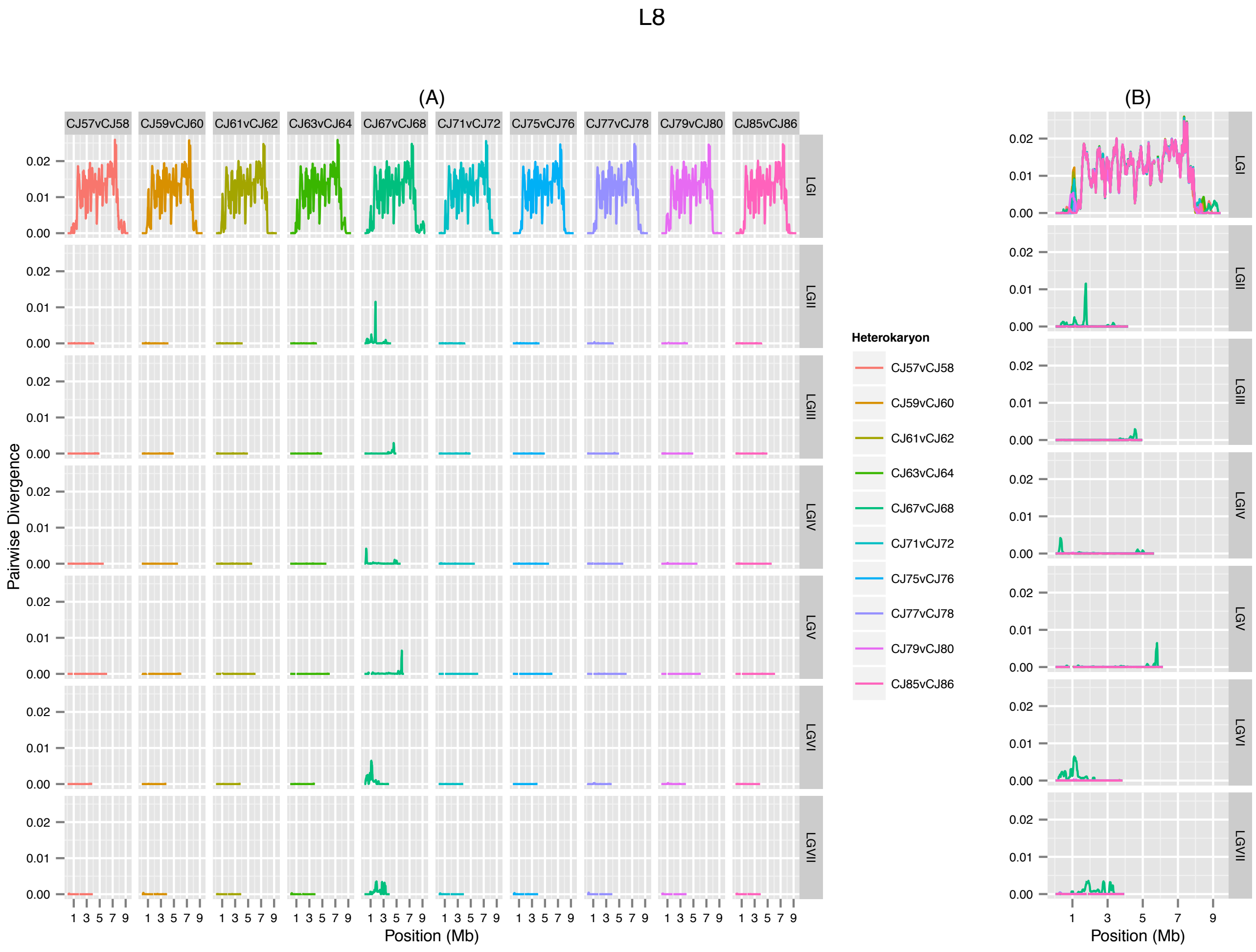

Supplementary Figure 7 continued

L10

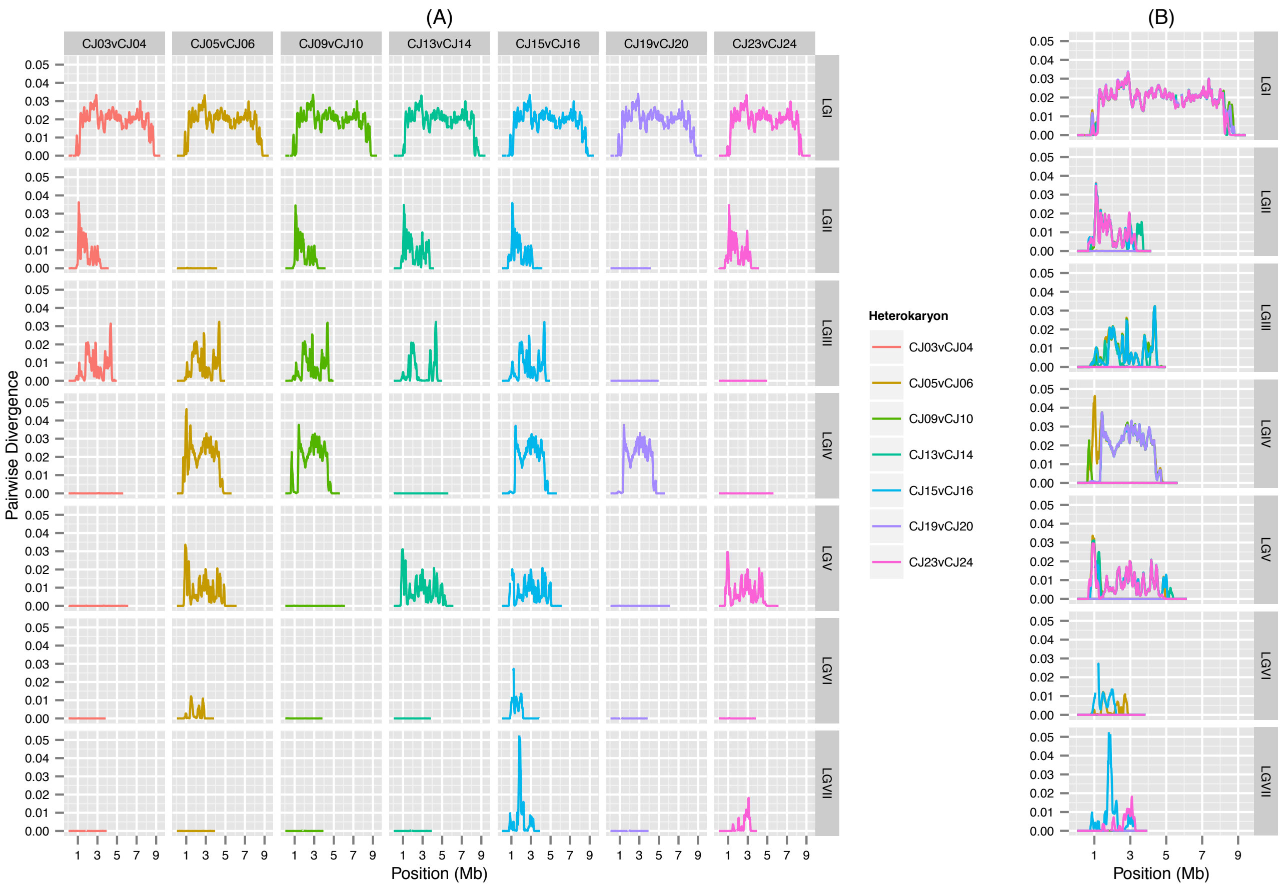

Supplementary Figure 7. Pair-wise genetic distance between homokaryons sampled from the same heterokaryon for the mating-type (*mat*) chromosomes (LGI) and six autosomes (LGII-VII) of *N. tetrasperma* lineages L5, L7, L8 and L10. (A) Individual heterokaryon comparisons. (B) Each heterokaryon comparison overlaid upon each other. The pair-wise genetic distances were calculated as the number of nucletide differences per bp between the sequences, using a 100kb sliding window (step size 20kb).

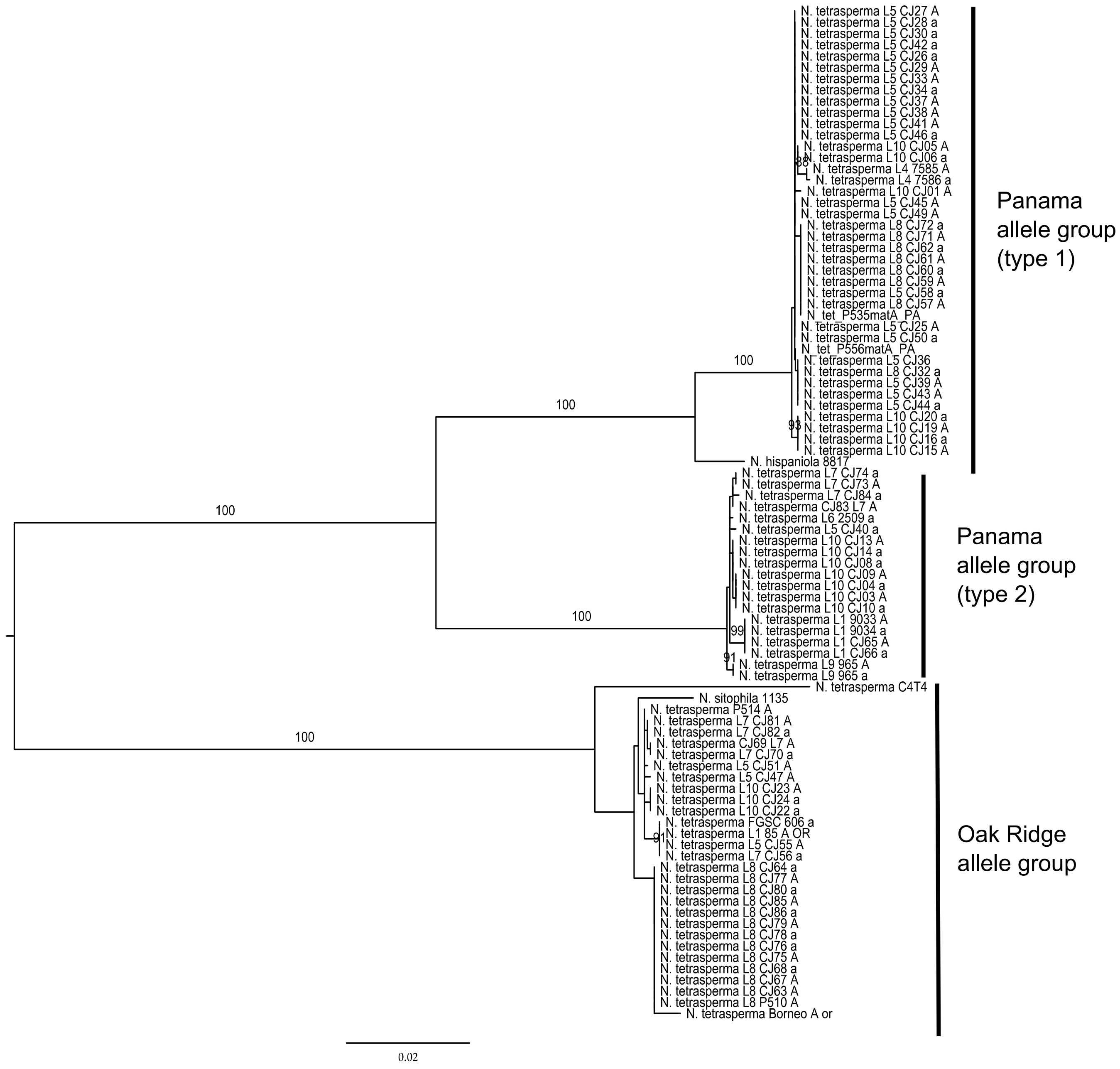

Supplementary Figure 8. Phylogeny of *het-c* alleles with N. tetrasperma strains from this study and heterothallic strains from Hall *et al.* (2010). Numbers on the branches are bootstrap support values expressed as a percentage; values below 70 are not shown.

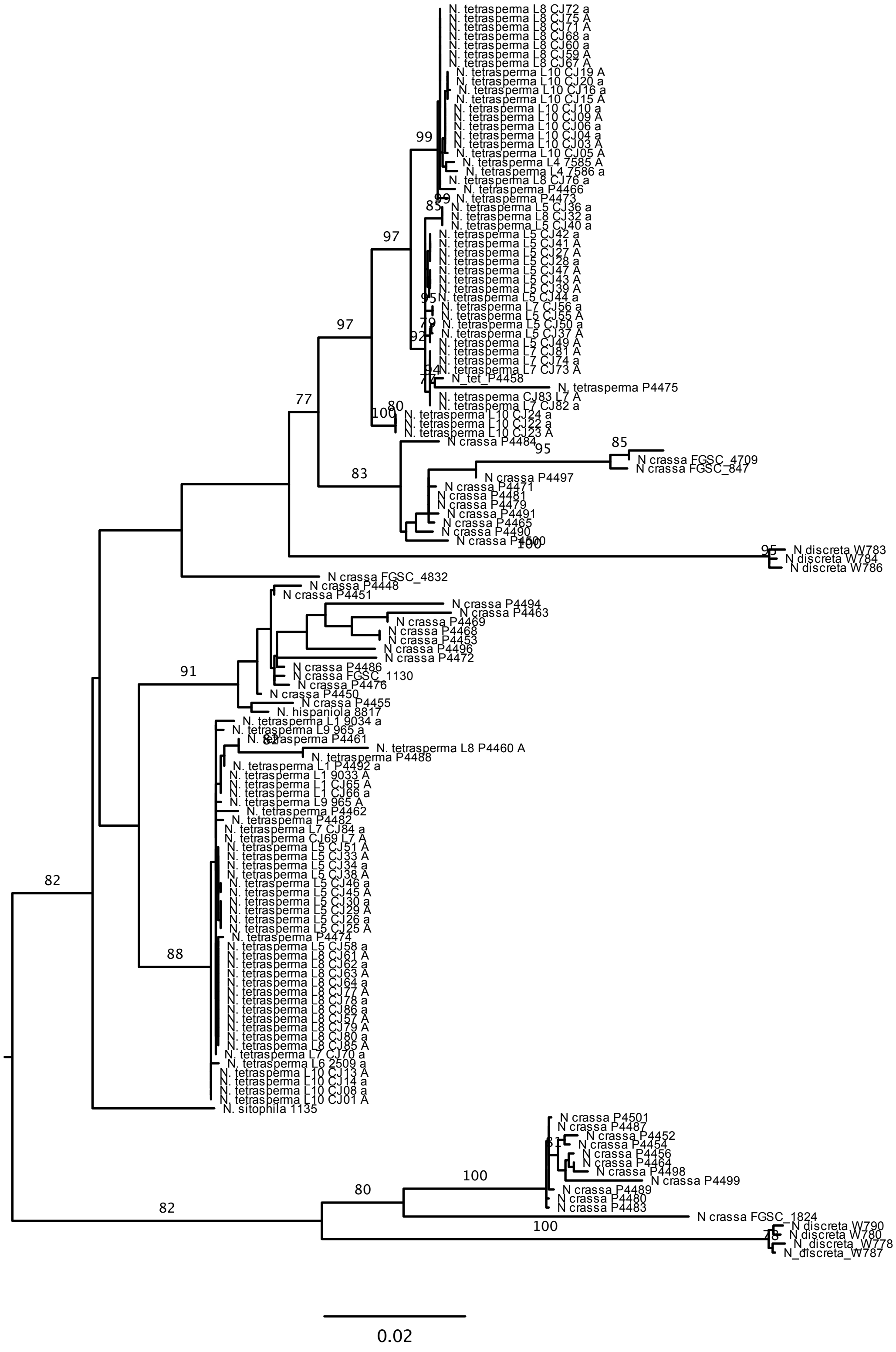

Supplementary Figure 9. Phylogeny of *het-6* alleles with *N.tetrasperma* strains from this study and strains from Powell *et al.*(2007). Numbers on the branches are bootstrap support values expressedas a percentage, values below 70 are not shown.

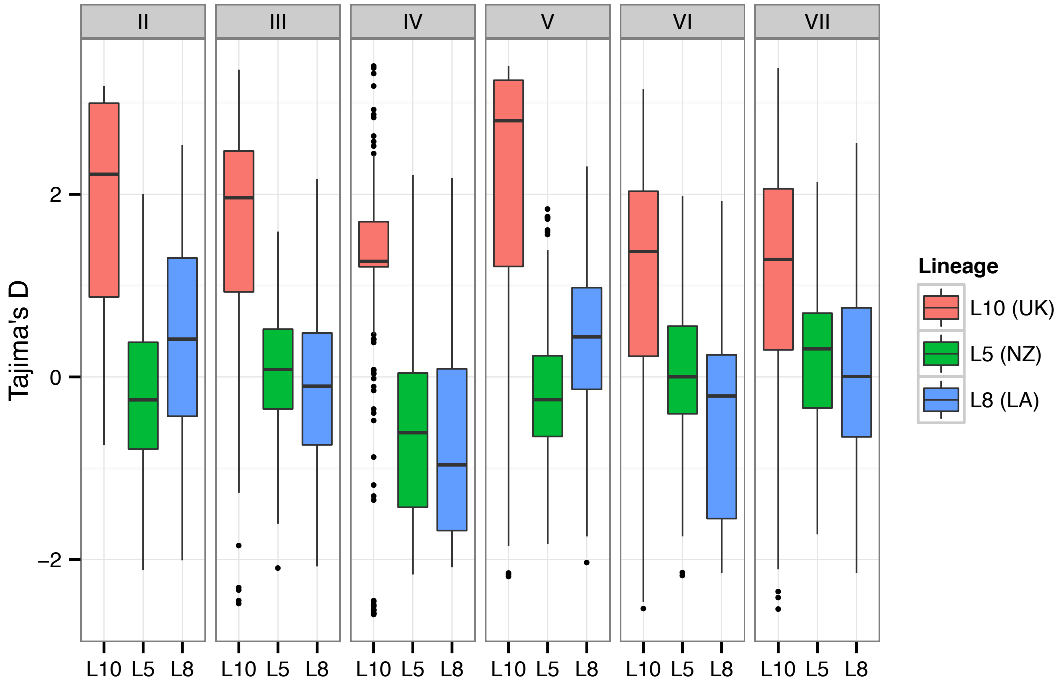

Supplementary Figure 10. Boxplots showing the Tajima's D statistic on the autosomes (II-VII) of *N. tetrasperma* L10 (UK), L5(NZ) and L8 (LA). Negative values indicate an excess of rare variants and a positive value an excess of intermediate frequency variants. Tajima's D was calculated in 25kb non-overlapping windows.

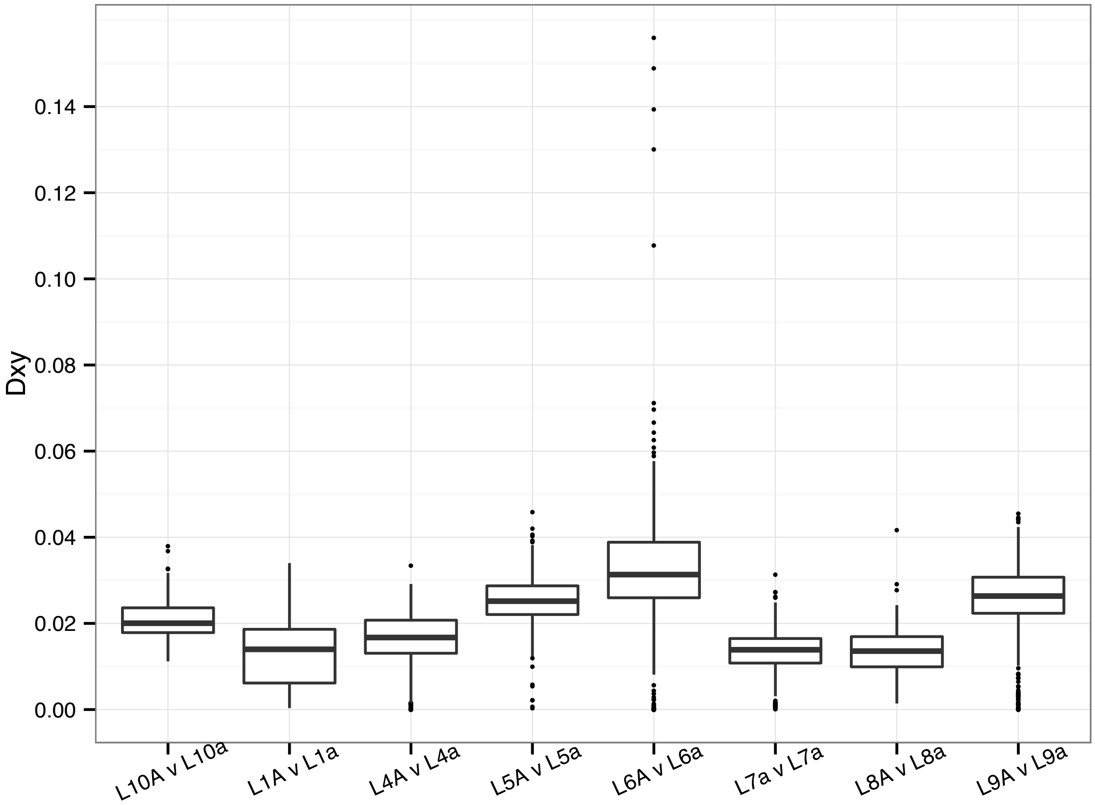

Supplementary Figure 11. The divergence (D_{xy}) between *mat A* and *mat a* mating-type chromosomes in the region of suppressed recombination within each lineage of *N. tetrasperma.* Shown is a boxplot constructed from D_{xy}, the average number of pairwise difference between *mat A* and *mat a* chromosomes from the same lineage. D_{xy} was calculated in 25kb non-overlapping windows in the SR region of each lineage.

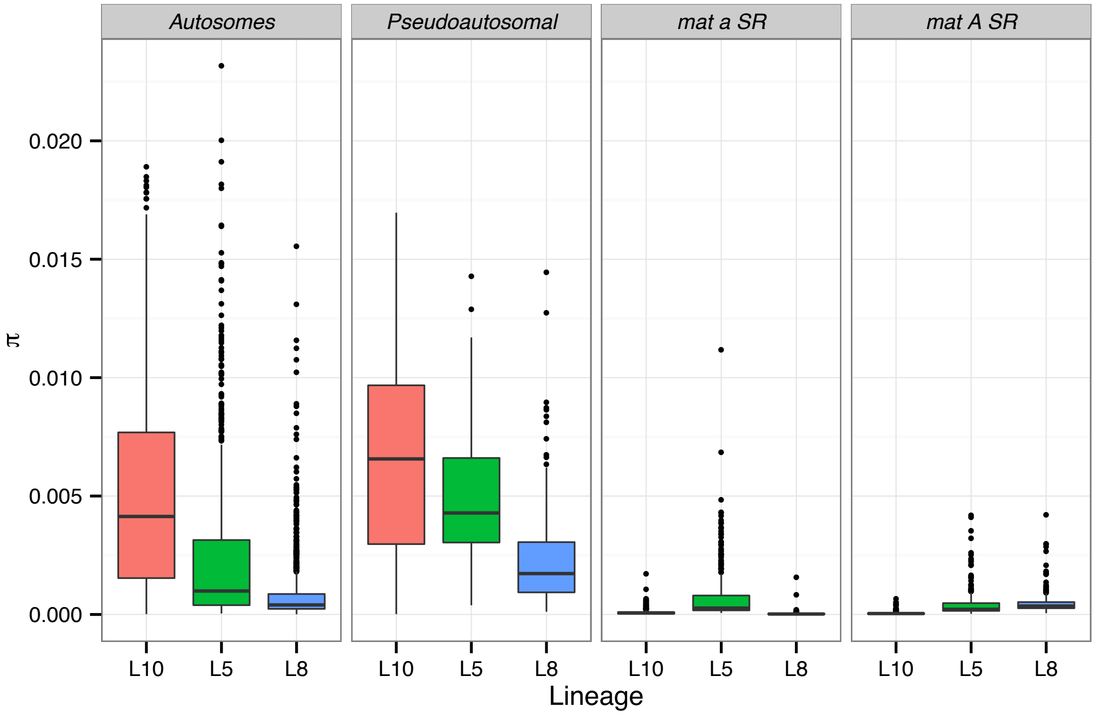

Supplementary Figure 12. Reduced variation on the mating-type chromosomes of *N. tetrasperma*. Boxplots showing the nucleotide diversity (π) on the autosomes, pseudoautosomal regions and regions of suppressed recombination on the *mat* chromosomes, *mat A* (*mat A* SR) and *mat a* (*mat a* SR).

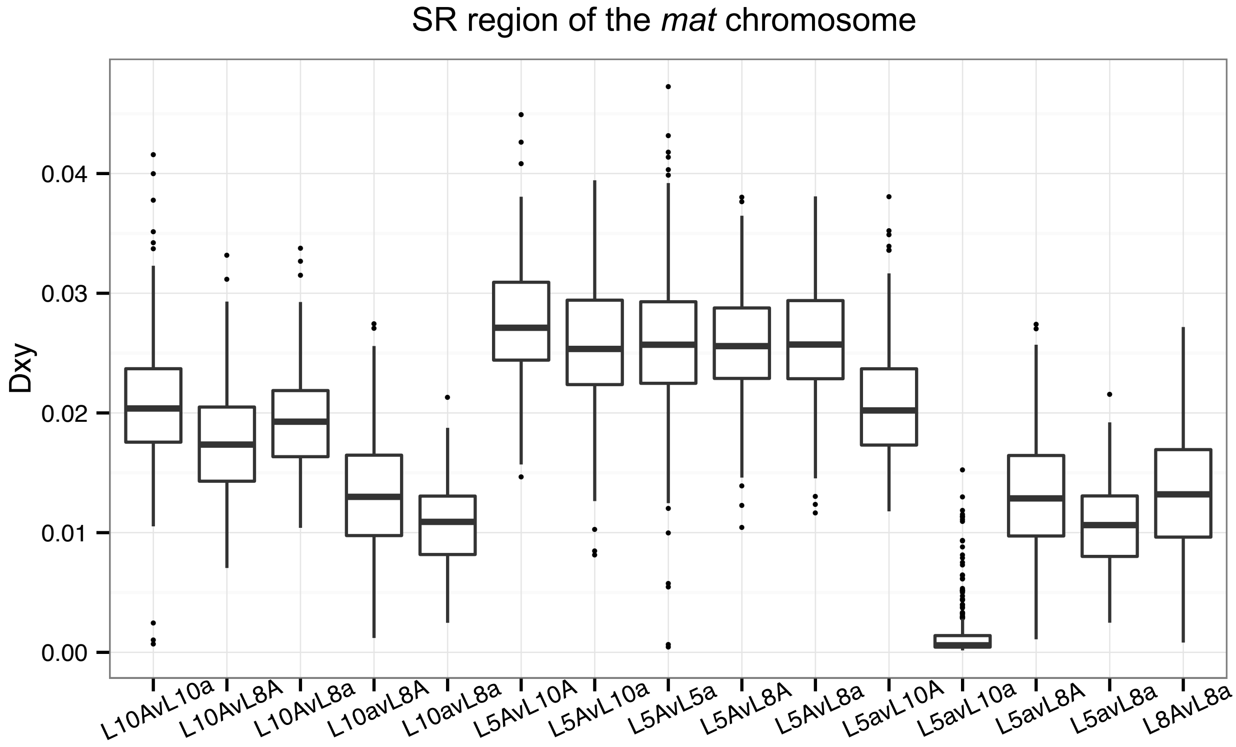

Supplementary Figure 13. The divergence (D_{xy}) between *mat A* and *mat a* mating-type chromosomes in the region of suppressed recombination (SR) within and between lineages L5 (NZ), L8 (LA) and L10 (UK) of *N. tetrasperma*. Shown is a boxplot constructed from D_{xy}, the average number of pairwise difference between SR regions within (e.g., L10 *A* v L10 *a*) or between lineages (e.g., L10 *a* v L8 *a*). D_{xy} was calculated in 25kb non-overlapping sliding windows in the SR region of each lineage.

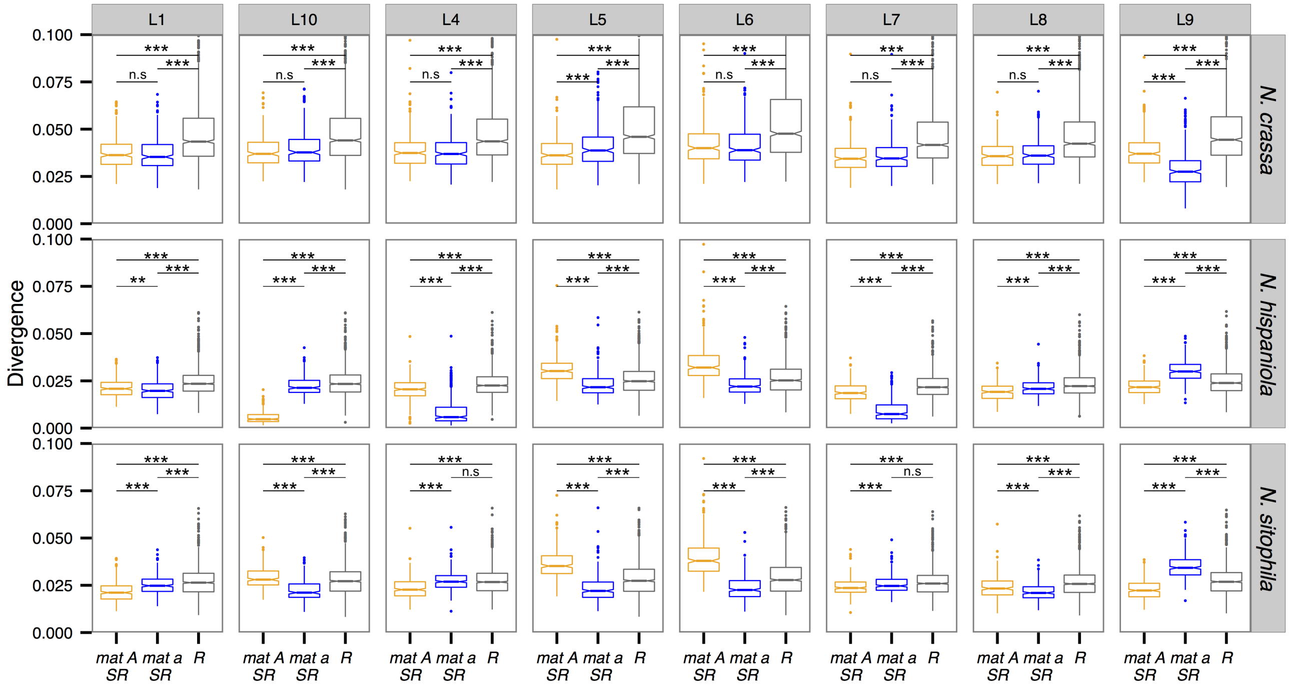

Supplementary Figure 14. Boxplots of mean divergence to heterothallic species in the SR and R regions for all lineages of *N. tetrasperma*. Asterisks above the horizontal black lines are the p-values for the Mann-Whitney U test between each region within a lineage (**:p < 0.01; ***:p < 0.001; n.s :non-significant).

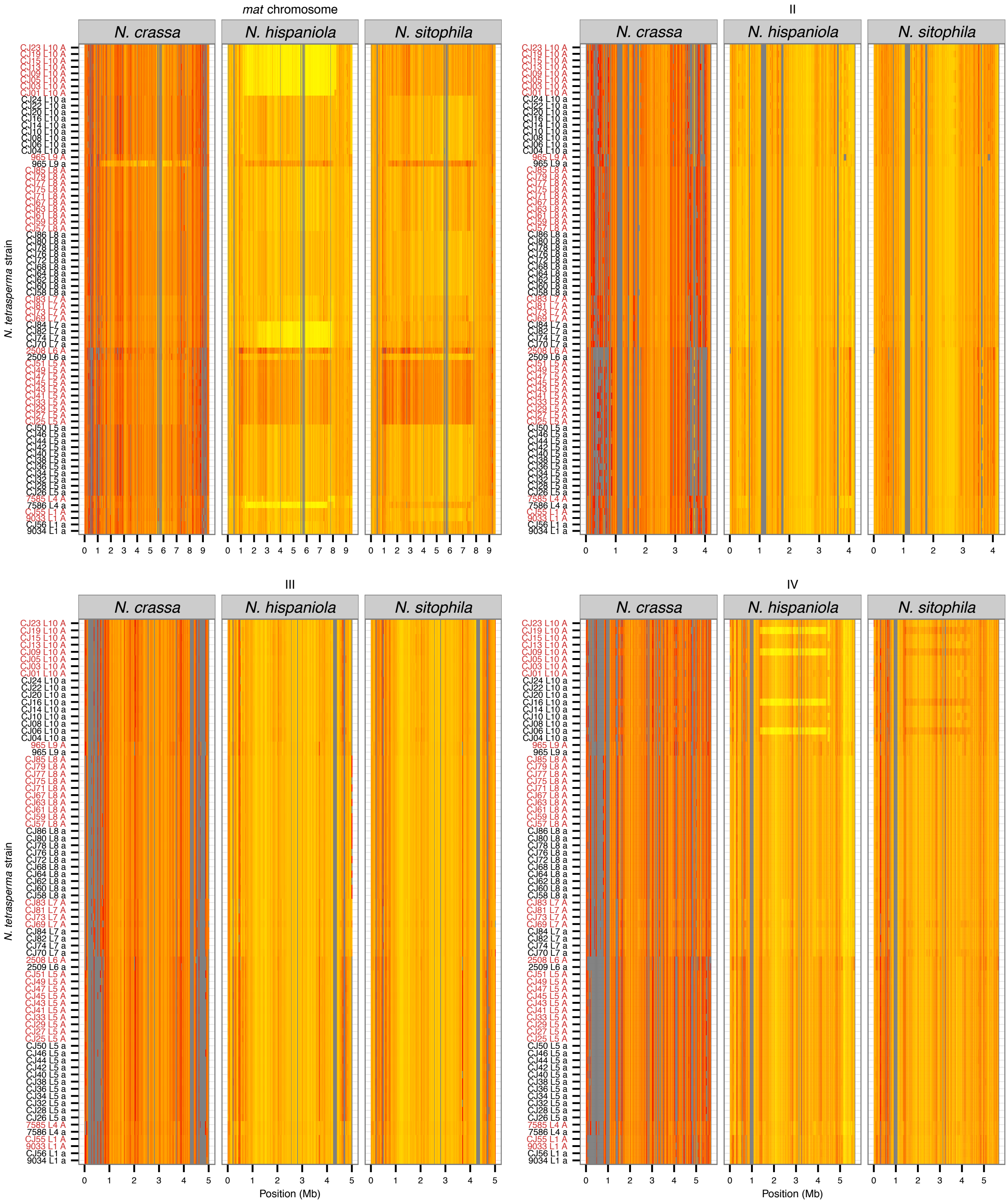

Supplementary Figure 15

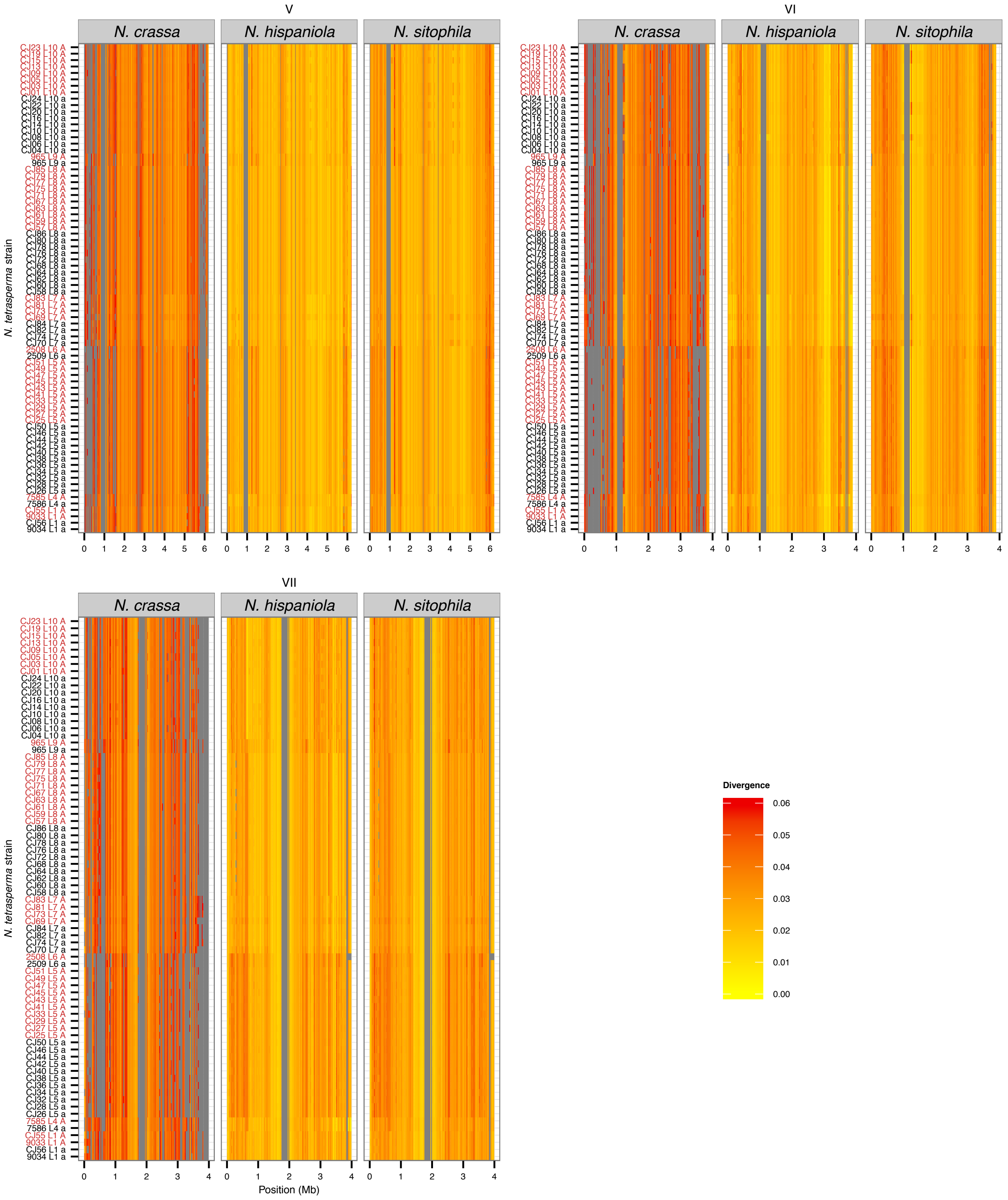

Supplementary Figure 15. Pair-wise sequence divergences between each of the *N. tetrasperma* strains and the heterothallic species: *N.crassa, N. hispaniola* and *N. sitophila*. Each row shows the divergence in 25kb non-overlapping windows across a chromosome between a strain of *N.tetrasperma* and the heterothallic species, indicated in the heading of the column . Strains are sorted by lineage and coloured according to mating-type.

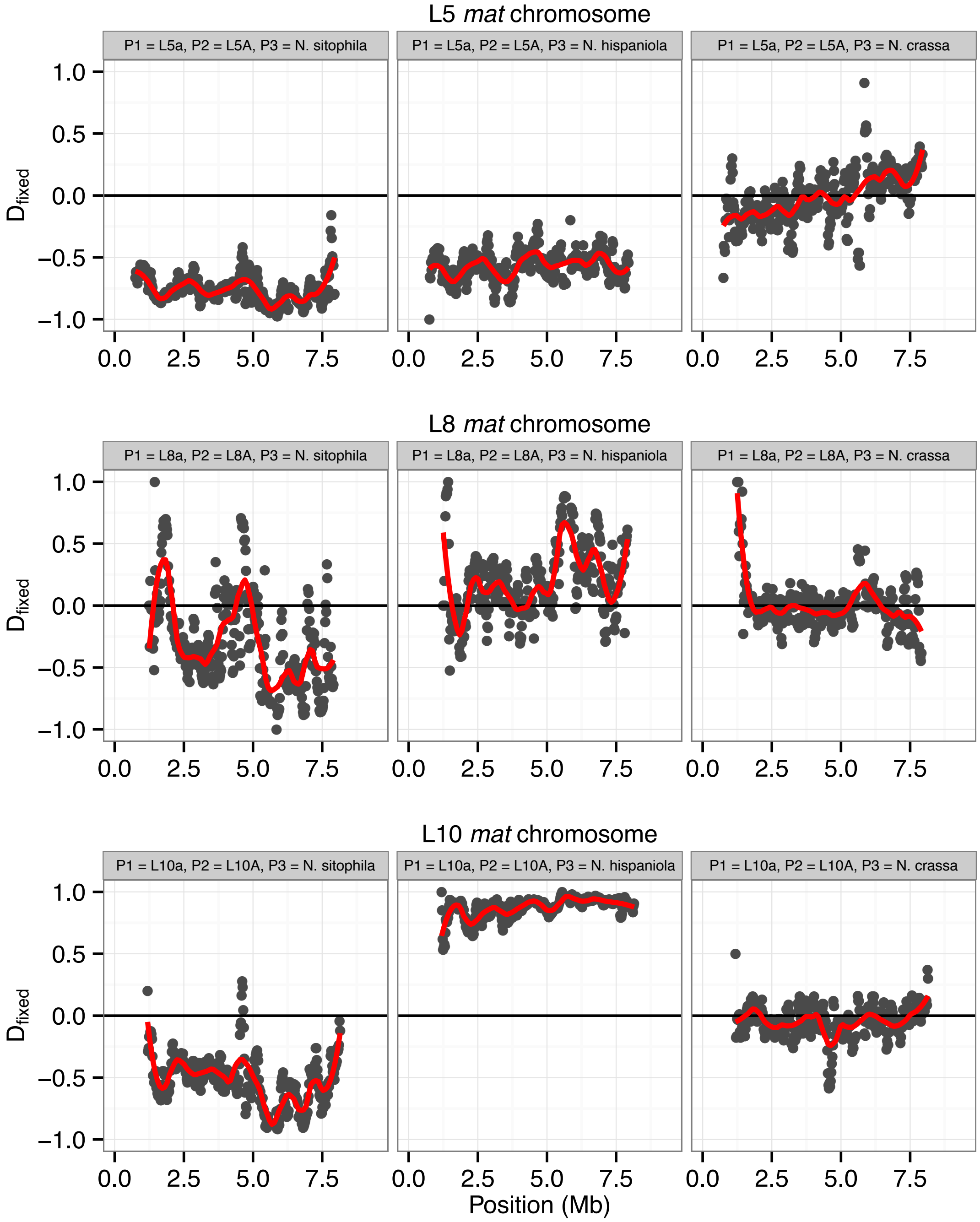

Supplementary Figure 16. Plot of Patterson's *D* statistic across the *mat* chromosomes of *N. tetrasperma* L5, L8 and L10. The P1, P2 were set to the *mat a* and *mat A* from the same lineage respectively, and P3 was one of either *N. sitophila*, *N. hispaniola* or *N. crassa*. *N. discreta* was used in all cases to determine the ancestral state. The calculation of D_{fixed} considered only biallelic sites that showed a fixed difference between *mat A* and *mat a* within a lineage (e.g., a site where all L10 *mat A* chrommosomes carry the derived allele and all L10 *mat a* carry the ancestral allele or vice versa). D_{fixed} was calculated across the chromosome in 100kb windows with a 20kb step. The red line is a smooth line plotted using stat_smooth in the ggplot2 R package using the loess method with a span of 0.2.

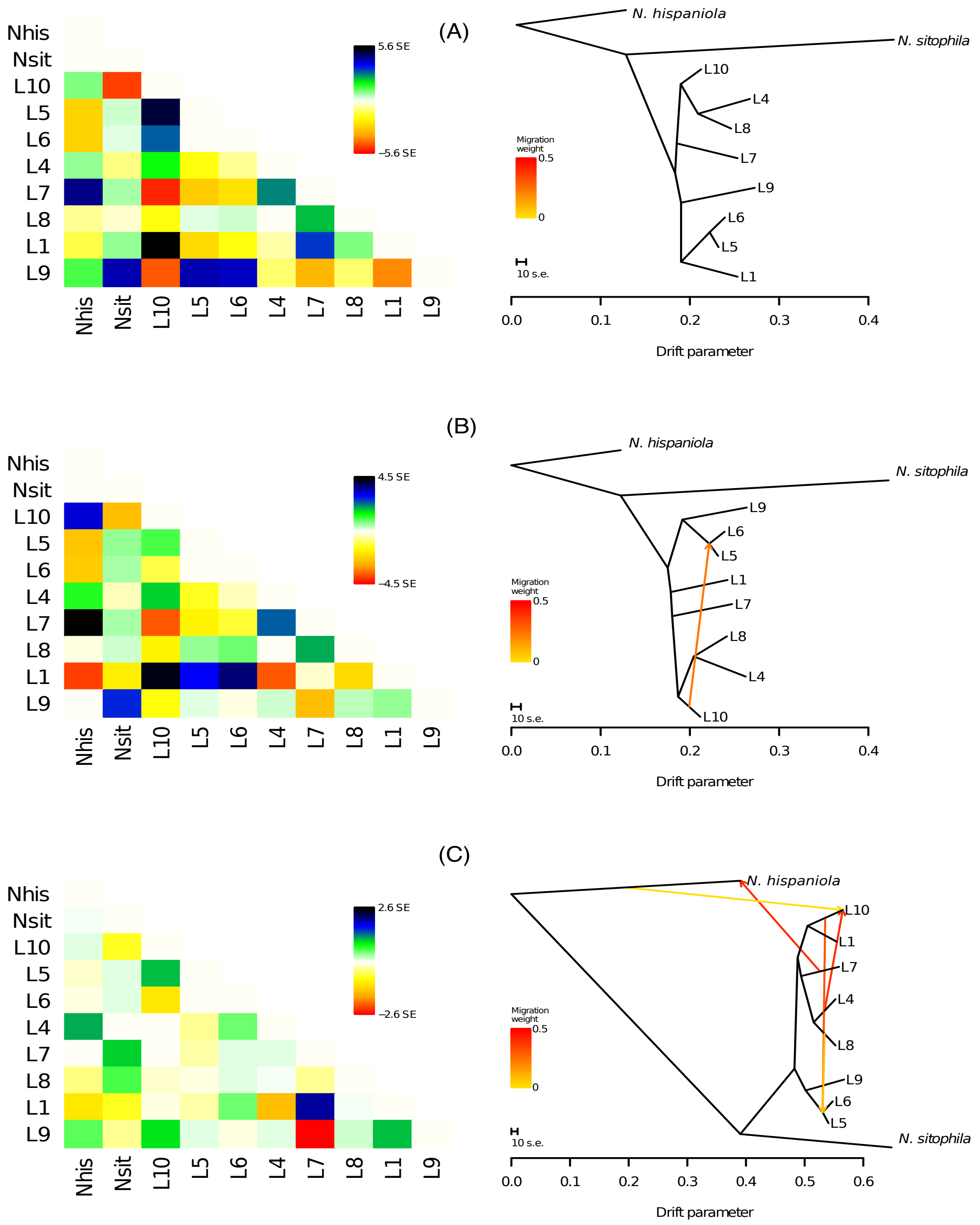

Supplementary Figure 17. Reconstruction of *N. tetrasperma* lineage history using the program *TreeMix*. Plotted is the structure of the tree/graph inferred by *TreeMix* for *N. tetrasperma* lineage, allowing zero (top), one (middle) and five (bottom) migration events. To the left of each tree/graph is the residual fit to the data. Arrows indicate past migration events between populations and are colored according to their weight. The scale bar shows ten times the average standard error of the entries in the sample covariance matrix.

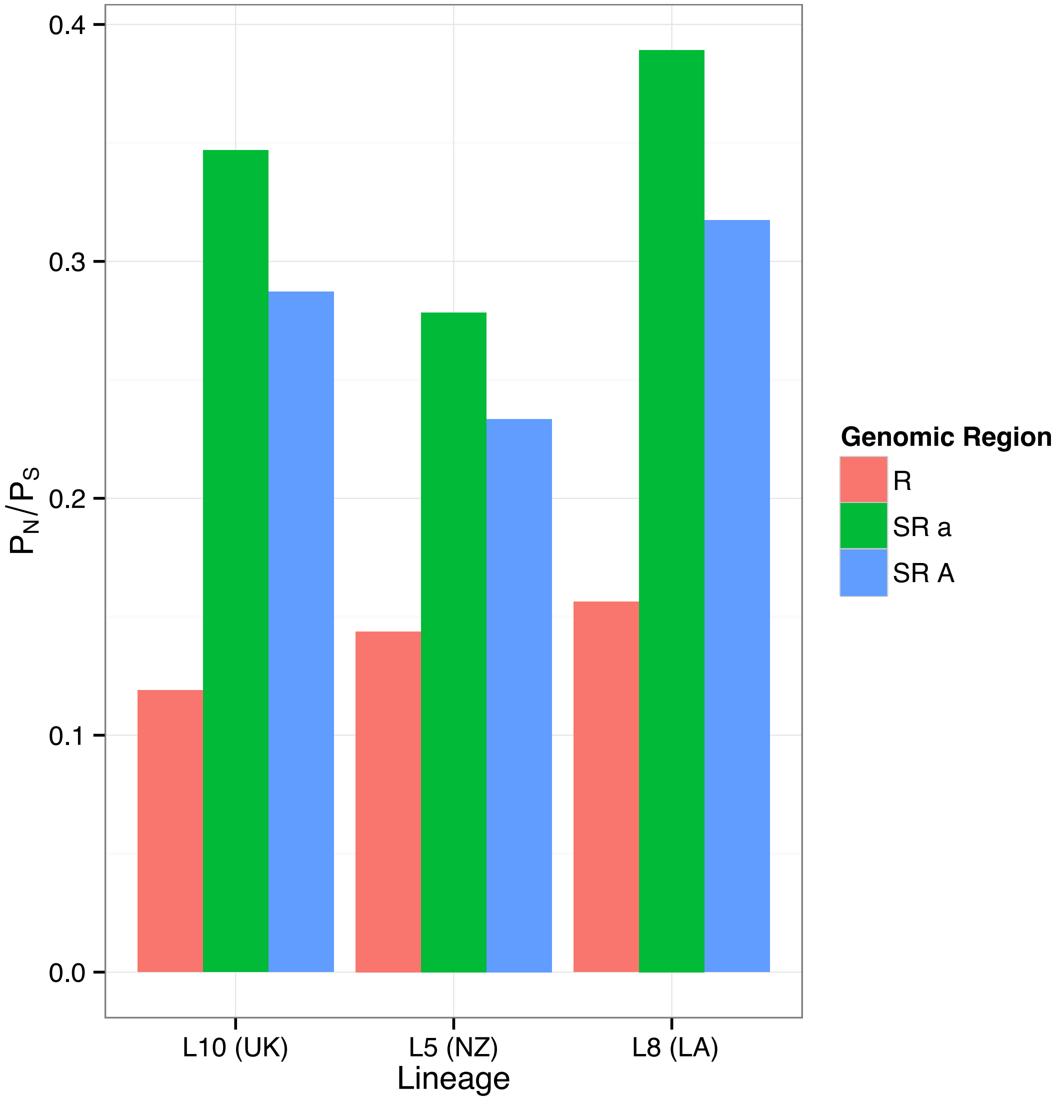

Supplementary Figure 18. The ratio non-synonymous polymorphisms per non-synonymous sites P_N to synonymous polymorphisms per synonymous sites P_S for lineages L5, L8 and L10. R, recombining regions; *mat* SR A and mat SR a ,regions of suppressed recomnination on *mat* A and a chromosomes respectively.

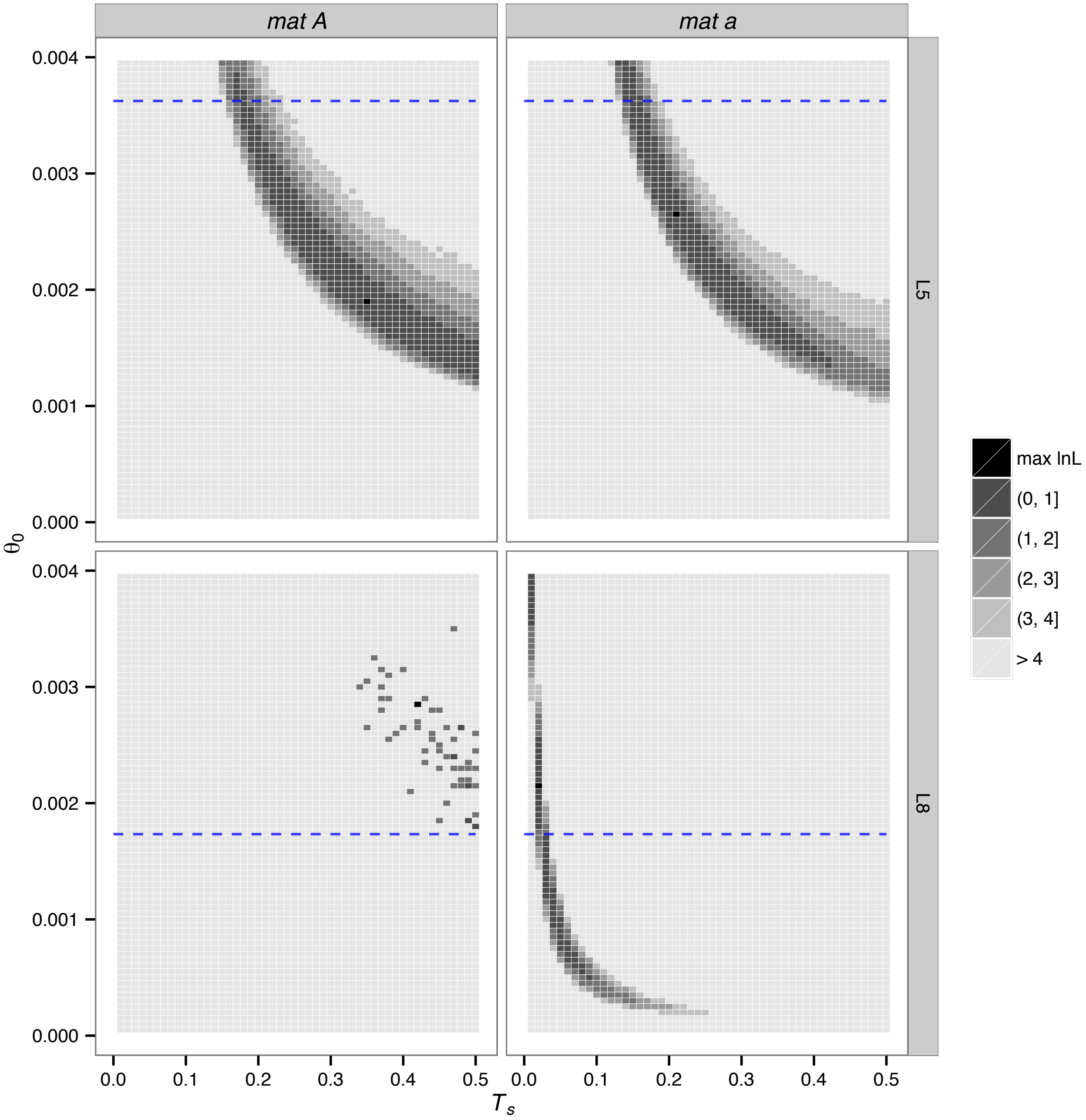

Supplementary Figure 19

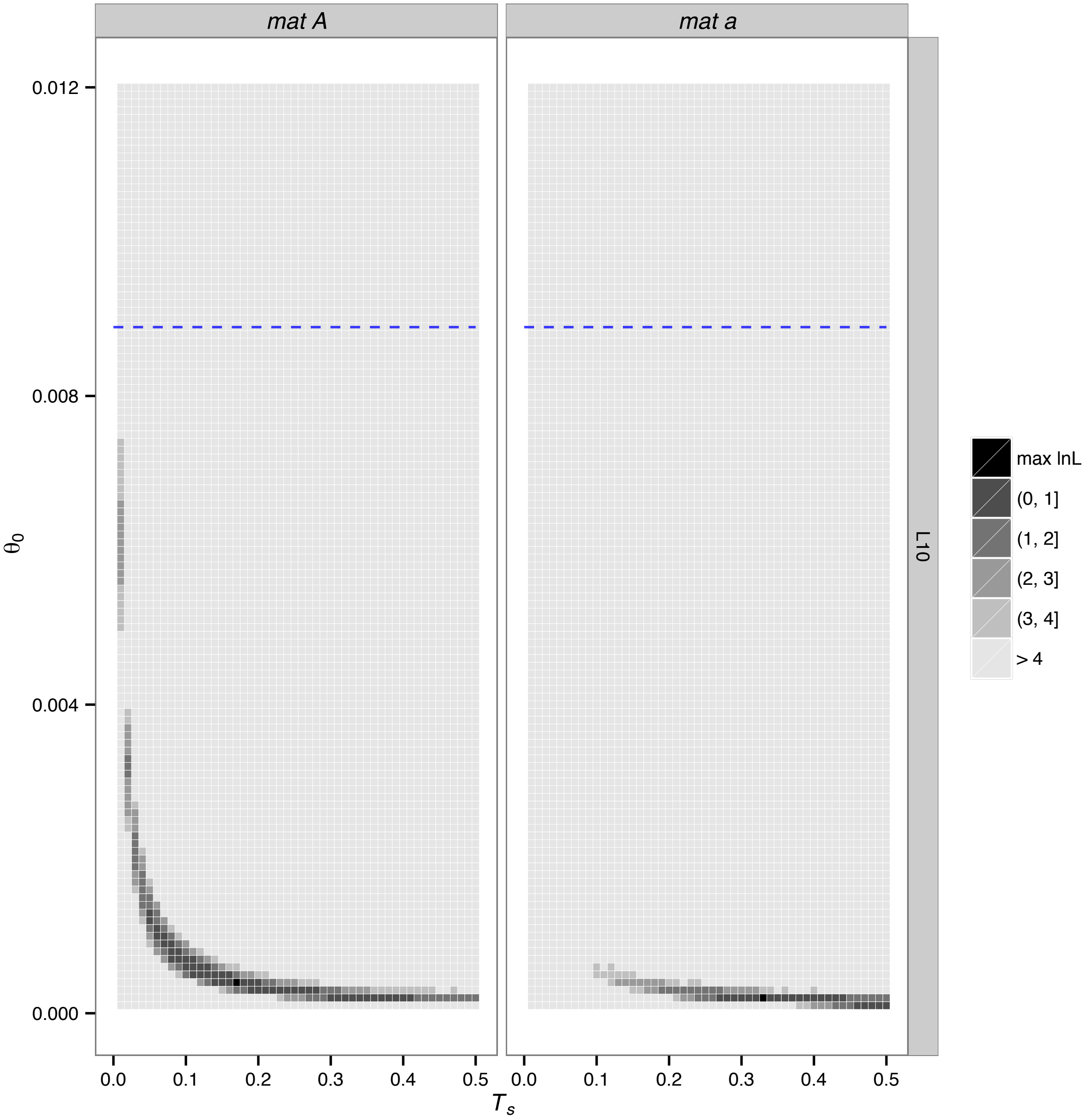

Supplementary Figure 19. Fit of a selective sweep model to the neutral polymorphisms within the regions of suppressed recombination of L5, L8 and L10. The maximum likelihood value is shown in black, and the shading of each grid represents the magnitude of the reduction in log likelihood for a given combination of θ_0 (neutral diversity in the absence of a sweep) and T_s (the time in N_e generations since the sweep occured). The dashed blue line shows the level on neutral diversity on the autosome, estimated as the mean value across genes of π at four-fold degenerate sites.
