# Supplementary material for: Introgression maintains the genetic integrity of the mating-type determining chromosome of the fungus Neurospora tetrasperma
Source: Genome Res. 2016 Apr;26(4):486–98. doi: 10.1101/gr.197244.115 (PMC4817772; doi:10.1101/gr.197244.115)
Supplement: Supplemental Material [file supp_gr.197244.115_Supplemental_Methods.pdf]

Read Mapping to *N. tetrasperma* reference genomes

The quality filtered paired-end reads of *N. tetrasperma* were mapped to both the 2508 (*mat A*) and 2509 (*mat a*) reference genomes of *N. tetrasperma* (Ellison et al., 2011b) using the program BWA 0.5.9 (Li and Durbin, 2010)(parameters -I -n 5 and the remainder of the parameters set to default values). The bam files were processed with samtools 0.1.18 (Li et al., 2009) and PCR duplicates were removed using the samtools rmdup function. Realignment of the bam file around indels was carried out using the IndelRealigner tool from GATK v2.3.9. The 2509 (*mat a*) bam files were chosen for calling the genotype of the *N. tetrasperma* strains, as the majority of the strains had a higher mean depth of reads across the genome in the 2509 bam files than in the 2508 bam files (Supplementary Table 1).

Genotype calling

Genotyping of the *N. tetrasperma* strains was carried out using the GATK UnifiedGenotyper v2.3.9 (DePristo et al., 2011) with the following parameters: --sample_ploidy 1 and --output_mode EMIT_ALL_SITES. Strains from the same lineage had their variant calling carried out together. The resulting vcf files were used to create a consensus sequence for each strain of *N. tetrasperma.* A genotype call at a variant site was included in the consensus sequence of a strain if that site had a QUAL ≥ 60, QD > 2.0, FS < 60, MQ > 40 and the genotype call had a genotype quality (GQ) ≥ 40, otherwise it was called as 'N'. A genotype call had to have a $8 \leq$ depth ≤ 250, otherwise the site was called as 'N'. Sites were masked as 'N' if they that were found within 5bp of an indel. Sites in the consensus sequence that were located in repetitive regions of the genome, as defined in the 2509 reference genome, were also masked as 'N' in the consensus sequences. These consensus sequences were combined to form

alignments for each of the seven chromosomes of *N. tetrasperma*, which were used for all subsequent analyses.

The quality filtered paired end read data for *N. hispaniola* 8817 and *N. sitophila* 1135 (Supplementary Table 1) were also mapped to the 2509 genome using BWA 0.5.9, but using -n 10. The bam files were processed in samtools 0.1.18 following the same procedures as applied to the *N. tetrasperma* alignments, and realignment around indels was also carried out using GATK. Consensus fastq files for *N. hispaniola* and *N. sitophila* were produced using samtools mpileup and bcftools and vcfutils.pl vcf2fq script from the samtools package (parameters -d7 -D250 -Q10). Sites in the consensus FASTQ file with a consensus quality score less than 30 were converted to 'N'. As this is haploid data, all heterozygous sites were masked as 'N' in the consensus sequence of both species.

***De novo* genome assembly**

SOAPdenovo v1.05 (Li et al., 2010) was used for the *de novo* assembly of the 92 *N. tetrasperma* strains (Supplementary Table 1). In order to assess which kmer gave the best assembly quality, we assembled the CJ01 genome with kmers ranging from 17 to 63 and compared the number of scaffolds and N50 values of the assembled genomes. We chose 37 as the best kmer size to assemble all 92 genomes, and kept other parameters as default in SOAPdenovo. The *de novo* assemblies were used in two analyses described below: the confirmation of mating types for each *N. tetrasperma* strain and the extraction of gene sequences for heterokaryon incompatibility (*het*) loci.

Filtering of heterokaryotic strains and clones

We took two different approaches to verify mating-type and homokaryosis of the strains of *N. tetrasperma* sequenced in this study. First, we carried out blastn searches of each *de novo* assembled *N. tetrasperma* genome using the *mat A* and *mat a* idiomorph gene sequences from the *N. tetrasperma* 2508 and 2509 reference genomes, respectively. A genome of a strain that received a significant hit with high sequence identity for only one of the idiomorphs was identified as homokaryotic for one or the other mating type, while significant hits with both idiomorphs was interpreted as mating-type heterokaryosis. Second, we carried out genotype calling in diploid mode with the GATK UnifiedGenotyper v2.3.9. Using this method, strains that are heterokaryotic for mating type were identified by a large number of heterozygous genotype calls along the *mat* chromosome associated with the presence of a large region of suppressed recombination between the *mat A* and *mat a* chromosomes. Both analyses identified strains of *N. tetrasperma* that appear to be mating-type heterokaryons (Supplementary Table 1), with strains that showed a significant hit for both *mat* idiomorphs also showing an excess of heterozygous calls on the mating type chromosomes (Supplementary Fig. 2A). In addition, we observed strains that did not show any indication of being heterokaryotic for mating-type by either of the criteria outlined above, but which did show an excess of heterozygous genotype calls on the autosomes (Supplementary Fig. 2B). These strains were scored as homokaryotic for mating-type while heterokaryotic on other chromosomes. Due to the uncertainty surrounding the causes of this pattern, we excluded these strains from further analyses.

The mating-type blast search also revealed that some pairs of homokaryons isolated from the same heterokaryon were of the same mating type (*i.e.*, CJ39 and

CJ40; CJ47 and CJ48). These were considered clones, and one of the strains from the pair was excluded from all downstream analyses. Finally, strains that were identified as identical to a strain from a different heterokaryon, based on the autosomal SNP phylogenetic analysis presented in Figure 1, were also considered to be clones and were excluded from all further analyses.

Genome alignments of outgroup heterothallic species

The genome sequences of heterothallic species *N. crassa* (FGSC 2489, *mat A*, finished v 10.0, downloaded from the Broad Institute http://www.broadinstitute.org/) and *N. discreta* (FGSC 8579, *mat A*, v 1.0 February, 2009, downloaded from DOE Joint Genome Institute http://genome.jgi-psf.org/) were aligned with *N. tetrasperma* reference genomes (FGSC 2508 and 2509) using Mauve v2.3.1 (Darling et al., 2010). The alignments produced by Mauve were processed using the perl script mauve-parser.pl (http://code.google.com/p/popoolation/downloads/list) to determine the outgroup alleles for each site in the *N. tetrasperma* 2509 reference genome, using the well-established relationship of (*N. discreta* (*N. crassa*, *N. tetrasperma*)) (Corcoran et al., 2014; Dettman et al., 2003).

Phylogenomic analyses

Two different phylogenomic analyses were used to resolve the phylogenetic relationships of the *Neurospora* species and lineages included in this study. First, we carried out Maximum Likelihood phylogenomic analysis using an alignment of only variable sites from all autosomes. The set of variable sites included sites that were variable across all strains of *N. tetrasperma* and the outgroup heterothallic species, a total of 2,259,433 variable sites. The Maximum Likelihood phylogenetic inference

was carried out using RAxML v7.3.1 (Stamatakis, 2006) with the following parameters: -m GTRCAT -f d -N 20. Statistical confidence in the phylogenetic analysis was determined by 1000 bootstrap replicates.

Second, we used the STAR method (Liu et al., 2009) for estimating species trees from a collection of rooted gene trees. Orthologs were detected between *N. tetrasperma*, *N. crassa* and the *N. discreta* reference genomes as described previously by Corcoran et al., (2014) using the program InParanoid standalone v4.1 (Remm et al., 2001) and a reciprocal best BLAST hit test. This approach identified 7,619 orthologs, of which 5,723 were located on the autosomes. These orthologs were extracted from the *N. sitophila* and *N. hispaniola* consensus sequences using the annotation information from the *N. tetrasperma* 2509 reference. A subsample of two strains per *N. tetrasperma* lineage was used in this analysis, to reduce the computational time in gene tree bootstrap analysis. The orthologs were aligned using MUSCLE v3.6 (Edgar, 2004). Sites in the alignments with greater than 75% missing data were removed. Filtered alignments with less than 100bp of data were excluded from further analysis. These filtering steps resulted in 5,292 autosomal ortholog alignments. A maximum likelihood phylogenetic analysis was carried out for each ortholog using PhyML v3.0 (Guindon and Gascuel, 2003). All gene tree analyses with PhyML used the GTR + gamma model as the DNA substitution and 1000 bootstrap analysis were carried out. The tree files for each orthologs containing the bootstrap replicate trees were then used for the "species tree" reconstruction with STAR using the online server at http://bioinformatics.publichealth.uga.edu/SpeciesTreeAnalysis/index.php.

Population Structure analyses

For the InStruct analysis, we included only the strains for which we had both *mat A* and *mat a* strains from the same heterokaryon, as the data were treated as diploid (one

allele from the *mat A* homokaryons and the second allele from the *mat a* homokaryons). The Markov chain was run for 50,000 generations with 10,000 generations discarded as burnin. The Deviance Information Criterion (DIC) was used to choose the optimal number of clusters (Gao et al., 2011). The program CLUMPP v1.12 (Jakobsson and Rosenberg, 2007) was used to average across runs and to manage label switching. The program distruct (Rosenberg, 2003) was used to visualize the ancestry for each heterokaryon at the chosen K.

Determining the region of suppressed recombination (SR)

For L5, L8 and L10, where sample size was large (>8 strains) comparisons between the levels of linkage disequilibrium on the *mat* chromosome and the autosomes were used to demarcate the SR region. Where the sample size was small (≤8), the approach of Sun *et al*., (2012) was used. The divergence (proportion of nucleotide differences) between the *mat A* and *mat a* homokaryons sampled from the same heterokaryon was calculated in sliding windows of 100kb with a step size of 20kb across the autosomes. The maximum divergence observed in these windows (L1: 0.000193; L4: 0.000252; L6: 0.002173 L7: 0.00018; L9: 0.000071) was set as a cutoff value to separate the recombining flanks from the SR region on the *mat* chromosome. On the *mat* chromosome, 100kb windows flanking the regions of elevated divergence were assigned to the R region when the observed divergence was below the cutoff. The 100kb windows were further subdivided into non-overlapping 10kb windows to refine the location of the boundary or SR and R regions using the same autosomal cutoffs.

Tests for introgression between species using D-statistics

To test for introgression from heterothallic species into the autosomes of the *N. tetrasperma* lineages, we calculated the D-statistic as outlined by Green at al., (2010). The D-statistic (also referred to as the 'ABBA-BABA' test) uses the patterns from bi-allelic sites observed in three populations (P1, P2, P3) and an outgroup (O) species, for which the relationship between the populations/species is (((P1, P2), P3), O). Sites that are bi-allelic for the ancestral state 'A', as observed in O, and the derived state 'B' as observed in P3 are counted up across the genome. Under a null model excluding gene flow between P3 and P2 or P3 and P1, the expectation is no significant difference in the number of ABBA sites (P1: A, P2: B, P3: B, O: A) and the number of BABA sites (P1: B, P2: A, P3: B, O: A) across the genome, if both site patterns result from retention of ancestral polymorphism. An excess of ABBA sites would support a history of gene flow between P3 and P2, whereas an excess of BABA sites would support a history gene flow between P3 and P1.

We calculated the genome wide D-statistic from ABBA and BABA sites across the six autosomes. Here P1 and P2 were two strains sampled from different lineages of *N. tetrasperma*. We chose strain 9033 from L1 as P1 for all analyses and P2 to one of the other strains of *N. tetrasperma*, because 9033 showed no strong indication of introgression from *N. crassa*, *N. hispaniola* or *N. sitophila* in the *mat* SR divergence analysis described above (Sun et al., 2012). The *N. discreta* genome was used to determine the ancestral allele. We initially calculated the D-statistic with each *N. tetrasperma* strain as P2 and the *N. hispaniola* genome as P3, then repeated the analysis with *N. sitophila* as P3 and finally with *N. crassa* as P3. Block-jackknife resampling with a block size of 250kb was used to estimate the standard error of the D-statistic and to calculate a Z-score. The Z-score was then converted to a p-value to

determine statistical significance of the D-statistic. All p-values were corrected for multiple testing using the Bonferroni correction method implemented in R (R Development Core Team, 2014).

The pattern of ABBA and BABA sites on the *mat* chromosome was visualized using a sliding window approach that considered only sites fixed between the *mat A* and *mat a* mating-type chromosomes from the same lineage (*e.g.*, L10A and L10a). The *mat A* and *mat a* strains were treated as two populations that have diverged, we chose the *mat a* strains as P1, the *mat A* as P2 and p3 was set to either *N. hispaniola*, *N. sitophila* or *N. crassa*. *N. discreta* was used as outgroup (O) in all analyses. For example, to investigate signs of introgression from *N. hispaniola* into L10 *mat A* mating-type chromosome, we set P1 to L10a, P2 to L10A and P3 to *N. hispaniola* (see Results and Discussion). We carried out a sliding window analysis across the mating-type chromosome in 100kb windows with a 20kb step, and recorded the sites where P2 was fixed for the derived allele in P3 and P1 fixed for the ancestral allele, and *vice versa*. In the example of L10, a large excess of ABBA sites caused by introgression on L10A from *N. hispaniola* would produce a positive D-statistic much greater than zero. We refer to the D-statistic calculated on the mating-type chromosomes as D_{fixed}, as we are considering only sites where there are fixed differences between the *mat A* and *mat a* chromosomes within a lineage. This procedure was also carried out for L8 and L5 mating-type chromosomes.

Identification and alignments of orthologous genes

To investigate the levels of polymorphism and divergence in coding regions of genes, the list of orthologs used was taken from the list generated previously (Corcoran et al. 2014). For each lineage, genes were assigned to one of two groups: those that were in

recombining regions (R) of the genome and those that fell within the region of suppressed recombination (SR) on the *mat* chromosome. The annotation file for the *N. tetrasperma* 2509 reference genome V1 was used to obtain coordinates for the genes in the genome and these coordinates were used to extract gene alignments from the consensus sequences of each *N. tetrasperma* strain. In analyses that required out-group information, the *N. discreta*, *N. crassa* and *N. tetrasperma* gene orthologs were translated to proteins and aligned with MUSCLE v3.8.31 and back translated into nucleotide sequence. At this stage any genes that had a premature stop codons present were excluded. Codons within the gene alignments that had any missing data character ('N') present were removed from the alignments.

Phylogenetic analysis of the genes on the *mat* chromosome

Phylogenetic analysis of genes within the SR region and the pseudoautosomal region (PAR) of the *mat* chromosome was carried to visualize the evolutionary history of the *mat* chromosomes and the possible sources of introgression among the heterothallic species of *Neurospora*. We selected six genes that are located within the SR region and two genes located within the PAR which are common to all lineages of *N. tetrasperma* and the heterothallic species used in this study (Supplementary Table 12). Primers, PCR conditions and procedures for DNA sequencing of the chosen loci can be found in (Menkis et al., 2008). We generated alignments for each gene using the program MUSCLE v3.6 (Edgar, 2004) to align the gene sequence data from the heterothallic species with the gene sequences from the *N. tetrasperma* strains. The gene alignments were concatenated and a maximum likelihood tree was reconstructed using RAxML v7.3.1 (Stamatakis, 2006) with a GTRGAMMA model and 1000 bootstrap replicates were performed.

Phylogenetic analysis of heterokaryon incompatibility (*het*) genes

We initially sought to extract the *het-6* and *het-c* gene sequences from our reference base genotype data, but many strains lacked genotype calls at these genes due to lack of read coverage in the bam files. This could be due to the *het* alleles of these strains being highly diverged from the *het* alleles of the *N. tetrasperma* 2509 reference strain. Therefore, the *het-6* and *het-c* gene sequences were retrieved by blasting the *N. crassa het-6* and *het-c* alleles gene sequences against the *de novo* assemblies of the *N. tetrasperma*, *N. sitophila* and *N. hispaniola* genomes. The sequence from the top blast hit was extracted from each *de novo* assembly and aligned with MUSCLE V3.6. The *het-c* sequences for other heterothallic *Neurospora* species from Hall et al., (2010) were added to the *het-c* alignment, and the *het-6* sequences for *N. tetrasperma* from Powell et al., (2007) were added to the *het-6* alignment. The alignments were filtered so that sites with more than 75% missing data or gaps were removed. The Maximum Likelihood phylogenetic trees at *het-c* and *het-6* were reconstructed using PhyML with the GTR + G model and 1000 bootstrap replicates were performed.

Likelihood ratio test of divergence with gene flow in autosomes

We used a likelihood-based approach to test for gene flow between a heterothallic species and a lineage of *N. tetrasperma* using the program 3S (Yang, 2010). This program requires an outgroup (species 3) and two species (species 1 and species 2) for which the test of gene flow is applied. This program compares three different models of speciation, a null model without gene flow (M_0) and two models with gene flow (M_1 and M_2). The M_1 allows variation is speciation time across the genome, which is interpreted as gene flow, and M2 model includes a migration parameter between species 1 and species 2. Parameters estimated in all three models include

ancestral population sizes for species, θ_4 and θ_5, and speciation time τ_0 and τ_1. The τ_0 parameter is the species divergence time for the lineage ancestral to species 1, species 2 and the outgroup, while τ_1 is the species divergence time for species 1 and species 2. The divergence time parameter $\tau_i = T_i \mu$, where T_i is the divergence time on the species tree and μ is the mutation rate per site. There are two scaled population size parameters, $\theta_4 = 4N_4\mu$, the common ancestor of all three species, and $\theta_5 = 4N_5\mu$ for the species 1 and species 2, where *Ni* is ancestral N_e sizes and μ is the mutation rate per site. This program does not distinguish between isolation with migration and models of secondary contact, where gene flow (introgression) occurs between two species that have come into contact after a period of isolation from each other.

The model implemented in the 3S program requires at least one genome per species. We chose *N. discreta* as the outgroup for all triplets of species. We chose a single genome from an *N. tetrasperma* lineage as species 1 and a heterothallic species as species 2. We chose the *N. tetrasperma* genome with the highest coverage within the lineage to represent that lineage. We examined all combinations of *N. tetrasperma* lineages and heterothallic species pairs. The model assumes that the loci being consider are neutral, that there has been no recombination within loci and free recombination between loci. Given these requirements we selected intergenic loci from chromosomes 2 to 7 that were at least 1kb from the nearest gene, had a maximum length of 500bp and had a minimum length of 100bp after removing sites missing data. The intergenic loci were further filtered so that there was at least 3kb between loci. The program was run three times for each dataset to ensure consistency between runs. We used a likelihood ratio test, as described by Yang (2010), to test whether we could reject the M_0 isolation model in favor of the M_1 model with post divergence gene flow.

Simulations of sequence divergence between species

The parameter combinations for the species tree used in the McCoal simulation were:

((Species A, Species B) : τ_1 # θ_5 Species C): τ_0 # θ_4

((*N. tetrasperma* , *N. sitophila*) : 0.007743 #0.0126955, *N. discreta*) : 0.043267 #0.0717015;

((*N. tetrasperma, N. hispaniola*) : 0.004923 #0.015434, *N. discreta*) : 0.043267 #0.0717015;

((*N. tetrasperma, N. crassa*) : 0.0097225 #0.0299495, *N. discreta*): 0.043267 #0.0717015;

Supplementary references

Corcoran, P., Dettman, J.R., Sun, Y., Luque, E.M., Corrochano, L.M., Taylor, J.W., Lascoux, M., Johannesson, H., 2014. A global multilocus analysis of the model fungus Neurospora reveals a single recent origin of a novel genetic system. Mol. Phylogenet. Evol. 78, 136–147. doi:10.1016/j.ympev.2014.05.007

Darling, A.E., Mau, B., Perna, N.T., 2010. progressiveMauve: Multiple Genome Alignment with Gene Gain, Loss and Rearrangement. PLoS ONE 5, e11147. doi:10.1371/journal.pone.0011147

DePristo, M.A., Banks, E., Poplin, R., Garimella, K.V., Maguire, J.R., Hartl, C., Philippakis, A.A., Angel, G. del, Rivas, M.A., Hanna, M., McKenna, A., Fennell, T.J., Kernytsky, A.M., Sivachenko, A.Y., Cibulskis, K., Gabriel, S.B., Altshuler, D., Daly, M.J., 2011. A framework for variation discovery and genotyping using next-generation DNA sequencing data. Nat. Genet. 43, 491–498. doi:10.1038/ng.806

Dettman, J.R., Jacobson, D.J., Taylor, J.W., 2003. A multilocus genealogical approach to phylogenetic species recognition in the model eukaryote Neurospora. Evol. Int. J. Org. Evol. 57, 2703–2720.

Edgar, R.C., 2004. MUSCLE: multiple sequence alignment with high accuracy and high throughput. Nucleic Acids Res 32, 1792 – 1797.

Ellison, C.E., Stajich, J.E., Jacobson, D.J., Natvig, D.O., Lapidus, A., Foster, B., Aerts, A., Riley, R., Lindquist, E.A., Grigoriev, I.V., Taylor, J.W., 2011. Massive Changes in Genome Architecture Accompany the Transition to Self-Fertility in the Filamentous Fungus Neurospora tetrasperma. Genetics 189, 55 –69. doi:10.1534/genetics.111.130690

Gao, H., Bryc, K., Bustamante, C.D., 2011. On Identifying the Optimal Number of Population Clusters via the Deviance Information Criterion. PLoS ONE 6, e21014. doi:10.1371/journal.pone.0021014

Green, R.E., Krause, J., Briggs, A.W., Maricic, T., Stenzel, U., Kircher, M., Patterson, N., Li, H., Zhai, W., Fritz, M.H.-Y., Hansen, N.F., Durand, E.Y., Malaspinas, A.-S., Jensen, J.D., Marques-Bonet, T., Alkan, C., Prüfer, K., Meyer, M., Burbano, H.A., Good, J.M., Schultz, R., Aximu-Petri, A., Butthof, A., Höber, B., Höffner, B., Siegemund, M., Weihmann, A., Nusbaum, C., Lander, E.S., Russ, C., Novod, N., Affourtit, J., Egholm, M., Verna, C., Rudan, P., Brajkovic, D., Kucan, Ž., Gušic, I., Doronichev, V.B., Golovanova, L.V., Lalueza-Fox, C., Rasilla, M. de la, Fortea, J., Rosas, A., Schmitz, R.W., Johnson, P.L.F., Eichler, E.E., Falush, D., Birney, E., Mullikin, J.C., Slatkin, M., Nielsen, R., Kelso, J., Lachmann, M., Reich, D., Pääbo, S., 2010. A Draft Sequence of the Neandertal Genome. Science 328, 710–722. doi:10.1126/science.1188021

Guindon, S., Gascuel, O., 2003. A simple, fast, and accurate algorithm to estimate large phylogenies by maximum likelihood. Syst. Biol. 52, 696–704.

Hall, C., Welch, J., Kowbel, D.J., Glass, N.L., 2010. Evolution and Diversity of a Fungal Self/Nonself Recognition Locus. PLoS ONE 5, e14055. doi:10.1371/journal.pone.0014055

Jakobsson, M., Rosenberg, N.A., 2007. CLUMPP: a cluster matching and permutation program for dealing with label switching and multimodality in analysis of population structure. Bioinformatics 23, 1801 –1806. doi:10.1093/bioinformatics/btm233

Li, H., Durbin, R., 2010. Fast and accurate long-read alignment with Burrows-Wheeler transform. Bioinforma. Oxf. Engl. 26, 589–595. doi:10.1093/bioinformatics/btp698

Li, H., Handsaker, B., Wysoker, A., Fennell, T., Ruan, J., Homer, N., Marth, G., Abecasis, G., Durbin, R., 1000 Genome Project Data Processing Subgroup, 2009. The Sequence Alignment/Map format and SAMtools. Bioinforma. Oxf. Engl. 25, 2078–2079. doi:10.1093/bioinformatics/btp352

Li, R., Zhu, H., Ruan, J., Qian, W., Fang, X., Shi, Z., Li, Y., Li, S., Shan, G., Kristiansen, K., Li, S., Yang, H., Wang, J., Wang, J., 2010. De novo assembly of human genomes with massively parallel short read sequencing. Genome Res. 20, 265–272. doi:10.1101/gr.097261.109

Liu, L., Yu, L., Pearl, D.K., Edwards, S.V., 2009. Estimating Species Phylogenies Using Coalescence Times among Sequences. Syst. Biol. 58, 468–477. doi:10.1093/sysbio/syp031

Menkis, A., Jacobson, D.J., Gustafsson, T., Johannesson, H., 2008. The Mating-Type Chromosome in the Filamentous Ascomycete Neurospora tetrasperma Represents a Model for Early Evolution of Sex Chromosomes. PLoS Genet 4, e1000030. doi:10.1371/journal.pgen.1000030

Powell, A.J., Jacobson, D.J., Natvig, D.O., 2007. Ancestral polymorphism and linkage disequilibrium at the het-6 region in pseudohomothallic Neurospora tetrasperma. Fungal Genet. Biol. FG B 44, 896–904. doi:10.1016/j.fgb.2007.04.010

R Development Core Team, 2014. R: A language and environment for statistical computing. R Foundation for Statistical Computing, Vienna, Austria. URL http://www.R-project.org/.

Remm, M., Storm, C.E., Sonnhammer, E.L., 2001. Automatic clustering of orthologs and in-paralogs from pairwise species comparisons. J. Mol. Biol. 314, 1041–1052. doi:10.1006/jmbi.2000.5197

Rosenberg, N.A., 2003. distruct: a program for the graphical display of population structure. Mol. Ecol. Notes 4, 137–138. doi:10.1046/j.1471-8286.2003.00566.x

Stamatakis, A., 2006. RAxML-VI-HPC: maximum likelihood-based phylogenetic analyses with thousands of taxa and mixed models. Bioinforma. Oxf. Engl. 22, 2688–2690. doi:10.1093/bioinformatics/btl446

Sun, Y., Corcoran, P., Menkis, A., Whittle, C.A., Andersson, S.G.E., Johannesson, H., 2012. Large-Scale Introgression Shapes the Evolution of the Mating-Type Chromosomes of the Filamentous Ascomycete Neurospora tetrasperma. PLoS Genet. 8. doi:10.1371/journal.pgen.1002820

Yang, Z., 2010. A likelihood ratio test of speciation with gene flow using genomic sequence data. Genome Biol. Evol. 2, 200–211. doi:10.1093/gbe/evq011
