# Supplementary material for: Introgression maintains the genetic integrity of the mating-type determining chromosome of the fungus Neurospora tetrasperma
Source: Genome Res. 2016 Apr;26(4):486–98. doi: 10.1101/gr.197244.115 (PMC4817772; doi:10.1101/gr.197244.115)
Supplement: Supplemental Material [file supp_gr.197244.115_Supplemental_Tables.pdf]

Supplementary Table 1. *Neurospora* strains sequenced in this study and the mean sequencing depth across the genome.

Strain Number[1]						
Hetero-karyon	Homo-karyon[2]	Location[3]	Mating type[4]	Lineage[5]	Depth[6] 2509	Depth[6] 2508
N. tetrasperma						
RLM131	FGSC7585	Coba, Mexico	A	L4	26	25
	FGSC7586		a	L4	26	25
P4492	FGSC9033	Franklin, LA, USA	A	L1	45	43
	FGSC9034		a	L1	28	26
FGSC965	965A/FGSC10751	Liberia	A	L9	45	42
	965a/FGSC10752		a	L9	25	24
UK26	CJ01/FGSC10703	Surrey, England	A	L10	29	27
	CJ02/FGSC10704[H]		a	L10	29	27
UK12	CJ03/FGSC10705	Surrey, England	A	L10	28	27
	CJ04/FGSC10706		a	L10	28	25
UK33	CJ05/FGSC10707	Surrey, England	A	L10	28	27
	CJ06/FGSC10708		a	L10	29	26
UK43	CJ07/FGSC709[H]	Surrey, England	A	L10	27	26
	CJ08/FGSC710		a	L10	30	27
UK23	CJ09/FGSC711	Surrey, England	A	L10	27	26
	CJ10/FGSC712		a	L10	29	27
UK1	CJ11/FGSC10713[H]	Surrey, England	A	L10	28	27
	CJ12 FGSC10714[H]		a	L10	29	27
UK41	CJ13/FGSC10715	Surrey, England	A	L10	28	26
	CJ14/FGSC10716		a	L10	29	27
UK54	CJ15/FGSC10717	Surrey, England	A	L10	27	26
	CJ16/FGSC718		a	L10	28	26
UK77	CJ17/FGSC719[H]	Surrey, England	A+a	L10	28	27
	CJ18/FGSC720[H]		a	L10	29	27
UK65	CJ19/FGSC721	Surrey, England	A	L10	28	27
	CJ20/FGSC722		a	L10	29	27
UK86	CJ21/FGSC10723[H]	Surrey, England	A+a	L10	28	26
	CJ22/FGSC10724		a	L10	29	27
UK100	CJ23/FGSC10725	Surrey, England	A	L10	29	27
	CJ24/FGSC10726		a	L10	29	27
P3358	CJ25/FGSC10727	Pirongia, NZ	A	L5	29	30

	CJ26/FGSC10728		a	L5	31	29
P2252	CJ27/FGSC10729	Dargaville, NZ	A	L5	29	30
	CJ28/FGSC10730		a	L5	31	29
P2331	CJ29/FGSC10731	Te Hana, NZ	A	L5	30	31
	CJ30/FGSC10732[C2]		a	L5	31	29
P3359	CJ31/FGSC10733[H]	Pirongia, NZ	A+a	L5	29	30
	CJ32/FGSC10734		a	L5	31	28
P2317	CJ33/FGSC10735	Brookby, NZ	A	L5	29	30
	CJ34/FGSC10736		a	L5	31	29
P2275	CJ35/FGSC10737[C1]	Herekino, NZ	a	L5	31	28
	CJ36/FGSC10738		a	L5	31	29
P2284	CJ37/FGSC10739[C2]	Omapere, NZ	A	L5	30	31
	CJ38/FGSC10740		a	L5	31	29
P2253	CJ39/FGSC10741[C2]	Dargaville, NZ	A	L5	30	31
	CJ40/FGSC10742		a	L5	31	28
P2299	CJ41/FGSC10743	Waitakere, NZ	A	L5	30	30
	CJ42/FGSC10744		a	L5	30	28
P2273	CJ43/FGSC10745	Herekino, NZ	A	L5	30	31
	CJ44/FGSC10746		a	L5	31	29
P2356	CJ45	Ahipara, NZ	A	L5	29	30
	CJ46		a	L5	30	27
P2357	CJ47/FGSC10747	Ahipara, NZ	A	L5	28	29
	CJ48/FGSC10748[C1]		A	L5	27	28
P2361	CJ49/P4371	Ahipara, NZ	A	L5	28	29
	CJ50/P4372		a	L5	30	27
P2294	CJ51/FGSC10749	Mt. Albert, NZ	A	L5	30	31
P2303	CJ52/FGSC10750[H]	Cornwallis Bch,NZ	A+a	L5	31	29
P4495	CJ55/FGSC9024	Franklin, LA, USA	A	L1	33	31
	CJ56/FGSC9025		a	L1	31	30
P4488	CJ57/FGSC9026	Franklin, LA, USA	A	L8	34	32
	CJ58/FGSC9027		a	L8	30	28
P4466	CJ59/FGSC9028	Franklin, LA, USA	A	L8	30	29
	CJ60/FGSC9029		a	L8	34	32
P4460	CJ61/FGSC9030	Franklin, LA, USA	A	L8	29	28
	CJ62/FGSC9031		a	L8	31	30
J3	CJ63/FGSC9035	Franklin, LA, USA	A	L8	33	32
	CJ64/FGSC9036		a	L8	31	30
P4461	CJ65/FGSC9037[C2]	Franklin, LA, USA	A	L1	35	33
	CJ66/FGSC9038[C2]		a	L1	31	30
J14	CJ67/FGSC9039	Franklin, LA, USA	A	L8	34	33

	CJ68/FGSC9040		a	L8	30	28
P4477	CJ69/FGSC9042	Franklin, LA, USA	A	L7	34	33
	CJ70/FGSC9041		a	L7	33	31
J8	CJ71/FGSC9043	Franklin, LA, USA	A	L8	33	32
	CJ72/FGSC9044		a	L8	35	33
J2	CJ73/FGSC9045	Franklin, LA, USA	A	L7	29	27
	CJ74/FGSC9046		a	L7	32	30
J9	CJ75/FGSC9047	Franklin, LA, USA	A	L8	31	29
	CJ76/FGSC9048		a	L8	30	29
J13	CJ77/FGSC9049	Franklin, LA, USA	A	L8	32	30
	CJ78/FGSC9050		a	L8	33	31
P4379	CJ79/FGSC9051	Welsh, LA, USA	A	L8	32	30
P4380	CJ80/FGSC9052		a	L8	31	30
P4381	CJ81/FGSC9053	Welsh, LA, USA	A	L7	29	28
P4382	CJ82/FGSC9054		a	L7	28	27
P470	CJ83/FGSC2501	Empire, LA, USA	A	L7	30	28
unknown	CJ84/FGSC2502		a	L7	33	31
P505	CJ85/FGSC2503	Welsh, LA, USA	A	L8	33	31
	CJ86/FGSC2504		a	L8	32	30
P0654	CJ87/FGSC2505[H]	Perkins, LA, USA	A+a	L8	32	30
P0655	CJ88/FGSC2506[H]		A+a	L8	30	29
	N. sitophila					
NA	FGSC1135	Panama	A	NA	32	30
	N. hispaniola					
NA	FGSC8817	Carrefour Dufort, Haiti	A	NA	31	30

1. Strain Names are Fungal Genetic Stock Center (FGSC), Perkins collection (P), Jacobson collection (J), RL Metzenberg (RLM) and UK/CJ numbers are from Corcoran et al (2012).
2. Genomes excluded from analyses due to "H": heterokaryosis, "C": identity (clonality) with other strain originating from the same heterokaryon "C1" or a different heterokaryon "C2."
3. Abbreviations: Louisiana (LA); New Zealand (NZ).
4. Mating type is either *mat A* (A), *mat a* (a) or heterokaryotic (A+a).
5. Lineage to which the *N. tetrasperma* strains are assigned, based on Menkis *et al.* (2009) and Corcoran *et al.* (2014).

6. *Neurospora tetrasperma* has two reference genomes from L6. The FGSC 2508 (*mat A*) and 2509 (*mat a*) homokarons were isolated from P581 (see Ellison et al. (2011), for details). Sequencing depths were calculated using the samtools depth command.

Supplementary Table 2. Comparison of genetic variation in *N. tetrasperma* and *N. crassa*[1]. Nucleotide diversity *(π*) of autosomes, and distance over which linkage disequilibrium (LD) decays to half its maximum.

Population	π	LD decay (kb)
L10 (UK)	0.0050	>500
L5 (NZ)	0.0022	11
L8 (LA)	0.0008	31
N. crassa (Louisiana)	0.0024	0.70
N. crassa (Caribbean)	0.0023	0.85

[1]Data for *N. crassa* Ellison *et al.* 2011a.

Supplementary Table 3. Mean nucleotide diversity at synonymous sites (π_s) across genes in recombining regions (R) and regions of suppressed recombination (SR) of the genome in Lineages of *N. tetrasperma*.

Lineage	R	SR (*mat A*)	SR (*mat a*)	R/SR[1] (*mat* A)	R/SR[1] (*mat* a)
L10 (UK)	0.01061	0.00007	0.00008	75.8	66.7
L5 (NZ)	0.00424	0.00065	0.00043	3.3	4.9
L8 (LA)	0.00194	0.00078	0.00004	1.24	24.25

[1]These ratios are divided by a factor of two to account for half the effective population size in the SR compared to R.

Supplementary Table 4. Divergence between the *N. tetrasperma* lineages and the three heterothallic species within the recombining regions of the genome (R) and regions of suppressed recombination on the *mat* chromosome. Divergence values in SR regions that are more extreme than expected under the isolation model are highlighted in bold.

		N. crassa		*N. hispaniola*		*N. sitophila*	
Lineage	Region[1]	Median	p-value[2]	Median	p-value	Median	p-value
L1	R	0.0434	n/a	0.0235	n/a	0.0264	n/a
L1	SRa	0.0353	0.4271	0.0197	0.48298	0.0248	0.53406
L1	SRA	0.0363	0.4469	0.0208	0.51916	0.0211	0.37128
L4	R	0.0436	n/a	0.0225	n/a	0.0267	n/a
L4	SRa	0.0369	0.4586	**0.0058**	**0**	0.0269	0.39266
L4	SRA	0.0374	0.4685	0.0205	0.50928	0.0227	0.50928
L5	R	0.0459	n/a	0.0248	n/a	0.0274	n/a
L5	SRa	0.0387	0.49224	0.0216	0.54458	0.0220	0.4151
L5	SRA	0.0362	0.55482	0.0302	0.2575	0.0352	0.19812
L6	R	0.0476	n/a	0.0252	n/a	0.0278	n/a
L6	SRa	0.0389	0.50424	0.0220	0.5567	0.0225	0.15882
L6	SRA	0.0400	0.4853	0.0320	0.22828	0.0379	0.43722
L7	R	0.0416	n/a	0.0216	n/a	0.0259	n/a
L7	SRa	0.0345	0.40986	**0.0074**	**0**	0.0247	0.52916
L7	SRA	0.0344	0.41134	0.0186	0.44436	0.0236	0.48504
L8	R	0.0423	n/a	0.0222	n/a	0.0257	n/a
L8	SRa	0.0360	0.44184	0.0208	0.51916	0.0210	0.36798
L8	SRA	0.0357	0.43556	0.0192	0.46582	0.0233	0.47148
L9	R	0.0444	n/a	0.0239	n/a	0.0268	n/a
L9	SRa	0.0275	0.25022	0.0300	0.26102	0.0342	0.2156
L9	SRA	0.0370	0.46088	0.0216	0.4564	0.0222	0.4242
L10	R	0.0440	n/a	0.0234	n/a	0.0272	n/a
L10	SRa	0.0377	0.47318	0.0213	0.5345	0.0211	0.37128
L10	SRA	0.0369	0.4586	**0.0047**	**0**	0.0280	0.35892

[1.]The R region is composed of pseudoautosomal regions (PAR) and autosomes.

[2] This is the proportion of simulated sequence divergence values that were as extreme or more extreme than the observed median divergence to a heterothallic species (50000 replicate simulations of 25kb a locus). Paramaters used as input for the MCcoal simulations were taken from the estimates produced with the M0 model in 3S run on autosomal intergenic regions (see methods for further details).

[3] n/a is not applicable.

Supplementary Table 5. Patterson's *D*-statistic (D), which measures excess of ABBA sites over BABA sites or vice versa , for the comparison of strain 9033 (P1) of L1 *N. tetrasperma* (L1 column), and a strain (P2) from each of the other seven lineages (L2 column) *of N. tetrasperma*, 8817 from *N. hispaniola* (P3) and *N. discreta* as the outgroups (O). A significantly positive D-statistic supports introgression from *N. hispaniola* and P2.

L1	L2	P1	P2	P3	O	D	Standard Error	Z-score	p-value
L1	L6	9033	2508	8817	Ndis	0.0352	0.0126	2.8010	3.77E-01
L1	L6	9033	2509	8817	Ndis	0.0383	0.0127	3.0243	1.84E-01
L1	L4	9033	7585	8817	Ndis	0.0934	0.0165	5.6636	1.10E-06
L1	L4	9033	7586	8817	Ndis	0.0942	0.0166	5.6708	1.05E-06
L1	L9	9033	965a	8817	Ndis	-0.0438	0.0110	-3.9995	4.70E-03
L1	L9	9033	965BA	8817	Ndis	-0.0439	0.0107	-4.1196	2.81E-03
L1	L10	9033	CJ01	8817	Ndis	0.0978	0.0134	7.3088	2.00E-11
L1	L10	9033	CJ03	8817	Ndis	0.0875	0.0178	4.8996	7.10E-05
L1	L10	9033	CJ04	8817	Ndis	0.0838	0.0181	4.6413	2.56E-04
L1	L10	9033	CJ05	8817	Ndis	0.0807	0.0200	4.0373	4.00E-03
L1	L10	9033	CJ06	8817	Ndis	0.3841	0.0548	7.0115	1.75E-10
L1	L10	9033	CJ08	8817	Ndis	0.0754	0.0153	4.9410	5.75E-05
L1	L10	9033	CJ09	8817	Ndis	0.3805	0.0551	6.9114	3.55E-10
L1	L10	9033	CJ10	8817	Ndis	0.0819	0.0186	4.4119	7.55E-04
L1	L10	9033	CJ13	8817	Ndis	0.0691	0.0155	4.4521	6.29E-04
L1	L10	9033	CJ14	8817	Ndis	0.0890	0.0137	6.4954	6.13E-09
L1	L10	9033	CJ15	8817	Ndis	0.0911	0.0168	5.4154	4.53E-06
L1	L10	9033	CJ16	8817	Ndis	0.3994	0.0550	7.2557	2.95E-11
L1	L10	9033	CJ19	8817	Ndis	0.3898	0.0549	7.0944	9.62E-11
L1	L10	9033	CJ20	8817	Ndis	0.0804	0.0191	4.2023	1.95E-03
L1	L10	9033	CJ22	8817	Ndis	0.0825	0.0189	4.3730	9.10E-04
L1	L10	9033	CJ23	8817	Ndis	0.0855	0.0190	4.4916	5.23E-04
L1	L10	9033	CJ24	8817	Ndis	0.0697	0.0203	3.4342	4.40E-02
L1	L5	9033	CJ25	8817	Ndis	0.0048	0.0120	0.3993	1.00E+00
L1	L5	9033	CJ26	8817	Ndis	0.0037	0.0118	0.3169	1.00E+00
L1	L5	9033	CJ27	8817	Ndis	0.0029	0.0116	0.2536	1.00E+00
L1	L5	9033	CJ28	8817	Ndis	0.0025	0.0116	0.2141	1.00E+00
L1	L5	9033	CJ29	8817	Ndis	0.0040	0.0121	0.3300	1.00E+00
L1	L5	9033	CJ32	8817	Ndis	0.0047	0.0112	0.4232	1.00E+00
L1	L5	9033	CJ33	8817	Ndis	0.0095	0.0122	0.7810	1.00E+00
L1	L5	9033	CJ34	8817	Ndis	0.0101	0.0122	0.8257	1.00E+00
L1	L5	9033	CJ36	8817	Ndis	0.0047	0.0111	0.4280	1.00E+00
L1	L5	9033	CJ38	8817	Ndis	0.0050	0.0116	0.4292	1.00E+00
L1	L5	9033	CJ40	8817	Ndis	0.0095	0.0115	0.8231	1.00E+00
L1	L5	9033	CJ41	8817	Ndis	0.0045	0.0118	0.3859	1.00E+00

L1	L5	9033	CJ42	8817	Ndis	0.0045	0.0116	0.3898	1.00E+00
L1	L5	9033	CJ43	8817	Ndis	0.0034	0.0118	0.2890	1.00E+00
L1	L5	9033	CJ44	8817	Ndis	0.0050	0.0116	0.4264	1.00E+00
L1	L5	9033	CJ45	8817	Ndis	0.0101	0.0120	0.8423	1.00E+00
L1	L5	9033	CJ46	8817	Ndis	0.0137	0.0120	1.1380	1.00E+00
L1	L5	9033	CJ47	8817	Ndis	0.0046	0.0121	0.3826	1.00E+00
L1	L5	9033	CJ49	8817	Ndis	0.0077	0.0118	0.6534	1.00E+00
L1	L5	9033	CJ50	8817	Ndis	0.0069	0.0117	0.5901	1.00E+00
L1	L5	9033	CJ51	8817	Ndis	0.0032	0.0111	0.2925	1.00E+00
L1	L1	9033	CJ55	8817	Ndis	0.0047	0.0351	0.1325	1.00E+00
L1	L1	9033	CJ56	8817	Ndis	0.0090	0.0336	0.2692	1.00E+00
L1	L8	9033	CJ57	8817	Ndis	0.0744	0.0136	5.4648	3.43E-06
L1	L8	9033	CJ58	8817	Ndis	0.0752	0.0136	5.5090	2.67E-06
L1	L8	9033	CJ59	8817	Ndis	0.0695	0.0140	4.9563	5.32E-05
L1	L8	9033	CJ60	8817	Ndis	0.0692	0.0138	5.0064	4.11E-05
L1	L8	9033	CJ61	8817	Ndis	0.0767	0.0138	5.5539	2.06E-06
L1	L8	9033	CJ62	8817	Ndis	0.0751	0.0137	5.4733	3.27E-06
L1	L8	9033	CJ63	8817	Ndis	0.0739	0.0136	5.4466	3.80E-06
L1	L8	9033	CJ64	8817	Ndis	0.0746	0.0136	5.4958	2.88E-06
L1	L8	9033	CJ67	8817	Ndis	0.0675	0.0139	4.8413	9.55E-05
L1	L8	9033	CJ68	8817	Ndis	0.0703	0.0139	5.0636	3.04E-05
L1	L7	9033	CJ69	8817	Ndis	0.0461	0.0128	3.5924	2.42E-02
L1	L7	9033	CJ70	8817	Ndis	0.0464	0.0129	3.5892	2.45E-02
L1	L8	9033	CJ71	8817	Ndis	0.0717	0.0139	5.1469	1.96E-05
L1	L8	9033	CJ72	8817	Ndis	0.0711	0.0140	5.0939	2.60E-05
L1	L7	9033	CJ73	8817	Ndis	0.0939	0.0132	7.1346	7.19E-11
L1	L7	9033	CJ74	8817	Ndis	0.0942	0.0132	7.1433	6.74E-11
L1	L8	9033	CJ75	8817	Ndis	0.0688	0.0138	4.9772	4.77E-05
L1	L8	9033	CJ76	8817	Ndis	0.0688	0.0139	4.9599	5.22E-05
L1	L8	9033	CJ77	8817	Ndis	0.0759	0.0141	5.3998	4.94E-06
L1	L8	9033	CJ78	8817	Ndis	0.0764	0.0141	5.4177	4.47E-06
L1	L8	9033	CJ79	8817	Ndis	0.0735	0.0138	5.3392	6.91E-06
L1	L8	9033	CJ80	8817	Ndis	0.0740	0.0137	5.4059	4.77E-06
L1	L7	9033	CJ81	8817	Ndis	0.1007	0.0131	7.6911	1.09E-12
L1	L7	9033	CJ82	8817	Ndis	0.1007	0.0131	7.7091	9.40E-13
L1	L7	9033	CJ83	8817	Ndis	0.0982	0.0131	7.4945	4.93E-12
L1	L7	9033	CJ84	8817	Ndis	0.0977	0.0131	7.4344	7.77E-12
L1	L8	9033	CJ85	8817	Ndis	0.0752	0.0137	5.4898	2.97E-06
L1	L8	9033	CJ86	8817	Ndis	0.0752	0.0138	5.4599	3.52E-06

Supplementary Table 6. Patterson's *D*-statistic (D), which measures excess of ABBA sites over BABA sites or vice versa, for the comparison of strain 9033 (P1) of L1 *N. tetrasperma* (L1 column), a single strain (P2) from each of the other seven lineages (L2 column) of *N. tetrasperma*, 1135 from *N. sitophila* (P3) and *N. discreta* as the outgroups (O). A significantly positive D-statistic supports introgression between *N. sitophila* and P2.

L1	L2	P1	P2	P3	O	Dstat	SE	Z	p-value
L1	L6	9033	2508	1135	Ndis	0.0743	0.0120	6.1768	4.84E-08
L1	L6	9033	2509	1135	Ndis	0.0772	0.0120	6.4369	9.03E-09
L1	L4	9033	7585	1135	Ndis	-0.0437	0.0178	-2.4548	1.00E+00
L1	L4	9033	7586	1135	Ndis	-0.0439	0.0181	-2.4307	1.00E+00
L1	L9	9033	965a	1135	Ndis	-0.0099	0.0121	-0.8207	1.00E+00
L1	L9	9033	965BA	1135	Ndis	-0.0103	0.0122	-0.8431	1.00E+00
L1	L10	9033	CJ01	1135	Ndis	-0.0410	0.0112	-3.6506	1.94E-02
L1	L10	9033	CJ03	1135	Ndis	-0.0612	0.0185	-3.3099	6.91E-02
L1	L10	9033	CJ04	1135	Ndis	-0.0604	0.0176	-3.4383	4.33E-02
L1	L10	9033	CJ05	1135	Ndis	-0.0341	0.0230	-1.4850	1.00E+00
L1	L10	9033	CJ06	1135	Ndis	-0.1824	0.0307	-5.9503	1.98E-07
L1	L10	9033	CJ08	1135	Ndis	-0.0328	0.0180	-1.8247	1.00E+00
L1	L10	9033	CJ09	1135	Ndis	-0.1590	0.0342	-4.6430	2.54E-04
L1	L10	9033	CJ10	1135	Ndis	-0.0341	0.0224	-1.5231	1.00E+00
L1	L10	9033	CJ13	1135	Ndis	-0.0196	0.0164	-1.1976	1.00E+00
L1	L10	9033	CJ14	1135	Ndis	-0.0368	0.0111	-3.3117	6.86E-02
L1	L10	9033	CJ15	1135	Ndis	-0.0629	0.0191	-3.2861	7.52E-02
L1	L10	9033	CJ16	1135	Ndis	-0.1568	0.0365	-4.2982	1.27E-03
L1	L10	9033	CJ19	1135	Ndis	-0.1637	0.0358	-4.5735	3.55E-04
L1	L10	9033	CJ20	1135	Ndis	-0.0317	0.0228	-1.3891	1.00E+00
L1	L10	9033	CJ22	1135	Ndis	-0.0661	0.0189	-3.4906	3.57E-02
L1	L10	9033	CJ23	1135	Ndis	-0.0623	0.0191	-3.2621	8.18E-02
L1	L10	9033	CJ24	1135	Ndis	-0.0360	0.0227	-1.5872	1.00E+00
L1	L5	9033	CJ25	1135	Ndis	0.0459	0.0120	3.8115	1.02E-02
L1	L5	9033	CJ26	1135	Ndis	0.0447	0.0121	3.6809	1.72E-02
L1	L5	9033	CJ27	1135	Ndis	0.0450	0.0121	3.7095	1.54E-02
L1	L5	9033	CJ28	1135	Ndis	0.0450	0.0121	3.7328	1.40E-02
L1	L5	9033	CJ29	1135	Ndis	0.0435	0.0123	3.5393	2.97E-02
L1	L5	9033	CJ32	1135	Ndis	0.0425	0.0121	3.5224	3.16E-02
L1	L5	9033	CJ33	1135	Ndis	0.0487	0.0118	4.1148	2.87E-03
L1	L5	9033	CJ34	1135	Ndis	0.0487	0.0119	4.1039	3.00E-03
L1	L5	9033	CJ36	1135	Ndis	0.0422	0.0120	3.5104	3.31E-02
L1	L5	9033	CJ38	1135	Ndis	0.0468	0.0117	4.0168	4.37E-03
L1	L5	9033	CJ40	1135	Ndis	0.0420	0.0122	3.4455	4.22E-02
L1	L5	9033	CJ41	1135	Ndis	0.0448	0.0121	3.7149	1.50E-02

L1	L5	9033	CJ42	1135	Ndis	0.0460	0.0120	3.8217	9.81E-03
L1	L5	9033	CJ43	1135	Ndis	0.0416	0.0121	3.4377	4.34E-02
L1	L5	9033	CJ44	1135	Ndis	0.0421	0.0120	3.5005	3.44E-02
L1	L5	9033	CJ45	1135	Ndis	0.0447	0.0121	3.6881	1.67E-02
L1	L5	9033	CJ46	1135	Ndis	0.0478	0.0123	3.8905	7.40E-03
L1	L5	9033	CJ47	1135	Ndis	0.0452	0.0121	3.7319	1.41E-02
L1	L5	9033	CJ49	1135	Ndis	0.0477	0.0119	3.9960	4.77E-03
L1	L5	9033	CJ50	1135	Ndis	0.0469	0.0120	3.9061	6.94E-03
L1	L5	9033	CJ51	1135	Ndis	0.0452	0.0120	3.7670	1.22E-02
L1	L1	9033	CJ55	1135	Ndis	-0.0102	0.0360	-0.2827	1.00E+00
L1	L1	9033	CJ56	1135	Ndis	-0.0143	0.0354	-0.4045	1.00E+00
L1	L8	9033	CJ57	1135	Ndis	0.0022	0.0177	0.1242	1.00E+00
L1	L8	9033	CJ58	1135	Ndis	0.0028	0.0178	0.1575	1.00E+00
L1	L8	9033	CJ59	1135	Ndis	0.0023	0.0176	0.1316	1.00E+00
L1	L8	9033	CJ60	1135	Ndis	0.0010	0.0176	0.0572	1.00E+00
L1	L8	9033	CJ61	1135	Ndis	0.0013	0.0178	0.0704	1.00E+00
L1	L8	9033	CJ62	1135	Ndis	0.0000	0.0178	0.0000	1.00E+00
L1	L8	9033	CJ63	1135	Ndis	0.0075	0.0175	0.4262	1.00E+00
L1	L8	9033	CJ64	1135	Ndis	0.0090	0.0175	0.5138	1.00E+00
L1	L8	9033	CJ67	1135	Ndis	0.0010	0.0178	0.0553	1.00E+00
L1	L8	9033	CJ68	1135	Ndis	0.0032	0.0177	0.1787	1.00E+00
L1	L7	9033	CJ69	1135	Ndis	-0.1516	0.0191	-7.9488	1.32E-13
L1	L7	9033	CJ70	1135	Ndis	-0.1520	0.0190	-8.0117	8.21E-14
L1	L8	9033	CJ71	1135	Ndis	0.0042	0.0175	0.2422	1.00E+00
L1	L8	9033	CJ72	1135	Ndis	0.0032	0.0175	0.1820	1.00E+00
L1	L7	9033	CJ73	1135	Ndis	-0.0936	0.0180	-5.1894	1.56E-05
L1	L7	9033	CJ74	1135	Ndis	-0.0936	0.0180	-5.1854	1.60E-05
L1	L8	9033	CJ75	1135	Ndis	0.0011	0.0177	0.0605	1.00E+00
L1	L8	9033	CJ76	1135	Ndis	0.0008	0.0176	0.0454	1.00E+00
L1	L8	9033	CJ77	1135	Ndis	0.0035	0.0180	0.1970	1.00E+00
L1	L8	9033	CJ78	1135	Ndis	0.0033	0.0179	0.1843	1.00E+00
L1	L8	9033	CJ79	1135	Ndis	0.0059	0.0178	0.3298	1.00E+00
L1	L8	9033	CJ80	1135	Ndis	0.0063	0.0179	0.3548	1.00E+00
L1	L7	9033	CJ81	1135	Ndis	-0.0940	0.0181	-5.2042	1.44E-05
L1	L7	9033	CJ82	1135	Ndis	-0.0942	0.0180	-5.2300	1.25E-05
L1	L7	9033	CJ83	1135	Ndis	-0.0946	0.0180	-5.2687	1.01E-05
L1	L7	9033	CJ84	1135	Ndis	-0.0943	0.0179	-5.2668	1.03E-05
L1	L8	9033	CJ85	1135	Ndis	0.0042	0.0178	0.2368	1.00E+00
L1	L8	9033	CJ86	1135	Ndis	0.0053	0.0177	0.2962	1.00E+00

Supplementary Table 7. Patterson's *D*-statistic (D), which measures excess of ABBA sites over BABA sites or vice versa, for the comparison of strain 9033 (P1) of L1 *N. tetrasperma* (L1 column), a single strain (P2) from each of the other seven lineages (L2 column) of *N. tetrasperma*, from *N. crassa* (P3) and *N. discreta* as the outgroups (O). A significantly positive D-statistic supports introgression between *N. crassa* and P2.

L1	L2	P1	P2	P3	O	Dstat	SE	Z	p-value
L1	L6	9033	2508	Ncra	Ndis	-0.0027	0.0076	-0.3536	1.00E+00
L1	L6	9033	2509	Ncra	Ndis	-0.0024	0.0076	-0.3112	1.00E+00
L1	L4	9033	7585	Ncra	Ndis	-0.0219	0.0072	-3.0510	1.00E+00
L1	L4	9033	7586	Ncra	Ndis	-0.0217	0.0071	-3.0434	1.00E+00
L1	L9	9033	965a	Ncra	Ndis	-0.0049	0.0066	-0.7334	1.00E+00
L1	L9	9033	965BA	Ncra	Ndis	-0.0045	0.0065	-0.6820	1.00E+00
L1	L10	9033	CJ01	Ncra	Ndis	0.0093	0.0068	1.3538	1.00E+00
L1	L10	9033	CJ03	Ncra	Ndis	0.0007	0.0072	0.0973	1.00E+00
L1	L10	9033	CJ04	Ncra	Ndis	0.0015	0.0073	0.2096	1.00E+00
L1	L10	9033	CJ05	Ncra	Ndis	0.0000	0.0077	-0.0051	1.00E+00
L1	L10	9033	CJ06	Ncra	Ndis	-0.0075	0.0077	-0.9645	1.00E+00
L1	L10	9033	CJ08	Ncra	Ndis	-0.0024	0.0059	-0.3976	1.00E+00
L1	L10	9033	CJ09	Ncra	Ndis	-0.0088	0.0077	-1.1401	1.00E+00
L1	L10	9033	CJ10	Ncra	Ndis	0.0006	0.0071	0.0914	1.00E+00
L1	L10	9033	CJ13	Ncra	Ndis	0.0051	0.0066	0.7662	1.00E+00
L1	L10	9033	CJ14	Ncra	Ndis	0.0012	0.0063	0.1903	1.00E+00
L1	L10	9033	CJ15	Ncra	Ndis	0.0111	0.0073	1.5131	1.00E+00
L1	L10	9033	CJ16	Ncra	Ndis	-0.0137	0.0077	-1.7877	1.00E+00
L1	L10	9033	CJ19	Ncra	Ndis	-0.0150	0.0075	-1.9880	1.00E+00
L1	L10	9033	CJ20	Ncra	Ndis	-0.0014	0.0073	-0.1903	1.00E+00
L1	L10	9033	CJ22	Ncra	Ndis	-0.0027	0.0080	-0.3365	1.00E+00
L1	L10	9033	CJ23	Ncra	Ndis	-0.0009	0.0077	-0.1143	1.00E+00
L1	L10	9033	CJ24	Ncra	Ndis	-0.0001	0.0076	-0.0147	1.00E+00
L1	L5	9033	CJ25	Ncra	Ndis	0.0106	0.0070	1.5050	1.00E+00
L1	L5	9033	CJ26	Ncra	Ndis	0.0098	0.0069	1.4299	1.00E+00
L1	L5	9033	CJ27	Ncra	Ndis	0.0083	0.0067	1.2372	1.00E+00
L1	L5	9033	CJ28	Ncra	Ndis	0.0086	0.0067	1.2789	1.00E+00
L1	L5	9033	CJ29	Ncra	Ndis	0.0092	0.0069	1.3346	1.00E+00
L1	L5	9033	CJ32	Ncra	Ndis	0.0086	0.0066	1.2981	1.00E+00
L1	L5	9033	CJ33	Ncra	Ndis	0.0091	0.0071	1.2871	1.00E+00
L1	L5	9033	CJ34	Ncra	Ndis	0.0099	0.0070	1.4062	1.00E+00
L1	L5	9033	CJ38	Ncra	Ndis	0.0062	0.0070	0.8864	1.00E+00
L1	L5	9033	CJ40	Ncra	Ndis	0.0057	0.0070	0.8099	1.00E+00
L1	L5	9033	CJ41	Ncra	Ndis	0.0089	0.0067	1.3328	1.00E+00
L1	L5	9033	CJ42	Ncra	Ndis	0.0086	0.0068	1.2526	1.00E+00

L1	L5	9033	CJ43	Ncra	Ndis	0.0046	0.0071	0.6437	1.00E+00
L1	L5	9033	CJ44	Ncra	Ndis	0.0050	0.0070	0.7072	1.00E+00
L1	L5	9033	CJ45	Ncra	Ndis	0.0066	0.0069	0.9576	1.00E+00
L1	L5	9033	CJ46	Ncra	Ndis	0.0063	0.0068	0.9224	1.00E+00
L1	L5	9033	CJ47	Ncra	Ndis	0.0076	0.0072	1.0500	1.00E+00
L1	L5	9033	CJ49	Ncra	Ndis	0.0053	0.0070	0.7640	1.00E+00
L1	L5	9033	CJ50	Ncra	Ndis	0.0034	0.0069	0.4962	1.00E+00
L1	L1	9033	CJ55	Ncra	Ndis	0.0112	0.0203	0.5499	1.00E+00
L1	L1	9033	CJ56	Ncra	Ndis	0.0103	0.0191	0.5385	1.00E+00
L1	L8	9033	CJ57	Ncra	Ndis	0.0021	0.0066	0.3226	1.00E+00
L1	L8	9033	CJ58	Ncra	Ndis	0.0021	0.0066	0.3185	1.00E+00
L1	L8	9033	CJ59	Ncra	Ndis	0.0002	0.0066	0.0327	1.00E+00
L1	L8	9033	CJ60	Ncra	Ndis	-0.0005	0.0066	-0.0813	1.00E+00
L1	L8	9033	CJ61	Ncra	Ndis	-0.0021	0.0067	-0.3146	1.00E+00
L1	L8	9033	CJ62	Ncra	Ndis	-0.0039	0.0068	-0.5795	1.00E+00
L1	L8	9033	CJ63	Ncra	Ndis	0.0029	0.0063	0.4685	1.00E+00
L1	L8	9033	CJ64	Ncra	Ndis	0.0029	0.0063	0.4578	1.00E+00
L1	L8	9033	CJ67	Ncra	Ndis	-0.0017	0.0067	-0.2529	1.00E+00
L1	L8	9033	CJ68	Ncra	Ndis	-0.0019	0.0068	-0.2714	1.00E+00
L1	L7	9033	CJ69	Ncra	Ndis	-0.0119	0.0072	-1.6629	1.00E+00
L1	L7	9033	CJ70	Ncra	Ndis	-0.0119	0.0071	-1.6687	1.00E+00
L1	L8	9033	CJ71	Ncra	Ndis	0.0022	0.0064	0.3409	1.00E+00
L1	L8	9033	CJ72	Ncra	Ndis	0.0022	0.0065	0.3303	1.00E+00
L1	L7	9033	CJ73	Ncra	Ndis	-0.0007	0.0072	-0.1024	1.00E+00
L1	L7	9033	CJ74	Ncra	Ndis	-0.0013	0.0071	-0.1864	1.00E+00
L1	L8	9033	CJ75	Ncra	Ndis	-0.0012	0.0067	-0.1825	1.00E+00
L1	L8	9033	CJ76	Ncra	Ndis	-0.0013	0.0066	-0.1917	1.00E+00
L1	L8	9033	CJ77	Ncra	Ndis	-0.0015	0.0068	-0.2243	1.00E+00
L1	L8	9033	CJ78	Ncra	Ndis	-0.0012	0.0068	-0.1741	1.00E+00
L1	L8	9033	CJ79	Ncra	Ndis	0.0010	0.0066	0.1474	1.00E+00
L1	L8	9033	CJ80	Ncra	Ndis	0.0012	0.0065	0.1844	1.00E+00
L1	L7	9033	CJ81	Ncra	Ndis	0.0000	0.0069	0.0053	1.00E+00
L1	L7	9033	CJ82	Ncra	Ndis	0.0005	0.0068	0.0696	1.00E+00
L1	L7	9033	CJ83	Ncra	Ndis	0.0003	0.0069	0.0422	1.00E+00
L1	L7	9033	CJ84	Ncra	Ndis	0.0007	0.0068	0.1080	1.00E+00
L1	L8	9033	CJ85	Ncra	Ndis	0.0001	0.0067	0.0217	1.00E+00
L1	L8	9033	CJ86	Ncra	Ndis	0.0013	0.0066	0.1908	1.00E+00

Supplementary Table 8 Maximum likelihood estimates of parameters under Model M1 and the likelihood ratio test statistic (2Δ*l*) between M1 (model with gene flow) and M0 (isolation model) for species divergence between lineages of *N. tetrasperma* and heterothallics species. Parameters θ and τ are scaled by 100.

Species Triplet	Loci	2Δ*l* [1]	M0				M1			
((1,2),Outgroup)			θ_4	θ_5	τ_0	τ_1	θ_4	θ_5	τ_0	τ_1
((L1, *N. sit*), *N. dis*)	719	**19.23**	7.06	1.25	4.32	0.76	7.11	1.11	4.11	0.64
((L4, *N. sit*), *N. dis*)	725	**13.48**	7.17	1.25	4.32	0.81	6.92	1.22	4.16	0.63
((L5, *N. sit*), *N. dis*)	713	**16.33**	7.37	1.45	4.33	0.75	7.19	1.24	4.19	0.64
((L6, *N. sit*), *N. dis*)	728	**10.86**	7.55	1.88	4.33	0.81	7.40	1.04	4.18	0.83
((L7, *N. sit*), *N. dis*)	732	**4.50**	7.02	1.28	4.30	0.80	6.99	1.25	4.20	0.64
((L8, *N. sit*), *N. dis*)	710	**11.72**	7.08	1.25	4.26	0.75	7.95	1.52	4.15	0.79
((L9, *N. sit*), *N. dis*)	712	**10.19**	7.16	1.25	4.34	0.79	6.97	1.23	4.23	0.68
((L10, *N. sit*), *N. dis*)	719	**11.00**	7.07	1.43	4.35	0.73	7.54	0.95	4.12	0.73
((L1, *N. his*), *N. dis*)	719	**4.92**	6.97	1.45	4.20	0.54	7.04	1.14	4.29	0.88
((L4, *N. his*), *N. dis*)	714	**13.86**	7.17	1.56	4.20	0.48	7.59	1.58	4.31	0.96
((L5, *N. his*), *N. dis*)	732	**20.92**	7.31	1.70	4.21	0.50	7.12	1.00	4.24	0.87
((L6, *N. his*), *N. dis*)	737	**20.46**	7.90	1.98	4.17	0.57	7.20	0.90	4.30	0.99
((L7, *N. his*), *N. dis*)	705	**13.90**	6.90	1.53	4.17	0.47	7.11	0.84	4.30	0.96
((L8, *N. his*), *N. dis*)	712	**13.19**	7.09	1.52	4.12	0.44	7.20	1.00	4.33	0.91
((L9, *N. his*), *N. dis*)	714	**7.49**	6.95	1.49	4.24	0.55	7.10	1.06	4.33	0.92
((L10, *N. his*), *N. dis*)	719	**31.10**	7.46	1.70	4.14	0.36	7.41	0.96	4.30	0.99
((L1,*N. cra*), *N. dis*))	772	**5.86**	7.45	2.96	4.64	0.97	6.96	2.65	4.66	1.09
((L4,*N. cra*), *N. dis*))	756	**5.04**	6.93	2.87	4.67	0.99	8.14	3.55	4.63	1.03
((L5,*N. cra*), *N. dis*))	786	2.11	7.99	3.31	4.63	1.00	8.02	3.19	4.63	1.05
((L6,*N. cra*), *N. dis*))	796	1.44	8.12	3.69	4.64	0.97	7.42	2.86	4.56	0.96
((L7,*N. cra*), *N. dis*))	756	0.05	7.41	2.88	4.57	0.96	7.76	2.98	4.59	1.01
((L8,*N. cra*), *N. dis*))	761	1.27	7.15	2.86	4.60	0.97	7.16	2.76	4.59	1.01
((L9,*N. cra*), *N. dis*))	769	**2.77**	7.67	3.03	4.61	1.00	7.51	2.74	4.62	1.08
((L10,*N. cra*), *N. dis*))	773	2.37	7.73	3.14	4.60	0.94	7.70	2.89	4.60	1.06

[1]Statistically significant results are in bold (5% critical value 2.71 (Yang 2010))

Supplementary Table 9. Counts of synonymous polymorphisms (S) to non-synonymous polymorphisms (N) in recombining (R) and non-recombining region (SR) of *N. tetrasperma*.

Lineage	Polymorphism type	Region		***P-value***
		**SR A**	**R**	
L5 (NZ)	N	766	11522	2.2e-16
	S	960	25147	
L8 (LA)	N	791	5699	2.2e-16
	S	782	11046	
L10 (UK)	N	103	20997	1.73e-07
	S	131	54043	
		**SR a**	**R**	
L5 (NZ)	N	797	11522	2.2e-16
	S	835	25147	
L8 (LA)	N	120	5699	7.063e-10
	S	100	11046	
L10 (UK)	N	115	20997	2.2e-16
	S	85	54043	
		**SR A**	**SR a**	
L5 (NZ)	N	766	797	0.01043
	S	960	835	
L8 (LA)	N	791	120	0.2497
	S	782	100	
L10 (UK)	N	103	115	0.005344
	S	131	85	

Supplementary Table 10. The median d_N/d_S values for 1133 genes within the *mat* chromosome SR region common to all lineages of *N. tetrasperma.* The p-value was obtained from a one-sided Mann-Whitney U test (MWU) for L5, L7, L9 and L10. A two-sided MWU test was used with L1 and L8.

**Lineage**[1]	**Median d_N/d_S**[2]		**P-value**
	mat A	***mat a***	
L1 (LA)	0.121	0.125	0.9028
L5 (NZ)[3]	**0.094**	0.119	0.03645
L7 (LA)	0.135	**0.118**	0.055
L8 (LA)	0.131	0.129	0.9138
L9 (LB)	0.137	**0.103**	0.0007221
L10 (UK)	**0.095**	0.119	0.008701

[1] Lineages L4, L5a and L6 were excluded from the analyses due to low divergence with other lineages.

[2] The d_N/d_S values marked in bold indicate the introgressed *mat* chromosome for the lineage being considered.

[3] Values used here from the L10 *mat a* branch.

Supplementary Table 11. Numbers of synonymous substitution in the SR region. Subsitution were catergorised as optimal or non-optimal. Counts of synonymous substitions resulting in switches from optimal to non-optimal codons (*OP → NOP)* and non-optimal to optimal codons were (*NOP → OP)* were determined as described in the methods section. Neutral substitutions include *OP → OP and NOP → NOP* switches.

Lineage	Synonymous subsitutions	*mat* A	*mat* a	Ratio	P-value
**L1 (LA)**	*OP → NOP*[1]	1719	1566	1.10	0.54 (FET)
	NOP → OP[1]	1025	968	1.06	0.23 (FET)
	Neutral[2]	2677	2371	1.13	<0.0001 (ST)
**L4 (MX)**	*OP → NOP*	2253	2148	1.05	0.03 (FET)
	NOP → OP	1268	1260	1.01	0.74 (FET)
	Neutral	3309	3383	0.98	0.37 (ST)
**L5 (NZ)**	*OP → NOP*	3212	3097	1.04	0.26 (FET)
	NOP → OP	2035	1823	1.12	0.09 (FET)
	Neutral	4975	4698	1.06	0.005 (ST)
**L6 (HI)**	*OP → NOP*	3606	3496	1.03	0.82 (FET)
	NOP → OP	2311	2095	1.11	0.004 (FET)
	Neutral	5307	5291	1.00	0.88 (ST)
**L7 (LA)**	*OP → NOP*	1950	2086	0.93	0.55 (FET)
	NOP → OP	1119	1194	0.94	0.49 (FET)
	Neutral	2972	3164	0.94	0.0002 (ST)
**L8 (LA)**	*OP → NOP*	1636	1814	0.90	0. 44 (FET)
	NOP → OP	935	997	0.94	0.93 (FET)
	Neutral	2536	2718	0.94	0.01 (ST)
**L9 (LB)**	*OP → NOP*	3145	3833	0.82	0.10 (FET)
	NOP → OP	1761	2567	0.69	<0.0001 (FET)
	Neutral	4779	6064	0.79	0.009 (ST)
**L10 (UK)**	*OP → NOP*	2557	2520	1.01	0.35 (FET)
	NOP → OP	1507	1439	1.05	0.35 (FET)
	Neutral	3940	3827	1.04	0.20 (ST)

[1] Statistical significance differences in *OP → NOP* and *NOP → OP* was determined using a Fisher's Exact test (FET) with a contingency table containing the *OP → NOP* and Neutral, or *NOP → OP* and Neutral. Statistical significance differences between Neutral synonymous subsitutions was determined using a sign test (ST).

Supplementary Table 12. Information on genes from the SR and PAR region shared by all *N. tetrasperma* lineages sequenced and the heterothallic species used for the phylogenetic analysis presented in Fig 5.

Gene	Region	Alignment Length (bp)
*ad-9**	SR	693
*al-1**	SR	839
*arg-1**	SR	1646
cys-9	SR	1050
eth-1	SR	982
*lys-4**	SR	777
phr	PAR	1917
*ro-10**	PAR	662

*New gene sequence data for heterothallic species not represented in Sun *et al.* (2012)
